# GeneSetCart: assembling, augmenting, combining, visualizing, and analyzing gene sets

**DOI:** 10.1093/gigascience/giaf025

**Published:** 2025-04-10

**Authors:** Giacomo B Marino, Stephanie Olaiya, John Erol Evangelista, Daniel J B Clarke, Avi Ma'ayan

**Affiliations:** Mount Sinai Center for Bioinformatics, Department of Pharmacological Sciences, Department of Artificial Intelligence and Human Health, Icahn School of Medicine at Mount Sinai, New York, NY, 10029, USA; Mount Sinai Center for Bioinformatics, Department of Pharmacological Sciences, Department of Artificial Intelligence and Human Health, Icahn School of Medicine at Mount Sinai, New York, NY, 10029, USA; Mount Sinai Center for Bioinformatics, Department of Pharmacological Sciences, Department of Artificial Intelligence and Human Health, Icahn School of Medicine at Mount Sinai, New York, NY, 10029, USA; Mount Sinai Center for Bioinformatics, Department of Pharmacological Sciences, Department of Artificial Intelligence and Human Health, Icahn School of Medicine at Mount Sinai, New York, NY, 10029, USA; Mount Sinai Center for Bioinformatics, Department of Pharmacological Sciences, Department of Artificial Intelligence and Human Health, Icahn School of Medicine at Mount Sinai, New York, NY, 10029, USA

**Keywords:** Venn, UpSet, SuperVenn, gene set intersection, Geneshot, integrative analysis, Alexander disease, aging, exercise, Chrome extension

## Abstract

Converting multiomics datasets into gene sets facilitates data integration that leads to knowledge discovery. Although there are tools developed to analyze gene sets, only a few offer the management of gene sets from multiple sources. GeneSetCart is an interactive web-based platform that enables investigators to gather gene sets from various sources; augment these sets with gene–gene coexpression correlations and protein–protein interactions; perform set operations on these sets such as union, consensus, and intersection; and visualize and analyze these gene sets, all in one place. GeneSetCart supports the upload of single or multiple gene sets, as well as fetching gene sets by searching PubMed for genes comentioned with terms in publications. Venn diagrams, heatmaps, Uniform Manifold Approximation and Projection (UMAP) plots, SuperVenn diagrams, and UpSet plots can visualize the gene sets in a GeneSetCart session to summarize the similarity and overlap among the sets. Users of GeneSetCart can also perform enrichment analysis on their assembled gene sets with external tools. All gene sets in a session can be saved to a user account for reanalysis and sharing with collaborators. GeneSetCart has a gene set library crossing feature that enables analysis of gene sets created from several National Institutes of Health Common Fund programs. For the top overlapping sets from pairs of programs, a large language model (LLM) is prompted to propose possible reasons for the high overlap. Using this feature, two use cases are presented. In addition, users of GeneSetCart can produce publication-ready reports from their uploaded sets. Text in these reports is also supplemented with an LLM. Overall, GeneSetCart is a useful resource enabling biologists without programming expertise to facilitate data integration for hypothesis generation.

## Background

The abstraction of biological and biomedical knowledge into gene sets has proven to be useful for data integration and reuse [1]. High-dimensional omics datasets are commonly converted into gene set libraries, which are collections of individually annotated gene sets [2]. Gene sets can be created from many types of omics resources such as genomics, proteomics, epigenomics, metabolomics, and high-throughput drug and gene knockout, knockdown, and overexpression screening. Such gene sets can be differentially expressed genes from transcriptomics studies such as RNA sequencing (RNA-seq), targets of transcription factors from epigenomics experiments such as chromatin immunoprecipitation followed by sequencing (ChIP-seq), protein complexes from mass spectrometry proteomics, genes that harbor mutations or deletions that lead to a human or a mouse phenotype from genomics studies, genes belonging to a pathway or a biological process based on literature curation, or marker genes that define specific cell types within a tissue [3].

Several web-based platforms facilitate the analysis of gene sets in the cloud. Most of these efforts focus on gene set enrichment analysis while others provide access to set operations and data visualizations. Enrichment analysis tools compute the significance of the overlap between an input gene set and background gene sets organized into gene set libraries. Enrichr is one example of a widely used gene set enrichment analysis tool that computes overrepresentation against a wide array of gene set libraries created from a multitude of sources [4]. Another leading tool, gProfiler, has the added functionality of ID conversion, multiorganism support, and support for single-nucleotide polymorphism (SNP) enrichment analysis, but compared to Enrichr, gProfiler supports enrichment analysis with only a few selected gene set libraries [5]. One of the first, and still one of the leading platforms in this domain, is the Database for Annotation, Visualization, and Integrated Discovery (DAVID) [6].

DAVID also supports ID conversion and additionally enables the combination of sets with the union operation. Other key widely used enrichment analysis platforms are WebGestalt [7], GSEA [1], ToppGene [8], and Metascape [9]. However, most of these systems do not provide users with accounts where they can save and manage their gene sets and apply other types of analyses and visualizations on these sets. One example of a gene set management system is Flame [10]. Flame has the ability to upload multiple sets and perform combinatorial functional enrichment analysis for multiple organisms using different enrichment analysis tools that include aGOtool [11], gProfiler [5], WebGestalt [12], and Enrichr [4]. Flame also supports different gene identifiers, SNPs, and uploading free text that can be mined for genes and proteins using named entity recognition (NER) for a specific organism. However, Flame does not have the ability to save gene sets in a user account. Another application, called Intervene [13], is a command-line tool that visualizes intersection across gene sets with Venn diagrams, UpSet plots, and clustered heatmaps. Intervene can generate five types of Venn diagrams: classical, Chow–Ruskey, Edwards, squares, and battleship. The web application Evenn [14] can be used to generate Venn diagrams, including classical and Edwards, Euler proportional diagrams, UpSet plots, Flower plots, and Venn network diagrams. GeneOverlap [15] is an R package that can visualize gene set overlaps with heatmaps. Altogether, these resources are widely used by experimental biologists that study gene sets, but these applications lack many features and are not always user-friendly.

GeneSetCart is a web-based application to manage the analysis of collections of gene sets. The platform provides access to some of the key functions implemented for the tools and services mentioned above but also has some unique features that set it apart. Users of GeneSetCart can assemble gene sets from multiple sources, including their own gene sets, annotated sets extracted from omics resources, and gene sets associated with biomedical terms from PubMed. Users can then augment these gene sets with related genes based on protein–protein interactions (PPIs), coexpression, and comentions networks; visualize the overlap between their gene sets; send the gene set for analysis with external tools; and produce reports that contain the results of the analysis and visualizations by selecting from a collection of visualization methods and downstream analysis tools. Additionally, GeneSetCart enables the storage and sharing of gene sets in user accounts. GeneSetCart also has a Chrome extension that only works when users visit the Gene Expression Omnibus (GEO) [16], PubMed, and PubMed Central (PMC) websites. The extension uses the Rummagene [17] and RummaGEO [18] resources to assist users with extracting gene sets from GEO studies and PubMed articles and load them for analysis by GeneSetCart.

## Data Description

### Assembling gene set from different sources

**Figure 1:**
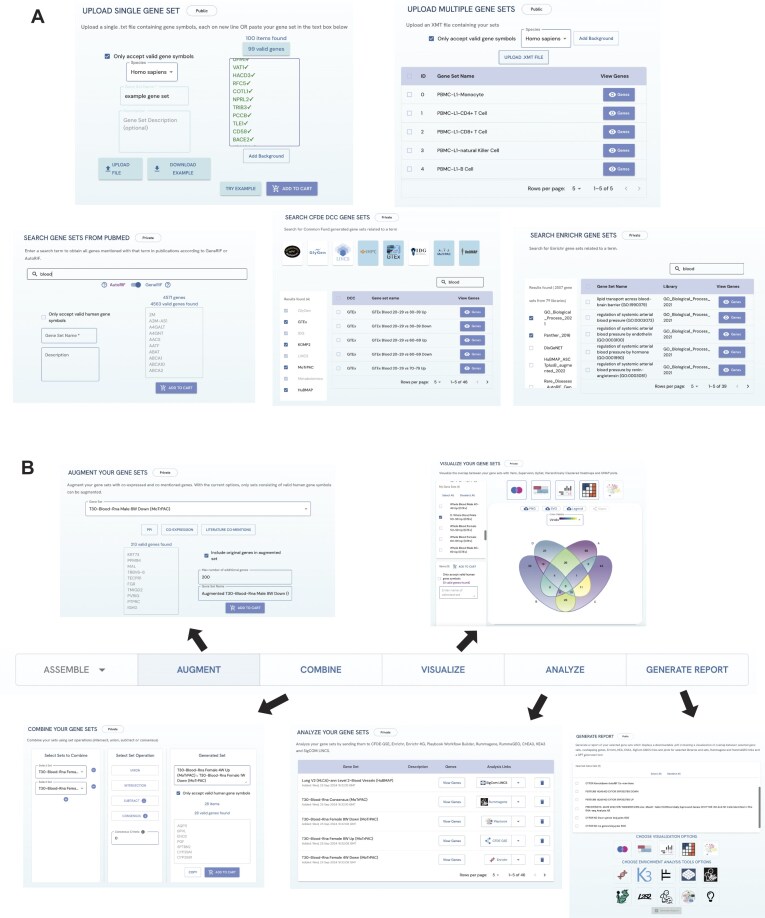
GeneSetCart gene set input and analysis features. (A) Users can upload a single gene set or enter a gene set in a text area; upload a .gmt file containing multiple gene sets; search PubMed for any term, and then the returned PMIDs are converted into a gene set based on GeneRIF or AutoRIF; fetch gene sets created from data collected by Common Fund programs; and fetch gene sets from Enrichr gene set libraries related to an input term. (B) The Augment feature allows augmentation of a gene set with coexpressed genes or PPI. The Combine features provides mechanisms to perform set operations on gene sets to create new sets. The Visualize page contain tools to visualize the overlap among sets, including Venn diagrams, UpSet plots, SuperVenn, heatmaps, and UMAP visualization. The Analyze page permits users to submit their gene sets to a collection of external tools, and the Report page provides means to generate reports using a selected set of sets and tools for performing the analysis.

Users of GeneSetCart can assemble gene sets from multiple sources (Fig. 1A). The first source is user-submitted gene sets. Users can upload gene sets using a few methods. Users can upload a .txt file containing a single gene set or a .gmt file containing multiple gene sets. Next, gene sets can be created using a PubMed search. This feature uses the Geneshot [19] application programming interface (API) to convert PMIDs into genes based on comentions in publications. Gene–publication associations are sourced from GeneRIF. Another source for assembling gene sets is from Enrichr [4]. Enrichr has over 500,000 annotated gene sets organized into >530 gene set libraries. The Enrichr gene set search functionality in GeneSetCart enables users to query Enrichr’s metadata for finding gene sets based on their description given any search term. Once matching gene sets are found, they can be added to the GeneSetCart shopping cart. Similarly, GeneSetCart has a collection of gene sets created from National Institutes of Health (NIH) Common Fund programs. These gene sets can be queried based on their description and added to the cart in a similar manner as the way Enrichr gene sets are fetched. The final method to add gene sets into the GeneSetCart is via a Chrome extension. The GeneSetCart Chrome extension available from the Google Chrome Store was developed with JavaScript and HTML. The extension enables users to add gene sets found in the Rummagene [17] and RummaGEO [18] databases when users visit the PubMed, PMC, or the GEO NCBI websites. The Rummagene database holds gene sets extracted from supplementary materials of publications deposited into PMC, while the RummaGEO gene sets are extracted from differential expression signatures automatically computed from the uniformly aligned RNA-seq GEO studies available from the ARCHS4 resource [20]. For a given set, users can choose to only include valid Entrez gene symbols from the NCBI Gene database or include any identifiers. This flexibility makes GeneSetCart applicable to handle other set types such as drugs, variants, and metabolites.

## Methods

### Gene names validation and mapping

All gene sets uploaded to GeneSetCart (RRID:SCR_026349) are converted into official NCBI gene symbols when the gene validation toggle is selected. Synonyms from other ontologies such as Ensembl and HGNC are supported and converted to official NCBI gene symbols. Users may select from 18 species to perform gene name conversion and validation. However, since most downstream tools support only human gene symbols, a copy of the set with valid mappable human gene symbols is also stored. Additionally, the gene conversion functionality includes correcting common Excel date conversion errors, where gene names are mistakenly converted to dates.

### Gene set augmentation

After assembling gene sets from various sources, users can expand their sets by adding similar genes to each set using the Augmentation step (Fig. 1B). The gene set augmentation feature takes a gene set from the shopping cart and based on the selected option for augmentation (coexpression, literature comentions, and PPIs), the gene set is expanded with additional relevant genes. The coexpression option uses the Geneshot (RRID:SCR_017582) [19] API, which returns genes that are coexpressed with the genes in the original set. The coexpressed genes are determined based on gene–gene coexpression correlations calculated from the processed data in ARCHS4 (RRID:SCR_015683) [20]. The literature comentions option also calls the Geneshot [19] API but with the GeneRIF gene–gene similarity matrix parameter selected. The related genes are those mostly comentioned with the input gene set based on GeneRIF comentions in publications. The PPI option uses the Genes2Networks (G2N) [21] API. This API endpoint returns genes that directly interact with genes in the sets based on known PPIs. The PPIs in Genes2Networks are assembled from BioGRID (RRID:SCR_007393) [22], BioPlex (RRID:SCR_016144) [23], IntAct (RRID:SCR_006944) [24], MINT (RRID:SCR_001523) [25], PPID [26], iRefWeb (RRID:SCR_008118) [27], Stelzl et al. [28], and a few other PPI resources. To construct PPI subnetworks, Genes2Networks [21] is utilizing the PPI from these databases and the shortest-path algorithm with a maximum path length of 2 between 2 seed genes. For each of these 3 augmentation options, users can specify the maximum number of genes to be added by the augmentation with the default set to 200 genes. There is also an option to decide whether to include the original genes from the set or only include the augmented genes.

### Combining gene sets

GeneSetCart (RRID:SCR_026349) has an interface that facilitates users to select sets and combine them to generate additional sets using 1 of 4 set operations options: union, intersection, consensus, and subtract (Fig. 1B). The union operation returns a single gene set containing all elements that are in each of the selected gene sets. The intersection option returns a single set composed of all elements that belong to all the selected gene sets. The consensus option returns a single gene set composed of genes that appear in at least N of the selected sets, where N is a number specified by the user. The subtract operation subtracts the union of other selected sets from the first selected set.

### Visualization of the overlap among selected gene sets

GeneSetCart (RRID:SCR_026349) can visualize the overlap between selected gene sets in the cart with several interactive and static publication-ready plots, including Venn diagrams, SuperVenn diagrams, UpSet plots, hierarchically clustered heatmaps, and UMAP plots [29] (Fig. 1B). The Venn diagrams support the visualization of up to 5 sets and use the Reaviz React library [30]. This library renders React elements using the Data-Driven Documents (D3) JavaScript library [31]. For visualization of the overlap of more sets, UpSet plots are created by rendering React Javascript XML (JSX) elements with D3 using the D3-UpSet library [32]. The SuperVenn plots are created with the React-SuperVenn library [33], which is an interactive React implementation of the Python supervenn library [34]. For the hierarchically clustered heatmap, we calculate the Jaccard similarity between all gene sets and then use the Seaborn clustermap function with default parameters to create the heatmap [32]. To create the UMAP plots, we compute the inverse document frequency matrix of all gene sets using the term frequency inverse document frequency (TF-IDF) vectorizer function from the Scikit-learn Python package [35]. The Scanpy Python package [36] is then used to create the UMAP embeddings of the TF-IDF values, which are visualized as a scatterplot created using React JSX and D3. In the UMAP, each point represents a gene set. Points on the UMAP can be colored based on 2 options. The default coloring option applies the Leiden algorithm [37] to the TF-IDF vectors, and thus the gene set points are colored based on their assigned cluster. For the user-assigned option, the application enables users to assign gene sets to groups by uploading a .csv file mapping each gene set to its desired group. The default UMAP parameters used are minDist=0.1, spread=1, nNeighbors=15, randomState=42. Sliders are provided for users to change these parameters. The Venn, SuperVenn, UpSet, and UMAP plots are interactive. Users can view the number of genes in each gene set in the visualization by clicking the region in the plot that represents the gene set. The selected gene sets can be added to the cart for further downstream analysis. The generated visualizations are also available for download as publication-ready portable network graphics (PNG) and scalable vector graphics (SVG) images. The URL of the plots can be shared to view the plots again directly from the GeneSetCart website.

### Gene set enrichment analysis with external tools

Users of GeneSetCart (RRID:SCR_026349) can submit the gene sets in their cart for analysis with external tools. There are currently 11 tools to choose from: Enrichr (RRID:SCR_001575) [4], Enrichr-KG [38], Rummagene [17], RummaGEO [18], ChIP-X Enrichment Analysis 3 (ChEA3) (RRID:SCR_005403) [39], Kinase Enrichment Analysis 3 (KEA3) [40], SigCom LINCS (RRID:SCR_022275) [41], LINCS L1000 Signature Search (L2S2), Common Fund Data Ecosystem Gene Set Enrichment (CFDE-GSE), Playbook Workflow Builder (PWB) [42], and PFOCRummage (Fig. 1B). ChEA3 performs transcription factor (TF) enrichment analysis to rank TFs associated with a given gene set. Similarly, KEA3 performs kinase enrichment analysis to find upstream kinases whose putative substrates are overrepresented in an input gene set. SigCom LINCS and L2S2 perform signature similarity search for mimickers and reversers compounds and single-gene knockouts by querying the gene set against a collection of more than one million gene expression signatures collected by the L1000 assay for the Library of Integrated Network-Based Cellular Signatures (LINCS) program [43]. The PWB uses the input gene set as an entry point for creating interactive workflows. PFOCRummage facilitates the search of the gene set against the gene sets collected for the Pathway Figure Optical Character Recognition project [44,45]. A selected gene set in GeneSetCart (RRID:SCR_026349) is sent to one of these external tools using the tools’ APIs. The tool returns a persistent URL link to the given analysis of the gene set with the selected tool. This URL is used to visualize the enrichment analysis results in the browser. Since some of these external tools have the option to include a background for the enrichment analysis [46], users of GeneSetCart can upload such background set or select from various provided backgrounds.

### Generating hypotheses with a large language model

For each significant gene set crossing pair, the user can add the overlapping genes to the GeneSetCart (RRID:SCR_026349) shopping cart, send the overlapping genes to Enrichr (RRID:SCR_001575) [4] for enrichment analysis, and generate a hypothesis that provides a possible explanation for the highly significant overlap between the gene sets pair. Such hypotheses are formed based on a textual description of each gene set and significantly enriched terms collected from Enrichr. To create a textual description of each gene set, we designed templates for each Common Fund Data Ecosystem (CFDE) gene set library. The template has the experimental and computational procedures used to create each gene set from each Common Fund program. We prompt the GPT-4o model from OpenAI to parse the terms associated with each gene set and place the appropriate parts of the term in the place fillers of the template. In order to provide the model more context to generate meaningful hypotheses, we perform gene set enrichment with the overlapping genes using the GO Biological Processes (RRID:SCR_002811) [47], WikiPathways (RRID:SCR_002134) [48], MGI Mammalian Phenotype (RRID:SCR_012953) [49], and the GWAS Catalog (RRID:SCR_012745) [50] Enrichr (RRID:SCR_001575) libraries. The top 5 enriched terms from each of these libraries are added to the model prompt. The final prompt instructs the model to generate a hypothesis describing the reason for the high overlap between the 2 gene sets based on the 2 sets, the templates, and the enriched terms. User-uploaded sets can be compared to CFDE gene sets and to each other. When users select exactly 2 sets in the report mode, an option to form large language model (LLM)–driven hypotheses about the possible relationship between the 2 sets is provided.

### Generating reports with GeneSetCart

GeneSetCart (RRID:SCR_026349) can produce downloadable persistently accessible reports provided in HTML and PDF formats. These reports contain the same visualization and analysis modules available from the visualize and analyze sections. These functions are applied to a selected small collection of gene sets from the user’s session. Users can select up to 5 gene sets, analysis tools, and visualization modules to include in the report. Once executed, the reports are displayed in the browser in HTML format. The reports also have a button to download the report as a PDF file. Users can share reports with a provided persistent URL. The executed reports also have a link to the GeneSetCart session, a listing of the included gene sets and their size, a table of contents, and the selected visualization and analysis modules, as well as figure and table legends. Reports also contain text automatically generated by an LLM. If the user selects exactly 2 sets for the report, they can enable a hypothesis generation feature. To generate hypotheses, GeneSetCart (RRID:SCR_026349) utilizes the same elements as described for the CFDE gene set crossing feature. The time that it takes to generate reports depends on the number of gene sets and the tools the user selects, as well as the size of the selected sets (Supplementary Fig. S1). Overall, the reports feature of GeneSetCart (RRID:SCR_026349) enables users to easily export and share the most relevant results from their gene set analyses.

### The GeneSetCart web-based interface implementation

The GeneSetCart web application is implemented in Typescript with the NextJS version 14 framework. A PostgreSQL database is used to store all user, gene set, and crossing data, while a Prisma ORM is used to query the database. User authentication is applied with NextAuth.js, enabling users to log into the site using their Keycloak authentication hosted by the CFDE Workbench web portal [51].

## Analyses

### The GeneSetCart user interface

The user interface of GeneSetCart starts with a homepage where users can begin a session by clicking on the “Start Here” button. This initiates a session, and the user is navigated to the Assemble page. In the top right corner of the site, users can log into their user account. Sessions created while a user is logged are automatically saved to that user’s account such that they can be reinstated. All sessions initiated when a user is not logged are public sessions that can still be shared via a persistent URL. Sessions created by a logged-in user, however, can be set to be private or public. By default, all sessions are private. Users own the intellectual property of their sets, and these sets will not be shared or published without the user’s consent. Users can share their sessions with their colleagues by making them public. Making sessions public is still hidden from most users because URLs to public sessions are also not shared with others by GeneSetCart and not indexed by search engines. Thus, the site provides means for users to communicate the results of their analyses to others while providing some level of privacy. While the platform provides a secure store for gene sets that may come from an investigator’s unpublished experimental results, investigators should be aware that we cannot guarantee 100% security of the GeneSetCart database. In addition, when gene sets are sent to third-party applications, such as LLM services, there is a chance that such gene sets are saved by these third-party applications.

### Crossing CFDE gene set libraries

**Figure 2:**
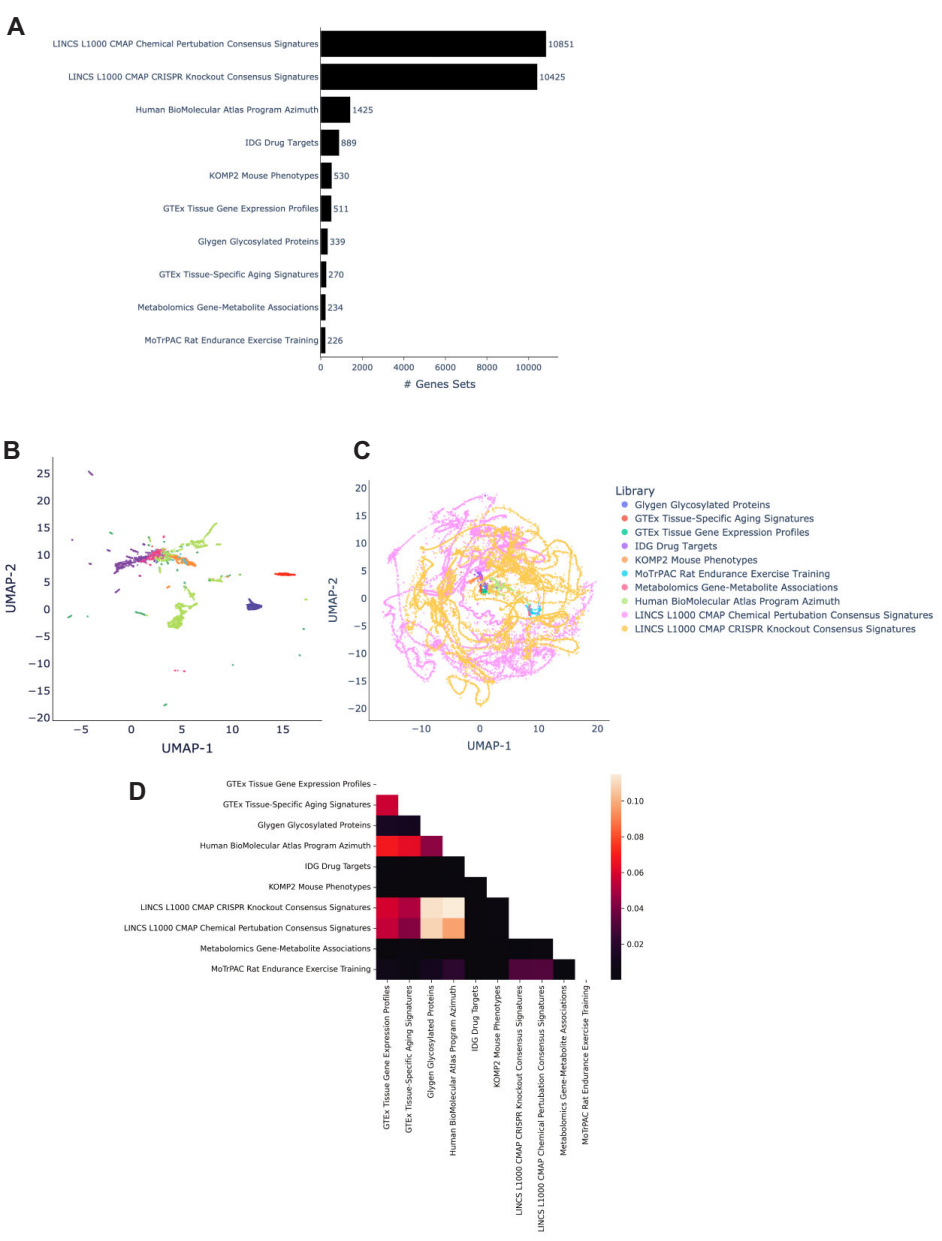
CFDE gene set libraries included in GeneSetCart. (A) Gene set sizes created from various NIH Common Fund programs. (B) UMAP plot of IDF vectors of gene sets across Common Fund gene set libraries excluding LINCS. (C) UMAP plot of IDF vectors of gene sets across all NIH Common Fund gene set libraries. (D) Percentage of significantly overlapping crossing pairs across all possible combinations of gene set libraries.

The gene set library crossing feature in GeneSetCart provides access to tables that rank pairs of gene sets created from data collected by 8 NIH Common Fund (CF) supported programs. Currently, there are 10 gene set libraries in GeneSetCart created from these 8 programs—LINCS [43], Illuminating the Druggable Genome (IDG) [52], Metabolomics Workbench [53], the Knockout Mouse Phenotyping Program (KOMP2) [54], the Genotype-Tissue Expression (GTEx) [55], Glygen [56], Human BioMolecular Atlas Program (HuBMAP) [57], and the Molecular Transducers of Physical Activity Consortium (MoTrPAC) [58] (Fig. 2A). To rank gene set pairs from different CF programs, the significance of the overlap between set pairs is computed with the Fisher exact test computed with the SciPy Python package [59]. The crossed gene set pairs with a *P* value of <0.001 are retained. The gene sets from the different CF libraries are visualized with UMAP plots (Fig. 2B, C). Some gene set libraries form a singular cluster such GTEx aging signatures (red), GTEx tissue expression profiles (green), IDG drug targets (purple), and KOMP2 (orange) while other libraries have multiple clusters such as the HubMAP Azimuth library (yellow). The LINCS libraries do not form clusters but instead show looping strings likely because of many gene sets with small overlap. The crossed gene sets of each library are first characterized by the percentage of significant crossing pairs (*P* < 0.001). A lower triangle heatmap visualizes these percentages for each library pair (Fig. 2D). Unsurprisingly, we observed that libraries sourced from the same CF program, for example, GTEx aging signatures and GTEx tissue expression, display the greatest overlap (Supplementary Fig. S2). Additionally, the gene set libraries sourced from the LINCS program display high overlap with the gene set libraries created from GTEx, GlyGen, and HuBMAP. This might be because most of these gene sets are created from transcriptomics. The gene set libraries created from KOMP2 and Metabolomics have the least overlap with other libraries. This might be because these gene sets were created from data collected by methods that are unique to each program.

### Case study 1: Shared pathways implicated in aging and exercise by crossing gene set libraries created from GTEx and MoTrPAC

**Figure 3:**
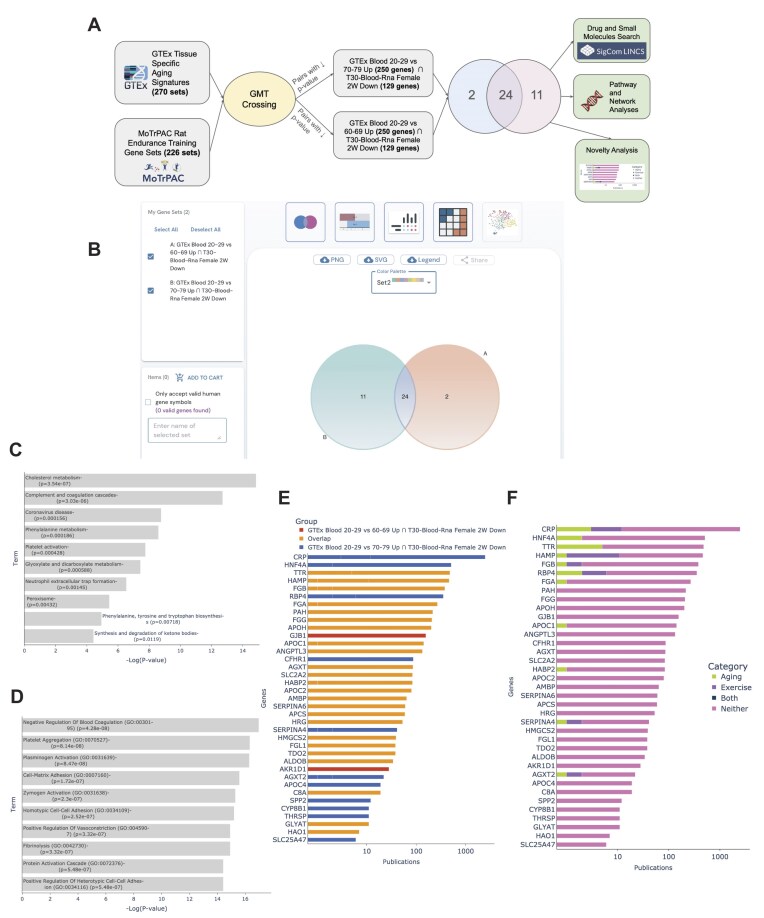
Exploring shared pathways implicated in aging and exercise from blood. (A) Workflow diagram depicting the analysis steps of cross the gene set libraries created from GTEx and MoTrPAC. (B) Screenshot from the Venn diagram visualization created by GeneSetCart of the overlap between the top 2 crossing results of GTEx Aging Signatures vs. MoTrPAC Rat Endurance Training libraries. (C) Enrichment analysis of the 24 overlapping genes from the top 2 crossing results with the KEGG 2021 Human gene set library in Enrichr. (D) Enrichment analysis of the top 24 overlapping genes with the GO Biological Processes library in Enrichr. (E) Publication count associated with all identified genes colored by set source. (F) Publication for each gene colored by comentions with the terms aging, exercise, both, and neither.

Aging is a risk factor for many common chronic diseases [60,61] such as type 2 diabetes [62], cardiovascular disease [63], and neurological disorders such as Alzheimer’s [64] and Parkinson’s diseases [65]. Moderate exercise [66] is widely accepted as a mechanism to promote overall health and aid in the prevention of aging-related diseases [67]. To investigate the common biological underpinnings that accompany both aging and exercise, as well as discover genes that are induced or repressed due to exercise and aging, we crossed the GTEx aging signatures with the MoTrPAC rat endurance training gene sets [68] (Fig. 3A, Supplementary Fig. S3). In total, 346 gene set pairs have a significant overlap (*P* < 0.001, Fisher exact test). The top 2 gene set pairs (GTEx Blood 20–29 vs. 60–69 Up ∩ T30-Blood-Rna Female 2 W Down and GTEx Blood 20–29 vs. 70–79 Up ∩ T30-Blood-Rna Female 2 W Down) have 35 (*P* = 6.52e-38) and 26 (*P* = 1.05e-24) overlapping genes, respectively. The “GTEx Blood 20–29 vs. 60–69 Up” gene set contains genes that are upregulated when comparing the blood of subjects aged 20–29 to those aged 60–69, and similarly the “GTEx Blood 20–29 vs. 70–79 Up” gene set contains genes that are upregulated when comparing the blood of subjects aged 20–29 to those aged 70–79. The “T30-Blood-Rna Female 2 W Down” gene set consists of genes that are downregulated in the blood of rats after 2 weeks of endurance training. Next, we added these 2 gene sets to GeneSetCart and used the intersection set operation to discover that these 2 sets share 24 genes in common (Fig. 3B).

Enrichment analysis applied to these 24 overlapping genes using Enrichr [4], with the default background, and with a background set of genes expressed only in blood [46] using ARCHS4 as the background atlas [20] found enriched pathways related to immune response, blood coagulation, and lipid metabolism, which are all processes known to be affected by both aging and physical activity (Fig. 3C, D). Some enriched terms are blood-related processes that are particularly known to undergo significant changes with aging and exercise such as blood coagulation and fibrinolysis. It is well known that aging is associated with increased plasma levels of many proteins related to coagulation [69]. Additionally, an acute bout of exercise is also associated with a transient increase in blood coagulation, whereas moderate exercise is known to enhance blood fibrinolytic activity without activation of coagulation mechanisms, while heavy exercise induces simultaneous activation of blood fibrinolysis and coagulation [70]. Notably, coagulation and fibrinolysis genes are upregulated due to aging in human blood and downregulated in rat blood due to aerobic long-term exercise. Additionally, blood lipids are a likely source of human aging and exercise biomarkers with blood lipid levels, including total cholesterol, low- and high-density lipoprotein cholesterol, and triglycerides changing in specific ways with age [71, 72] while endurance exercise induces fat oxidation [73]. These results are in concordance with the notion that aerobic exercise can attenuate some of the hallmarks of aging [74]. The identified genes can become biomarkers and potential therapeutic targets for exercise mimickers. Some of the identified genes are already well-known targets and biomarkers, while others are completely unknown. A novelty assessment of the gene was performed by comparing the number of publications each gene has on PubMed (Fig. 3E). We found that 65% of the genes contained in those sets are associated with fewer than 100 publications listed on PubMed, with 2 genes (HAO1 and SLC25A47) having fewer than 10 publications. We also found that out of the 37 genes (union of both sets), only 11 are previously comentioned with the terms aging, exercise, or both. These genes are CRP, HNF4A, TTR, HAMP, FGB, RBP4, FGA, APOC1, HABP2, SERPINA4, and AGXT2 (Fig. 3F). These results suggest that many of the identified overlapping genes are understudied in the context of both aging and exercise, and as such, they provide hypotheses that warrant further exploration.

### Case study 2: Exploring novel targets for Alexander’s disease

**Figure 4:**
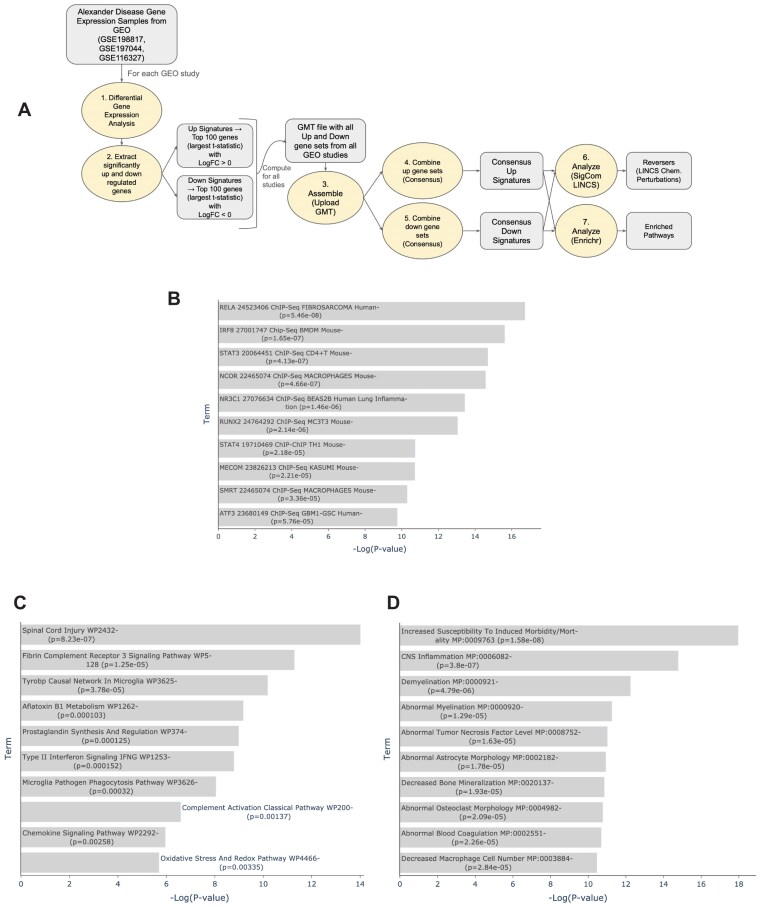
Investigating mechanisms in Alexander’s disease. (A) Use case workflow and analysis steps. (B) Top 10 enriched terms from ChEA 2022 library of consensus up signature (*n* = 3) gene set in Enrichr. (C) Top 10 enriched terms from WikiPathway 2023 Mouse library of consensus up signature (*n* = 3) gene set in Enrichr. (D) Top 10 enriched terms from MGI Mammalian Phenotype Level 4 2024 library of consensus up signature (*n* = 3) gene set in Enrichr.

Alexander’s disease (AxD) is a rare neurodegenerative disease caused by a mutation in the *GFAP* gene, which codes for the glial fibrillary acidic protein (GFAP) [75]. The GFAP protein supports the formation of myelin sheaths in normal physiology, but in AxD, the gain-of-function mutation in the *GFAP* gene causes the protein product to accumulate. Instead of helping maintain myelin sheaths, the extra GFAP causes damage to the myelin. The overexpression of GFAP in animal models also results in the appearance and accumulation of Rosenthal fibers (RFs), protein aggregates in the cytoplasm of astrocytes [76], in subpial and white matter central nervous system areas, which have typically high *GFAP* expression. Other than RF buildup, astrocytes in AxD also have abnormal cell shape and function. The GEO is a major open biomedical research repository for transcriptomics and other omics datasets that currently contains millions of gene expression samples from tens of thousands of studies collected by research laboratories from around the world [77]. Here, we use the GeneSetCart pipeline to analyze gene sets created by comparing gene expression samples obtained from GEO of wild type (WT) or controls to AxD samples (Fig. 4A).

To obtain the AxD disease signatures, we perform differential gene expression analysis on RNA-seq gene expression samples from 3 GEO studies that compare control or wild-type cells and tissues to AxD samples (GSE198817, GSE197044, GSE116327) [78]. GSE198817 contains gene expression samples from the hippocampus and corpus callosum tissue of Gfap^+/+^, Gfap^+^/R236H, and mGFAPTg170-2 transgenic mice. The GSE197044 study has RNA-seq profiles from hippocampus and corpus callosum tissue of male Gfap^+^/R236H and Gfap^+/+^ mice in FVB/N-Tac at 8 weeks of age, and the GSE116327 study has profiles from healthy controls and AxD patients’ induces pluripotent stem cell (iPSC)-derived astrocytes and postmortem brain tissues. Differentially expressed genes between healthy controls and disease samples for each study are computed using the limma method [79]. This analysis was performed with the bulk RNA-seq analysis pipeline appyter [80]. The up and down genes were converted into gene sets. These gene sets were uploaded to GeneSetCart for further integrative analysis. Using the GeneSetCart Combine feature, consensus up and down sets were created. Choosing the consensus criteria of 3, the consensus up set has 65 genes and the consensus down set has 20 genes. These up and down consensus sets were submitted to SigCom LINCS [41] to identify potential drugs and preclinical small molecules that may reverse the disease gene expression changes in different cell lines. We also perform gene set enrichment analysis on the consensus up and down sets with Enrichr [4] (Fig. 4B–D).

The consensus upregulated genes are enriched for transcription factors known to regulate immune response and inflammation. The top 3 transcription factors from the ChEA [39] analysis are RELA, IRF8, and STAT3 (*P* < 0.0001, Fisher exact test) (Fig. 4B). Consistent with inflammation and AxD, the top enriched WikiPathways [48] pathway is Spinal Cord Injury WP2432 (*P* = 8.234e-7) with the 6 overlapping genes: CXCL10, CCND1, CCL2, CXCL1, VIM, and GFAP (Fig. 4C). The most profound results from the enrichment analysis come from the MGI Mouse Phenotypes library with the top 4 most enriched terms: Increased Susceptibility to Induced Morbidity/Mortality MP:0009763 (*P* = 1.576e-8), CNS Inflammation MP:0006082 (*P* = 3.795e-7), Demyelination MP:0000921 (*P* = 0.000004793), and Abnormal Myelination MP:0000920 (*P* = 0.00001292). The knockout mice of the overlapping genes with these terms could serve as AxD disease models due to the shared phenotype. GFAP only overlaps with genes from the Abnormal Myelination MP:0000920 phenotype together with TYROBP, PTPRC, ADGRG6, and TLR2 (Fig. 4D). When querying Rummagene [17] with the consensus up genes, the brain inflammation signature is further confirmed. Several of the top matching sets in Rummagene are from brain inflammation studies with 2 studies about prion disease [81, 82], suggesting potentially similar mechanisms between prion disease and AxD. The consensus downregulated genes are enriched for terms related to brain tissues and cell types. Specifically, markers for astrocytes are the top enriched terms from the gene set libraries created from CellMarker [83], Tabula Muris [84], PanglaoDB [85], and Allen Brain Atlas 10x scRNA [86]. This observation is also supported by a RummaGEO [18] query that returned matching gene sets from studies titled as follows: RNA-seq of human astrocytes GSE73721, Regionally specified human pluripotent stem cell-derived astrocytes GSE133489, and CROP-seq of hiPSC-derived astrocytes from GSE182307 and GSE182309. Altogether, the AxD down gene set appears to be related to astrocyte cell type specification, supporting the observed phenotype of defective astrocytes in AxD.

## Discussion

GeneSetCart is a user-friendly platform designed to help biomedical researchers to explore knowledge about gene sets. The platform provides mechanisms to upload, compare, combine, visualize, save, share, manage, and analyze collections of gene sets. In addition, users of GeneSetCart can perform enrichment analyses using a variety of tools via the use of their APIs. Additional tools that accept gene sets could be added. The application builds on the functionality of many existing gene set analysis tools. For example, Geneshot [19] is used to convert PubMed searches into gene sets, and Genes2Networks [21] and ARCHS4 [20] are used to expand a gene set with PPI and gene–gene coexpression correlations, respectively. The gene set expansion functionality provides predictions about additional genes that may be involved in the same function as a given gene set. Such a gene set expansion approach can be used to form novel hypotheses and point to new targets. Machine learning methods could be employed to improve this functionality in the future. GeneSetCart also employs LLMs to form hypotheses about the overlap between pairs of gene sets. Typically, such hypotheses state obvious general concepts that can be gleaned from examining the shared genes and enriched terms. However, LLM helps with articulating the most apparent patterns, and in some cases, the LLM can provide surprising explanations. As LLMs improve, their ability to reason is expected to further improve this feature. Users of GeneSetCart should also use caution when reading the LLM hypotheses because it is plausible for the LLM to make logical mistakes. One other limitation of GeneSetCart is that it is designed to handle a relatively small collection of gene sets. Users with hundreds of gene sets should seek other analysis with other tools, methods, and platforms.

### Potential implications

GeneSetCart was created to promote the reuse of NIH Common Fund datasets. So far, we have created 10 gene set libraries from 8 Common Fund programs. It is expected that additional libraries from more programs will be added to the system once such data become available. One of the use cases provided in this article and on the GeneSetCart site crosses gene sets from 2 NIH Common Fund programs: GTEx [55] and MoTrPAC [58]. The use case shows that crossing the GTEx aging gene sets, created from profiling postmortem human tissues, with tissues from rats collected by MoTrPAC after prolonged aerobic exercise produced interesting insights. However, this is just one example. Many other crossings and overlaps are made possible by GeneSetCart and remain to be explored. For example, exercise mimickers and aging reversers can be identified by crossing the gene set libraries created from the NIH Common Fund LINCS [43] with those created from MoTrPAC and GTEx.

## Availability of Source Code and Requirements

Project name: GeneSetCart

Project homepage: https://genesetcart.cfde.cloud/

GitHub link: https://github.com/MaayanLab/GeneSetCart

Chrome extension: https://chromewebstore.google.com/detail/genesetcart/dahaedghigbofibfadgedahlekhphmbd

Operating system(s): Platform independent

Programming language: TypeScript, Python

License: GPL-3.0

Software Heritage PID: swh:1:snp:95562a973a8c179a12e91f3fcbeba6e8182f301f

Bio.tools Unique Identifier: biotools:genesetcart

RRID:SCR_026349

## Supplementary Material

giaf025_Supplemental_Files

giaf025_GIGA-D-24-00490_Original_Submission

giaf025_GIGA-D-24-00490_Revision_1

giaf025_GIGA-D-24-00490_Revision_2

giaf025_Response_to_Reviewer_Comments_Original_Submission

giaf025_Response_to_Reviewer_Comments_Revision_1

giaf025_Reviewer_1_Report_Original_Submission Mark Ziemann, PhD -- 12/5/2024

giaf025_Reviewer_1_Report_Revision_1 Mark Ziemann, PhD -- 2/4/2025

giaf025_Reviewer_2_Report_Original_Submission Weidong Tian -- 12/23/2024

giaf025_Reviewer_2_Report_Revision_1 Weidong Tian -- 2/3/2025

## Data Availability

Snapshots of the code are available in Software Heritage [87]. All additional datasets used in this research can be found on the homepage [88].

## Additional Files

**Supplementary Fig. S1**. API response time (*n* = 5) as a function of gene set size for all linked analysis tools.

**Supplementary Fig. S2**. *P* value distribution of significant library crossing results for each library. (A) Glygen Glycosylated Proteins vs. rest. (B) GTEx Aging Signatures vs. rest. (C) GTEx Tissue Gene Expression Profiles vs. rest. (D) IDG Drug Targets vs. rest. (E) KOMP2 Mouse Phenotypes vs. rest. (F) MoTrPAC Rat Endurance Training vs. rest. (G) Metabolomics Workbench Metabolites. (H) HuBMAP azimuth 2023 augmented. (I) LINCS L1000 chemical perturbation consensus signatures. (J) LINCS L1000 CRISPR knockout consensus signatures.

**Supplementary Fig. S3**. Hypothesis generation for crossing the MoTrPAC exercise signatures with the GTEx tissue aging signatures. (A) The CFDE GMT Crossing page results of crossing the GTEx tissue-specific aging signatures with the MoTrPAC Rat Endurance Training libraries. (B) Overlapping genes with the lowest *P* values for gene set pair overlap. (C) GPT-4 generated hypothesis of lowest *P* value gene set pair.

## Abbreviations

API: application programming interface; ARCHS4: all RNA-seq and ChIP-seq sample signature search; AxD: Alexander disease; CF: Common Fund; CFDE: Common Fund Data Ecosystem; CFDE-GSE: Common Fund Data Ecosystem Gene Set Enrichment; ChEA3: ChIP-X enrichment analysis 3; CRISPR: clustered regularly interspaced short palindromic repeats; CROP-seq: CRISPR droplet sequencing; D3: data-driven documents; DAVID: Database for Annotation, Visualization, and Integrated Discovery; G2N: genes to networks; GeneRIF: gene reference into function; GEO: Gene Expression Omnibus; GFAP: glial fibrillary acidic protein; GO: Gene Ontology; GSEA: gene set enrichment analysis; GTEx: genotype-tissue expression; GWAS: genome-wide association study; HGNC: HUGO Gene Nomenclature Committee; HuBMAP: Human Biomolecular Atlas Program; HUGO: Human Genome Organization; JSX: JavaScript XML; KEA3: kinase enrichment analysis 3; KOMP2: knockout mouse phenotyping program 2; L2S2: LINCS L1000 signature search; LINCS: Library of Integrated Network-Based Cellular Signatures; LLM: large language model; MotrPAC: Molecular Transducers of Physical Activity Consortium; NCBI: National Center for Biotechnology Information; NER: named entity recognition; NIH: National Institutes of Health; OCR: optical character recognition; ORM: object relational mapping; PFOCRummage: pathway figure OCR rummage; PMC: PubMed Central; PNG: portable network graphics; PPI: protein–protein interaction; PWB: Playbook Workflow Builder; RF: Rosenthal fibers; scRNA: single-cell RNA; SNP: single-nucleotide polymorphism; SVG: scalable vector graphics; TF: transcription factor; TF-IDF: term frequency inverse document frequency; UMAP: uniform manifold projection; URL: uniform resource locator; WT: wild type; XML: extensible markup language.

## References

[1] Subramanian A, Tamayo P, Mootha VK, et al. Gene set enrichment analysis: a knowledge-based approach for interpreting genome-wide expression profiles. Proc Natl Acad Sci U S A. 2005;102:15545–50. 10.1073/pnas.0506580102. 16199517 PMC1239896

[2] Ma'ayan A, Rouillard AD, Clark NR, et al. Lean Big data integration in systems biology and systems pharmacology. Trends Pharmacol Sci. 2014;35:450–60. 10.1016/j.tips.2014.07.001. 25109570 PMC4153537

[3] Rouillard AD, Gundersen GW, Fernandez NF, et al. The harmonizome: a collection of processed datasets gathered to serve and mine knowledge about genes and proteins. Database. 2016;2016:baw100. 10.1093/database/baw100. PMC4930834 27374120

[4] Chen EY, Tan CM, Kou Y, et al. Enrichr: interactive and collaborative HTML5 gene list enrichment analysis tool. BMC Bioinf. 2013;14:128. 10.1186/1471-2105-14-128. PMC3637064 23586463

[5] Reimand J, Kull M, Peterson H, et al. g:Profiler—a web-based toolset for functional profiling of gene lists from large-scale experiments. Nucleic Acids Res. 2007;35:W193–200. 10.1093/nar/gkm226. 17478515 PMC1933153

[6] Sherman BT, Hao M, Qiu J, et al. DAVID: a web server for functional enrichment analysis and functional annotation of gene lists (2021 update). Nucleic Acids Res. 2022;50:W216–21. 10.1093/nar/gkac194. 35325185 PMC9252805

[7] Elizarraras JM, Liao Y, Shi Z, et al. WebGestalt 2024: faster gene set analysis and new support for metabolomics and multi-omics. Nucleic Acids Res. 2024;52:W415–21. 10.1093/nar/gkae456. 38808672 PMC11223849

[8] Chen J, Bardes EE, Aronow BJ, et al. ToppGene Suite for gene list enrichment analysis and candidate gene prioritization. Nucleic Acids Res. 2009;37:W305–11. 10.1093/nar/gkp427. 19465376 PMC2703978

[9] Zhou Y, Zhou B, Pache L, et al. Metascape provides a biologist-oriented resource for the analysis of systems-level datasets. Nat Commun. 2019;10:1523. 10.1038/s41467-019-09234-6. PMC6447622 30944313

[10] Karatzas E, Baltoumas FA, Aplakidou E, et al. Flame (v2.0): advanced integration and interpretation of functional enrichment results from multiple sources. Bioinformatics. 2023;39(8):btad490. 10.1093/bioinformatics/btad490. 37540207 PMC10423032

[11] Schölz C, Lyon D, Refsgaard JC, et al. Avoiding abundance bias in the functional annotation of post-translationally modified proteins. Nat Methods. 2015;12:1003–4. 10.1038/nmeth.3621. 26513550

[12] Zhang B, Kirov S, Snoddy J. WebGestalt: an integrated system for exploring gene sets in various biological contexts. Nucleic Acids Res. 2005;33:W741–48. 10.1093/nar/gki475. 15980575 PMC1160236

[13] Khan A, Mathelier A. Intervene: a tool for intersection and visualization of multiple gene or genomic region sets. BMC Bioinf. 2017;18:287. 10.1186/s12859-017-1708-7. PMC5452382 28569135

[14] Yang M, Chen T, Liu Y-X, et al. Visualizing set relationships: eVenn's comprehensive approach to Venn diagrams. Imeta. 2024;3:e184. 10.1002/imt2.184. 38898979 PMC11183158

[15] Shen L . GeneOverlap: an R package to test and visualize gene overlaps. *R Package*. bioconductor.statistik.tu-dortmund.de. 2016. 10.18129/B9.bioc.GeneOverlap. (Accessed 3 April 2024).

[16] Clough E, Barrett T, Wilhite SE, et al. NCBI GEO: archive for gene expression and epigenomics data sets: 23-year update. Nucleic Acids Res. 2024;52:D138–44. 10.1093/nar/gkad965. 37933855 PMC10767856

[17] Clarke DJB, Marino GB, Deng EZ, et al. Rummagene: massive mining of gene sets from supporting materials of biomedical research publications. Commun Biol. 2024;7:482. 10.1038/s42003-024-06177-7. 38643247 PMC11032387

[18] Marino GB, Clarke DJB, Lachmann A, et al. RummaGEO: automatic mining of human and mouse gene sets from GEO. Patterns (N Y). 2024;5:101072. 10.1016/j.patter.2024.101072. 39569206 PMC11573963

[19] Lachmann A, Schilder BM, Wojciechowicz ML, et al. Geneshot: search engine for ranking genes from arbitrary text queries. Nucleic Acids Res. 2019;47:W571–77. 10.1093/nar/gkz393. 31114885 PMC6602493

[20] Lachmann A, Torre D, Keenan AB, et al. Massive mining of publicly available RNA-seq data from human and mouse. Nat Commun. 2018;9:1366. 10.1038/s41467-018-03751-6. 29636450 PMC5893633

[21] Berger SI, Posner JM, Ma'ayan A. Genes2Networks: connecting lists of gene symbols using mammalian protein interactions databases. BMC Bioinf. 2007;8:372. 10.1186/1471-2105-8-372. PMC2082048 17916244

[22] Breitkreutz B-J, Stark C, Tyers M. The GRID: the General Repository for Interaction datasets. Genome Biol. 2003;4:R23. 10.1186/gb-2003-4-3-r23. PMC153463 12620108

[23] Huttlin EL, Ting L, Bruckner RJ, et al. The BioPlex network: a systematic exploration of the human interactome. Cell. 2015;162:425–40. 10.1016/j.cell.2015.06.043. 26186194 PMC4617211

[24] Hermjakob H, Montecchi-Palazzi L, Lewington C, et al. IntAct: an open source molecular interaction database. Nucleic Acids Res. 2004;32:D452–55. 10.1093/nar/gkh052. 14681455 PMC308786

[25] Zanzoni A, Montecchi-Palazzi L, Quondam M, et al. MINT: a molecular INTeraction database. FEBS Lett. 2002;513:135–40. 10.1016/S0014-5793(01)03293-8. 11911893

[26] Husi H, Grant SGN. Construction of a protein-protein interaction database (PPID) for synaptic biology. In: Neuroscience Databases. Boston (MA): Springer US, p. 51–62. 2003. 10.1007/978-1-4615-1079-6_4.

[27] Turner B, Razick S, Turinsky AL, et al. iRefWeb: interactive analysis of consolidated protein interaction data and their supporting evidence. Database (Oxford). 2010;2010:baq023. 10.1093/database/baq023. 20940177 PMC2963317

[28] Stelzl U, Worm U, Lalowski M, et al. A human protein-protein interaction network: a resource for annotating the proteome. Cell. 2005;122:957–68. 10.1016/j.cell.2005.08.029. 16169070

[29] McInnes L, Healy J. UMAP: uniform manifold approximation and projection for dimension reduction. arXiv.org. 2018. 10.48550/arXiv.1802.03426.

[30] Reaviz Contributors . Reaviz: data visualization library for React. GitHub. https://github.com/reaviz/reaviz.(Accessed 15 March 2024)

[31] Bostock M, Ogievetsky V, Heer J. D^3^: data-driven documents. IEEE Trans Vis Comput Graph. ​​​​​2011;17:2301–9. 10.1109/TVCG.2011.185. 22034350

[32] Ho C . D3-upset: an UpSet Plot in d3.Js. Toronto, CA: GitHub, https://github.com/chuntul/d3-upset. (Accessed 21 March 2024).

[33] Clarke DJ . react_supervenn. GitHub. https://github.com/MaayanLab/react-supervenn.(Accessed 12 February 2024)

[34] Fedor . supervenn: precise and easy-to-read multiple sets visualization in Python. GitHub. https://github.com/gecko984/supervenn. (Accessed 12 Feb 2024).

[35] Pedregosa F, Varoquaux G, Gramfort A, et al. Scikit-learn: machine learning in Python. J Mach Learn Res. 2011;12:2825–30. 10.5555/1953048.2078195.

[36] Wolf FA, Angerer P, Theis FJ. SCANPY: large-scale single-cell gene expression data analysis. Genome Biol. 2018;19:15. 10.1186/s13059-017-1382-0. 29409532 PMC5802054

[37] Traag VA, Waltman L, van Eck NJ. From Louvain to Leiden: guaranteeing well-connected communities. Sci Rep. 2019;9:5233. 10.1038/s41598-019-41695-z. 30914743 PMC6435756

[38] Evangelista JE, Xie Z, Marino GB, et al. Enrichr-KG: bridging enrichment analysis across multiple libraries. Nucleic Acids Res. 2023;51:W168–79. 10.1093/nar/gkad393. 37166973 PMC10320098

[39] Keenan AB, Torre D, Lachmann A, et al. ChEA3: transcription factor enrichment analysis by orthogonal omics integration. Nucleic Acids Res. 2019;47:W212–24. 10.1093/nar/gkz446. 31114921 PMC6602523

[40] Kuleshov MV, Xie Z, London ABK, et al. KEA3: improved kinase enrichment analysis via data integration. Nucleic Acids Res. 2021;49:W304–16. 10.1093/nar/gkab359. 34019655 PMC8265130

[41] Evangelista JE, Clarke DJB, Xie Z, et al. SigCom LINCS: data and metadata search engine for a million gene expression signatures. Nucleic Acids Res. 2022;50:W697–709. 10.1093/nar/gkac328. 35524556 PMC9252724

[42] Clarke DJB, Evangelista JE, Xie Z, et al. Playbook Workflow Builder: interactive construction of bioinformatics workflows from a network of microservices. PLOS Comput. Biol. 2025;21(4):e1012901. 10.1371/journal.pcbi.1012901. 40179105 PMC11967941

[43] Keenan AB, Jenkins SL, Jagodnik KM, et al. The Library of Integrated Network-Based Cellular Signatures NIH Program: system-level cataloging of human cells response to perturbations. Cell Syst. 2018;6:13–24. 10.1016/j.cels.2017.11.001. 29199020 PMC5799026

[44] Shin M-G, Pico A. Using published pathway figures in enrichment analysis and machine learning. BMC Genomics. 2023;24:713. 10.1186/s12864-023-09816-1. 38007419 PMC10676589

[45] Hanspers K, Riutta A, Summer-Kutmon M, et al. Pathway information extracted from 25 years of pathway figures. Genome Biol. 2020;21:273. 10.1186/s13059-020-02181-2. 33168034 PMC7649569

[46] Timmons JA, Szkop KJ, Gallagher IJ. Multiple sources of bias confound functional enrichment analysis of global -omics data. Genome Biol. 2015;16:186. 10.1186/s13059-015-0761-7. 26346307 PMC4561415

[47] Gene Ontology Consortium . Gene Ontology Consortium: going forward. Nucleic Acids Res. 2015;43:D1049–56. 10.1093/nar/gku1179. 25428369 PMC4383973

[48] Kutmon M, Riutta A, Nunes N, et al. WikiPathways: capturing the full diversity of pathway knowledge. Nucleic Acids Res. 2016;44:D488–94. 10.1093/nar/gkv1024. 26481357 PMC4702772

[49] Blake JA, Bult CJ, Eppig JT, et al. Mouse Genome Database Group. The Mouse Genome Database genotypes:phenotypes. Nucleic Acids Res. 2009;37:D712–19. 10.1093/nar/gkn886. 18981050 PMC2686566

[50] Sollis E, Mosaku A, Abid A, et al. The NHGRI-EBI GWAS Catalog: knowledgebase and deposition resource. Nucleic Acids Res. 2023;51:D977–85. 10.1093/nar/gkac1010. 36350656 PMC9825413

[51] Evangelista JE, Clarke DJB, Xie Z, et al. The CFDE Workbench: integrating metadata and processed data from Common Fund programs. bioRxiv. 10.1101/2025.02.04.636535. (Accessed 10 November 2024). 41506317

[52] Oprea TI, Bologa CG, Brunak S, et al. Unexplored therapeutic opportunities in the human genome. Nat Rev Drug Discov. 2018;17:317–32. 10.1038/nrd.2018.14. 29472638 PMC6339563

[53] Sud M, Fahy E, Cotter D, et al. Metabolomics Workbench: an international repository for metabolomics data and metadata, metabolite standards, protocols, tutorials and training, and analysis tools. Nucleic Acids Res. 2016;44:D463–70. 10.1093/nar/gkv1042. 26467476 PMC4702780

[54] Dickinson ME, Flenniken AM, Ji X, et al. High-throughput discovery of novel developmental phenotypes. Nature. 2016;537:508–14. 10.1038/nature19356. 27626380 PMC5295821

[55] GTE Consortium . The Genotype-Tissue Expression (GTEx) project. Nat Genet. 2013;45:580–85. 10.1038/ng.2653. 23715323 PMC4010069

[56] York WS, Mazumder R, Ranzinger R, et al. GlyGen: computational and informatics resources for glycoscience. Glycobiology. 2020;30:72–73. 10.1093/glycob/cwz080. 31616925 PMC7335483

[57] HuBMAP Consortium . The human body at cellular resolution: the NIH Human Biomolecular Atlas Program. Nature. 2019;574:187–92. 10.1038/s41586-019-1629-x. 31597973 PMC6800388

[58] Sanford JA, Nogiec CD, Lindholm ME, et al. Molecular Transducers of physical Activity Consortium (MoTrPAC): mapping the dynamic responses to exercise. Cell. 2020;181:1464–74. 10.1016/j.cell.2020.06.004. 32589957 PMC8800485

[59] Virtanen P, Gommers R, Oliphant TE, et al. SciPy 1.0: fundamental algorithms for scientific computing in Python. Nat Methods. 2020;17:261–72. 10.1038/s41592-019-0686-2. 32015543 PMC7056644

[60] Wick G, Jansen-Dürr P, Berger P, et al. Diseases of aging. Vaccine. 2000;18:1567–83. 10.1016/S0264-410X(99)00489-2. 10689131

[61] Saul D, Kosinsky RL. Epigenetics of aging and aging-associated diseases. Int J Mol Sci. 2021;22:401. 10.3390/ijms22010401. 33401659 PMC7794926

[62] Wilkerson HLC . Problems of an aging population. Am J Public Health Nations Health. 1947;37:177–88. 10.2105/AJPH.37.2.177. PMC1623303 18016480

[63] North BJ, Sinclair DA. The intersection between aging and cardiovascular disease. Circ Res. 2012;110:1097–108. 10.1161/CIRCRESAHA.111.246876. 22499900 PMC3366686

[64] Xia X, Jiang Q, McDermott J, et al. Aging and Alzheimer's disease: comparison and associations from molecular to system level. Aging Cell. 2018;17:e12802. 10.1111/acel.12802. 29963744 PMC6156542

[65] Reeve A, Simcox E, Turnbull D. Ageing and Parkinson's disease: why is advancing age the biggest risk factor?. Ageing Res Rev. 2014;14:19–30. 10.1016/j.arr.2014.01.004. 24503004 PMC3989046

[66] Caspersen CJ, Powell KE, Christenson GM. Physical activity, exercise, and physical fitness: definitions and distinctions for health-related research. Public Health Rep. 1985;100:126–31. 3920711 PMC1424733

[67] Fiuza-Luces C, Santos-Lozano A, Joyner M, et al. Exercise benefits in cardiovascular disease: beyond attenuation of traditional risk factors. Nat Rev Cardiol. 2018;15:731–43. 10.1038/s41569-018-0065-1. 30115967

[68] Schenk S, Sagendorf TJ, Many GM, et al. Physiological adaptations to progressive endurance exercise training in adult and aged rats: insights from The Molecular Transducers of Physical Activity Consortium (MoTrPAC). Function. 2024;5:zqae014. 10.1093/function/zqae014. 38984994 PMC11245678

[69] Tracy RP, Bovill EG. Thrombosis and cardiovascular risk in the elderly. Arch Pathol Lab Med. 1992;116:1307–12. 1456876

[70] El-Sayed MS, Sale C, Jones PG, et al. Blood hemostasis in exercise and training. Med Sci Sports Exerc. 2000;32:918–25. 10.1097/00005768-200005000-00007. 10795781

[71] Johnson AA, Stolzing A. The role of lipid metabolism in aging, lifespan regulation, and age-related disease. Aging Cell. 2019;18:e13048. 10.1111/acel.13048. 31560163 PMC6826135

[72] PS Collaboration, Lewington S, Whitlock G, et al. Blood cholesterol and vascular mortality by age, sex, and blood pressure: a meta-analysis of individual data from 61 prospective studies with 55,000 vascular deaths. Lancet. 2007;370:1829–39. 10.1016/s0140-6736(07)61778-4. 18061058

[73] Horowitz JF, Klein S. Lipid metabolism during endurance exercise. Am J Clin Nutr. 2000;72:558S–63S. 10.1093/ajcn/72.2.558S. 10919960

[74] Carapeto PV, Aguayo-Mazzucato C. Effects of exercise on cellular and tissue aging. Aging. 2021;13:14522–43. 10.18632/aging.203051. 34001677 PMC8202894

[75] Kuhn J, Cascella M. Alexander disease. 2023 Sep 4. Treasure Island (FL): StatPearls Publishing; 2025. 32965913

[76] Messing A, Head MW, Galles K, et al. Fatal encephalopathy with astrocyte inclusions in GFAP transgenic mice. Am J Pathol. 1998;152:391–98. 9466565 PMC1857948

[77] Barrett T, Wilhite SE, Ledoux P, et al. NCBI GEO: archive for functional genomics data sets—update. Nucleic Acids Res. 2013;41:D991–95. 10.1093/nar/gks1193. 23193258 PMC3531084

[78] Gammie SC, Messing A, Hill MA, et al. Large-scale gene expression changes in APP/PSEN1 and GFAP mutation models exhibit high congruence with Alzheimer's disease. PLoS One. 2024;19:e0291995. 10.1371/journal.pone.0291995. 38236817 PMC10796008

[79] Ritchie ME, Phipson B, Wu D, et al. limma powers differential expression analyses for RNA-sequencing and microarray studies. Nucleic Acids Res. 2015;43:e47. 10.1093/nar/gkv007. 25605792 PMC4402510

[80] Clarke DJB, Jeon M, Stein DJ, et al. Appyters: turning jupyter notebooks into data-driven web apps. Patterns (N Y). 2021;2:100213. 10.1016/j.patter.2021.100213. 33748796 PMC7961182

[81] Slota JA, Medina SJ, Frost KL, et al. Neurons and astrocytes elicit brain region specific transcriptional responses to prion disease in the Murine CA1 and thalamus. Front Neurosci. 2022;16:918811. 10.3389/fnins.2022.918811. 35651626 PMC9149297

[82] Crespo I, Roomp K, Jurkowski W, et al. Gene regulatory network analysis supports inflammation as a key neurodegeneration process in prion disease. BMC Syst Biol. 2012;6:132. 10.1186/1752-0509-6-132. 23068602 PMC3607922

[83] Zhang X, Lan Y, Xu J, et al. CellMarker: a manually curated resource of cell markers in human and mouse. Nucleic Acids Res. 2019;47:D721–28. 10.1093/nar/gky900. 30289549 PMC6323899

[84] TM Consortium . A single-cell transcriptomic atlas characterizes ageing tissues in the mouse. Nature. 2020;583:590–95. 10.1038/s41586-020-2496-1. 32669714 PMC8240505

[85] Franzén O, Gan L-M, Björkegren JLM. PanglaoDB: a web server for exploration of mouse and human single-cell RNA sequencing data. Database (Oxford). 2019;2019:baz046. 10.1093/database/baz046. 30951143 PMC6450036

[86] Shen EH, Overly CC, Jones AR. The Allen Human Brain Atlas: comprehensive gene expression mapping of the human brain. Trends Neurosci. 2012;35:711–14. 10.1016/j.tins.2012.09.005. 23041053

[87] Marino GB, Olaiya S, Evangelista JE, et al. GeneSetCart: assembling, augmenting, combining, visualizing, and analyzing gene sets (version 1) [Computer software]. Rocquencourt, France: Software Heritage, 2025. https://archive.softwareheritage.org/browse/snapshot/95562a973a8c179a12e91f3fcbeba6e8182f301f/directory/?origin_url=https://github.com/MaayanLab/GeneSetCart. (Accessed 27 March 2025). 10.1093/gigascience/giaf025 PMC11984350 40208796

[88] Marino GB, Olaiya S, Evangelista JE, et al. GeneSetCart: assembling, augmenting, combining, visualizing, and analyzing gene sets. https://genesetcart.cfde.cloud. (Accessed 10 February 2025). 10.1093/gigascience/giaf025 PMC11984350 40208796

