# Supplementary material for: GeneSetCart: assembling, augmenting, combining, visualizing, and analyzing gene sets
Source: Gigascience. 2025 Apr 10;14:giaf025. doi: 10.1093/gigascience/giaf025 (PMC11984350; doi:10.1093/gigascience/giaf025)
Supplement: giaf025_Supplemental_Files [file giaf025_supplemental_files.zip › figS3.pdf]

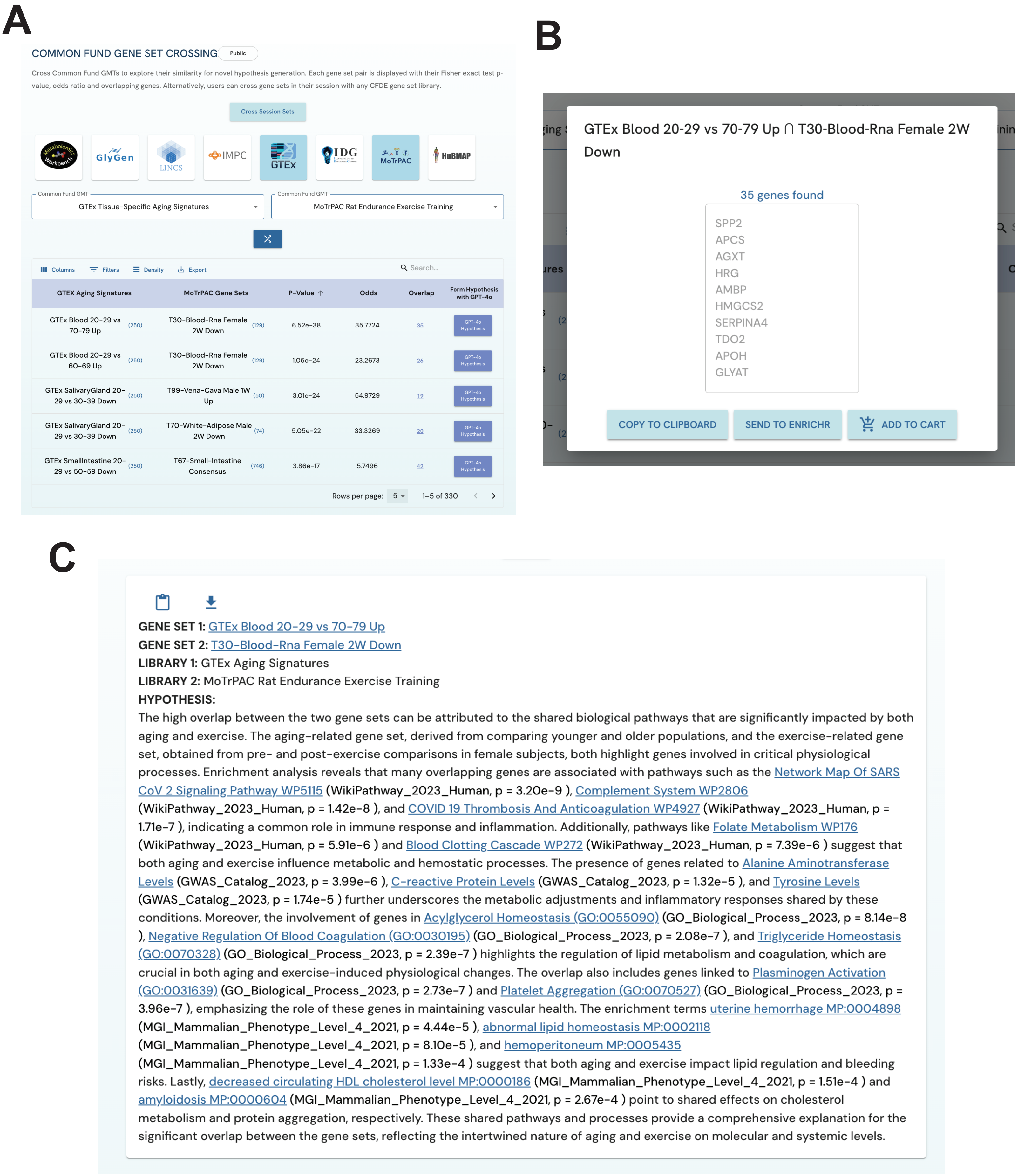
A
COMMON FUND GENE SET CROSSING
Public
Cross Common Fund GMTs to explore their similarity for novel hypothesis generation. Each gene set pair is displayed with their Fisher exact test p-value, odds ratio and overlapping genes. Alternatively, users can cross gene sets in their session with any CFDE gene set library.
Cross Session Sets
Metabolomics Workbench
GlyGen
LINCS
IMPC
GTEx
IDG
MoTrPAC
HuBMAP
Common Fund GMT
GTEx Tissue-Specific Aging Signatures
Common Fund GMT
MoTrPAC Rat Endurance Exercise Training
Columns
Filters
Density
Export
Search…
GTEX Aging Signatures
MoTrPAC Gene Sets
P-Value
Odds
Overlap
Form Hypothesis with GPT-4o
GTEx Blood 20-29 vs 70-79 Up (250) | T30-Blood-Rna Female 2W Down (129) | 6.52e-38 | 35.7724 | 35 | GPT-4o Hypothesis
GTEx Blood 20-29 vs 60-69 Up (250) | T30-Blood-Rna Female 2W Down (129) | 1.05e-24 | 23.2673 | 26 | GPT-4o Hypothesis
GTEx SalivaryGland 20-29 vs 30-39 Down (250) | T99-Vena-Cava Male 1W Up (50) | 3.01e-24 | 54.9729 | 19 | GPT-4o Hypothesis
GTEx SalivaryGland 20-29 vs 30-39 Down (250) | T70-White-Adipose Male 2W Down (74) | 5.05e-22 | 33.3269 | 20 | GPT-4o Hypothesis
GTEx SmallIntestine 20-29 vs 50-59 Down (250) | T67-Small-Intestine Consensus (746) | 3.86e-17 | 5.7496 | 42 | GPT-4o Hypothesis
Rows per page: 5
1–5 of 330
B
GTEx Blood 20-29 vs 70-79 Up ∩ T30-Blood-Rna Female 2W Down
35 genes found
SPP2
APCS
AGXT
HRG
AMBP
HMGCS2
SERPINA4
TDO2
APOH
GLYAT
COPY TO CLIPBOARD
SEND TO ENRICHR
ADD TO CART
C
GENE SET 1: GTEx Blood 20-29 vs 70-79 Up
GENE SET 2: T30-Blood-Rna Female 2W Down
LIBRARY 1: GTEx Aging Signatures
LIBRARY 2: MoTrPAC Rat Endurance Exercise Training
HYPOTHESIS:
The high overlap between the two gene sets can be attributed to the shared biological pathways that are significantly impacted by both aging and exercise. The aging-related gene set, derived from comparing younger and older populations, and the exercise-related gene set, obtained from pre- and post-exercise comparisons in female subjects, both highlight genes involved in critical physiological processes. Enrichment analysis reveals that many overlapping genes are associated with pathways such as the Network Map Of SARS CoV 2 Signaling Pathway WP5115 (WikiPathway_2023_Human, p = 3.20e-9), Complement System WP2806 (WikiPathway_2023_Human, p = 1.42e-8), and COVID 19 Thrombosis And Anticoagulation WP4927 (WikiPathway_2023_Human, p = 1.71e-7), indicating a common role in immune response and inflammation. Additionally, pathways like Folate Metabolism WP176 (WikiPathway_2023_Human, p = 5.91e-6) and Blood Clotting Cascade WP272 (WikiPathway_2023_Human, p = 7.39e-6) suggest that both aging and exercise influence metabolic and hemostatic processes. The presence of genes related to Alanine Aminotransferase Levels (GWAS_Catalog_2023, p = 3.99e-6), C-reactive Protein Levels (GWAS_Catalog_2023, p = 1.32e-5), and Tyrosine Levels (GWAS_Catalog_2023, p = 1.74e-5) further underscores the metabolic adjustments and inflammatory responses shared by these conditions. Moreover, the involvement of genes in Acylglycerol Homeostasis (GO:0055090) (GO_Biological_Process_2023, p = 8.14e-8), Negative Regulation Of Blood Coagulation (GO:0030195) (GO_Biological_Process_2023, p = 2.08e-7), and Triglyceride Homeostasis (GO:0070328) (GO_Biological_Process_2023, p = 2.39e-7) highlights the regulation of lipid metabolism and coagulation, which are crucial in both aging and exercise-induced physiological changes. The overlap also includes genes linked to Plasminogen Activation (GO:0031639) (GO_Biological_Process_2023, p = 2.73e-7) and Platelet Aggregation (GO:0070527) (GO_Biological_Process_2023, p = 3.96e-7), emphasizing the role of these genes in maintaining vascular health. The enrichment terms uterine hemorrhage MP:0004898 (MGI_Mammalian_Phenotype_Level_4_2021, p = 4.44e-5), abnormal lipid homeostasis MP:0002118 (MGI_Mammalian_Phenotype_Level_4_2021, p = 8.10e-5), and hemoperitoneum MP:0005435 (MGI_Mammalian_Phenotype_Level_4_2021, p = 1.33e-4) suggest that both aging and exercise impact lipid regulation and bleeding risks. Lastly, decreased circulating HDL cholesterol level MP:0000186 (MGI_Mammalian_Phenotype_Level_4_2021, p = 1.51e-4) and amyloidosis MP:0000604 (MGI_Mammalian_Phenotype_Level_4_2021, p = 2.67e-4) point to shared effects on cholesterol metabolism and protein aggregation, respectively. These shared pathways and processes provide a comprehensive explanation for the significant overlap between the gene sets, reflecting the intertwined nature of aging and exercise on molecular and systemic levels.
