# Supplementary figures and images for: GeneSetCart: assembling, augmenting, combining, visualizing, and analyzing gene sets
Source: Gigascience. 2025 Apr 10;14:giaf025. doi: 10.1093/gigascience/giaf025 (PMC11984350; doi:10.1093/gigascience/giaf025)

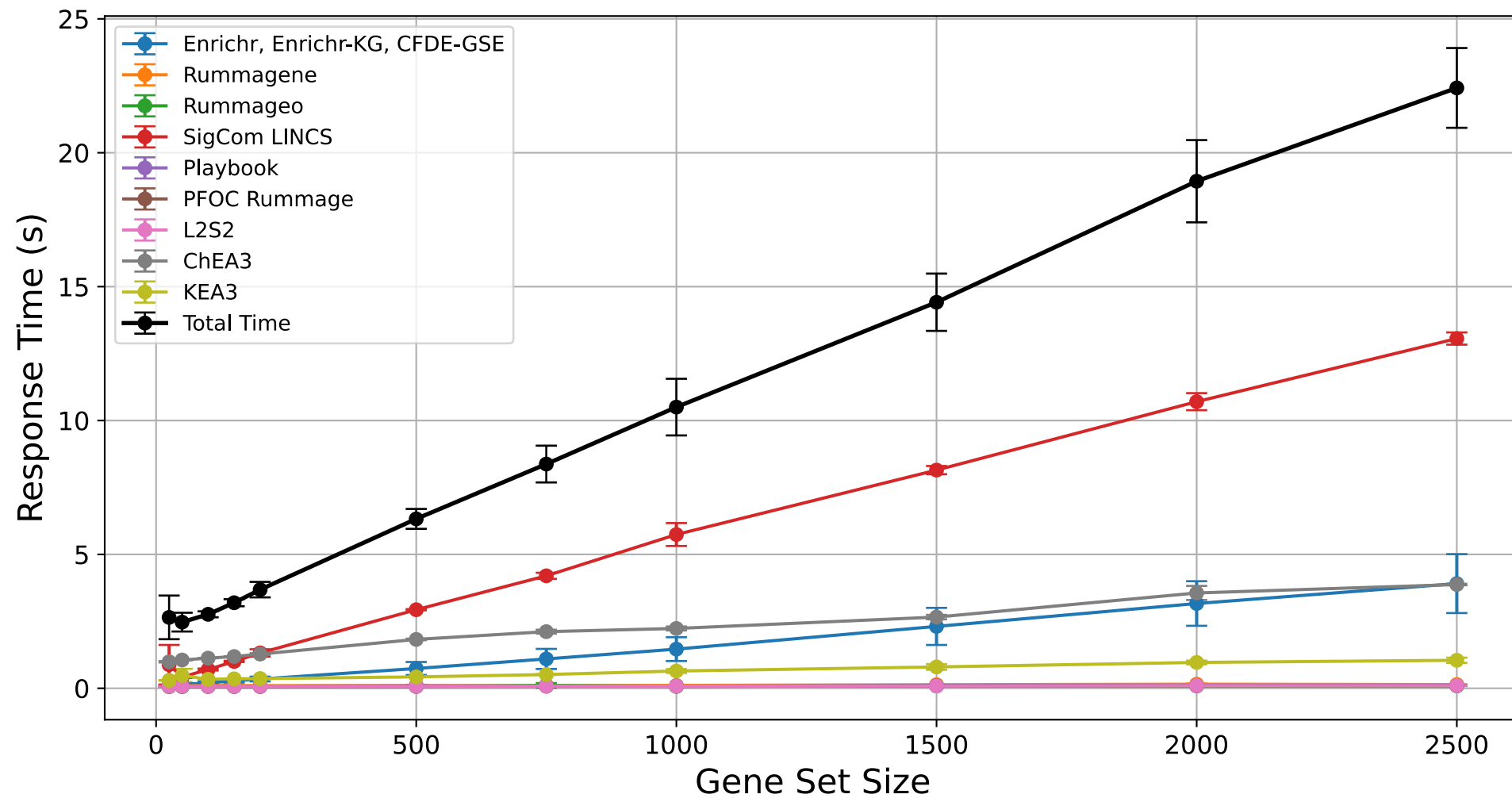

Supplement: giaf025_Supplemental_Files [file giaf025_supplemental_files.zip › figS1.pdf]

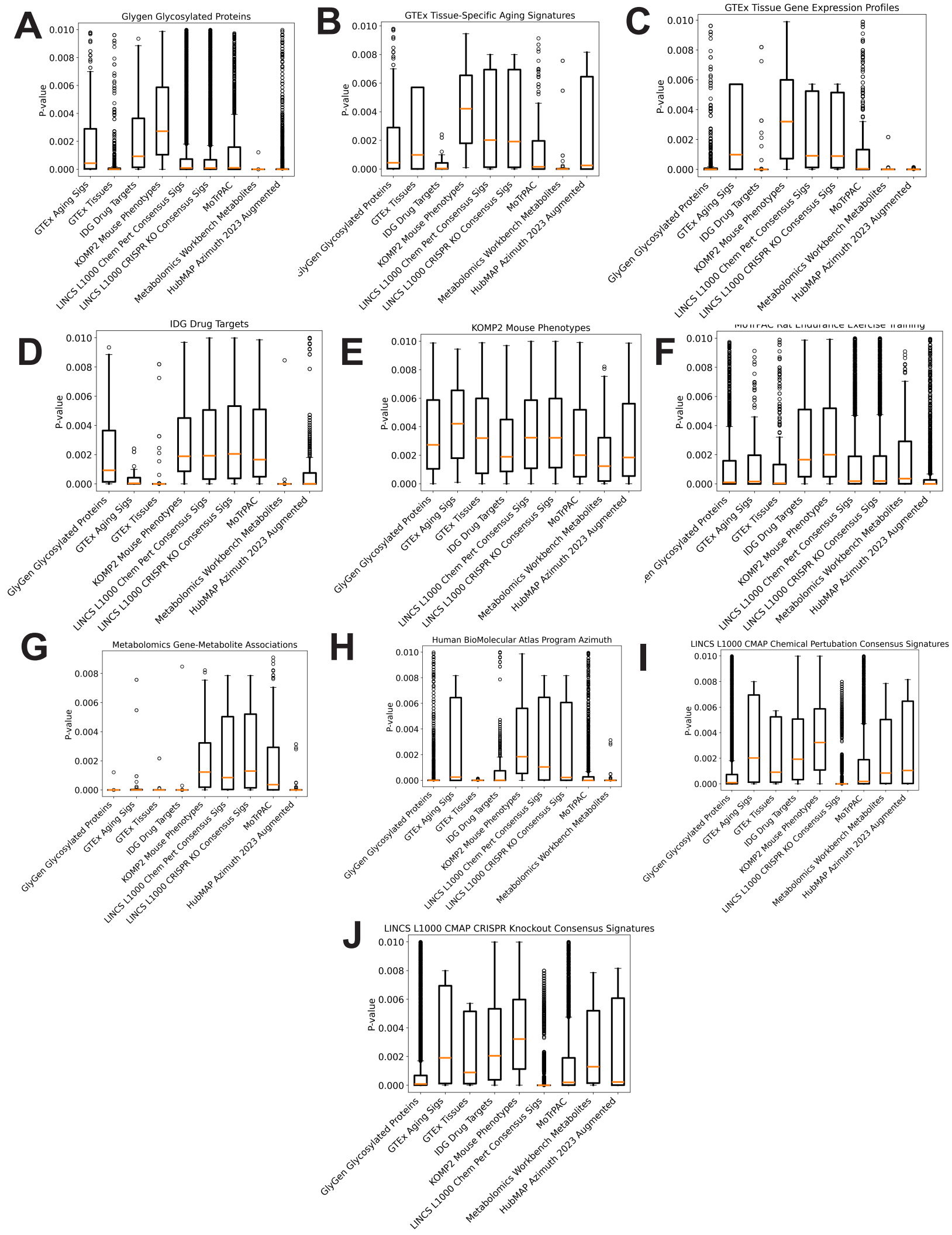

Supplement: giaf025_Supplemental_Files [file giaf025_supplemental_files.zip › figS2.pdf]
