# Supplementary material for: GeneSetCart: assembling, augmenting, combining, visualizing, and analyzing gene sets
Source: Gigascience. 2025 Apr 10;14:giaf025. doi: 10.1093/gigascience/giaf025 (PMC11984350; doi:10.1093/gigascience/giaf025)
Supplement: giaf025_GIGA-D-24-00490_Original_Submission [file giaf025_giga-d-24-00490_original_submission.pdf]

GigaScience

GeneSetCart: Assembling, Augmenting, Combining, Visualizing, and Analyzing Gene Sets

--Manuscript Draft--

**Manuscript Number:**	GIGA-D-24-00490
**Full Title:**	GeneSetCart: Assembling, Augmenting, Combining, Visualizing, and Analyzing Gene Sets
**Article Type:**	Research
**Funding Information:**	National Institute of Diabetes and Digestive and Kidney Diseases (R01DK131525) — Dr Avi Ma'ayan<br>National Institute of Diabetes and Digestive and Kidney Diseases (RC2DK131995) — Dr Avi Ma'ayan<br>NIH Office of the Director (OT2OD036435) — Dr Avi Ma'ayan<br>NIH Office of the Director (OT2OD030160) — Dr Avi Ma'ayan<br>Center for Biomedical Informatics and Information Technology, National Cancer Institute (U24CA264250) — Dr Avi Ma'ayan<br>Center for Biomedical Informatics and Information Technology, National Cancer Institute (U24CA271114) — Dr Avi Ma'ayan
**Abstract:**	Converting multi-omics datasets into gene sets facilitates data integration that leads to knowledge discovery. Although there are tools developed to analyze gene sets, only few offers management of gene sets from multiple sources. GeneSetCart is an interactive web-based platform that enables investigators to gather gene sets from various sources; augment these sets with gene-gene co-expression correlations and protein-protein interactions; perform set operations on these sets such as union, consensus, and intersection; and visualize and analyze these gene sets, all in one place. GeneSetCart supports the upload of single or multiple gene sets, as well as fetching gene sets by searching PubMed for genes co-mentioned with terms in publications. Venn diagrams, heatmaps, UMAP, SuperVenn diagrams, and UpSet plots can visualize the gene sets in a GeneSetCart session to summarize the overlap between the sets. Users of GeneSetCart can also perform enrichment analysis on their assembled gene sets with external tools. All gene sets in a session can be saved to a user account for re-analysis and sharing with collaborators. GeneSetCart has a gene-set-library crossing feature that enables analysis of gene sets created from several NIH Common Fund programs. For the top overlapping sets from pairs of programs, a large language model (LLM) is prompted to propose possible reasons for the high overlap. Using this feature, two use cases are presented. Finally, users of GeneSetCart can produce publication-ready reports from their uploaded sets. Overall, GeneSetCart is a useful resource for biologists without programming expertise to facilitate data integration for hypothesis generation.
**Corresponding Author:**	Avi Ma'ayan<br>Icahn School of Medicine at Mount Sinai<br>New York, NY UNITED STATES
**Corresponding Author Secondary Information:**	
**Corresponding Author's Institution:**	Icahn School of Medicine at Mount Sinai
**Corresponding Author's Secondary Institution:**	
**First Author:**	Giacomo B. Marino

Powered by Editorial Manager® and ProduXion Manager® from Aries Systems Corporation

**First Author Secondary Information:**	
**Order of Authors:**	Giacomo B. Marino
	Stephanie Olaiya
	John Erol Evangelista
	Daniel J. B. Clarke
	Avi Ma'ayan
**Order of Authors Secondary Information:**	
**Additional Information:**	
**Question**	**Response**
Are you submitting this manuscript to a special series or article collection?	No
**Experimental design and statistics**<br><br>Full details of the experimental design and statistical methods used should be given in the Methods section, as detailed in our Minimum Standards Reporting Checklist. Information essential to interpreting the data presented should be made available in the figure legends.<br><br>Have you included all the information requested in your manuscript?	Yes
**Resources**<br><br>A description of all resources used, including antibodies, cell lines, animals and software tools, with enough information to allow them to be uniquely identified, should be included in the Methods section. Authors are strongly encouraged to cite Research Resource Identifiers (RRIDs) for antibodies, model organisms and tools, where possible.<br><br>Have you included the information requested as detailed in our Minimum Standards Reporting Checklist?	Yes
**Availability of data and materials**<br><br>All datasets and code on which the	Yes

Powered by Editorial Manager® and ProduXion Manager® from Aries Systems Corporation

conclusions of the paper rely must be either included in your submission or deposited in publicly available repositories (where available and ethically appropriate), referencing such data using a unique identifier in the references and in the “Availability of Data and Materials” section of your manuscript.<br><br>Have you have met the above requirement as detailed in our Minimum Standards Reporting Checklist?	
GigaScience has policies and guidelines in place for the use of generative AI-writing tools such as ChatGPT. If you have used such writing tools to assist with writing the manuscript this must be declared and cited in the text. Authors should not list AI-writing tools and other AI-assisted technologies as an author or co-author and should acknowledge that they are fully responsible for text generated or refined by AI-writing tools.<p><br><br>A summary of use (particularly in the introduction or among methods) needs to be included at the end of the paper, and the outputs should also be included as a supplementary file hosted in GigaDB or other open repositories. Please read our guidelines for more information. <p><br><br>By submitting to GigaScience, you are aware of the journal's AI-writing tools policy, and if you have declared use of such tools below, you have acknowledged this where appropriate in your manuscript and have made a summary of use and outputs available. <p><br><b>AI-assisted writing tools have been used in the preparation of this manuscript?	No

Powered by Editorial Manager® and ProduXion Manager® from Aries Systems Corporation

Manuscript
Click here to access/download;Manuscript;GeneSetCart Manuscript 10312024-am.docx

GeneSetCart: Assembling, Augmenting, Combining, Visualizing, and Analyzing Gene Sets

Giacomo B. Marino[1], Stephanie Olaiya[1], John Erol Evangelista[1], Daniel J. B. Clarke[1], Avi Ma'ayan[1,*]

[1]Mount Sinai Center for Bioinformatics, Department of Pharmacological Sciences, Department of Artificial Intelligence and Human Health, Icahn School of Medicine at Mount Sinai, New York 10029, NY USA

*To whom correspondence should be addressed:
E-mail: avi.maayan@mssm.edu

Abstract

Converting multi-omics datasets into gene sets facilitates data integration that leads to knowledge discovery. Although there are tools developed to analyze gene sets, only few offers management of gene sets from multiple sources. GeneSetCart is an interactive web-based platform that enables investigators to gather gene sets from various sources; augment these sets with gene-gene co-expression correlations and protein-protein interactions; perform set operations on these sets such as union, consensus, and intersection; and visualize and analyze these gene sets, all in one place. GeneSetCart supports the upload of single or multiple gene sets, as well as fetching gene sets by searching PubMed for genes co-mentioned with terms in publications. Venn diagrams, heatmaps, UMAP, SuperVenn diagrams, and UpSet plots can visualize the gene sets in a GeneSetCart session to summarize the overlap between the sets. Users of GeneSetCart can also perform enrichment analysis on their assembled gene sets with external tools. All gene sets in a session can be saved to a user account for re-analysis and sharing with collaborators. GeneSetCart has a gene-set-library crossing feature that enables analysis of gene sets created from several NIH Common Fund programs. For the top overlapping sets from pairs of programs, a large language model (LLM) is prompted to propose possible reasons for the high overlap. Using this feature, two use cases are presented. Finally, users of GeneSetCart can produce publication-ready reports from their uploaded sets. Overall, GeneSetCart is a useful resource for biologists without programming expertise to facilitate data integration for hypothesis generation.

Keywords

Venn, UpSet, SuperVenn, gene set intersection, Geneshot, Integrative Analysis, Alexander Disease, Aging, Exercise, Chrome extension.

Background

The abstraction of biological and biomedical knowledge into gene sets has proven to be useful for data integration and reuse [1]. High dimensional omics datasets are commonly converted into gene set libraries which are collections of gene sets with annotations about the functions of each gene set in the library [2]. Gene sets can be created from many types of omics including genomics, proteomics, epigenomics, metabolomics, literature, and high-throughput drug and gene knockout, knockdown, and over-expression screening. Such gene sets can be differentially expressed genes from transcriptomics studies such as RNA-seq, targets of transcription factors from epigenomics experiments such as ChIP-seq, protein complexes from mass-spectrometry proteomics, genes that harbor mutations or deletions that lead to human or mouse phenotypes from genomics studies, genes belonging to a pathway or a biological process based on literature curation, or marker genes that define specific cell types within a tissue [3].

There are several web-based platforms that facilitate the analysis of gene sets in the cloud. Most of these efforts focus on gene set enrichment analysis while others provide access to set operations and data visualizations. Enrichment analysis tools compute the significance of an overlap between an input gene set and background gene sets organized into gene set libraries. Enrichr is one example of a widely used gene set enrichment analysis tool that computes over-representation against a wide array of gene-set libraries created from a multitude of sources [4]. Another leading tool called gProfiler has the added functionality of ID conversion, multi-organism support, and support for single-nucleotide polymorphism (SNPs) enrichment analysis, but compared to Enrichr, gProfiler supports enrichment analysis against only a few selected gene set libraries [5]. One of the first, and still one of the leading platforms in this domain, is the Database for Annotation, Visualization, and Integrated Discovery (DAVID) [6]. DAVID also supports ID conversion, and additionally enables the combination of sets with the union operation. Other key widely-used enrichment analysis platforms are WebGestalt [7], GSEA [1], ToppGene [8], and Metascape [9]. However, most of these systems do not provide users with accounts where they can save and manage their gene sets and apply other types of analyses and visualizations on these sets. One example of a gene set management system is Flame [10]. Flame has the ability to upload multiple sets and perform combinatorial functional enrichment analysis for multiple organisms using different enrichment analysis tools, namely aGOtool [11], gProfiler [5], WebGestalt [12], and Enrichr [4]. Flame also supports different gene identifiers, SNPs, and uploading free text that can be mined for genes and proteins using named entity recognition (NER) for a specific organism. Flame does not have the ability to save gene sets in a user account. Another application called Intervene [13] is a command line tool that visualizes intersection across gene sets with Venn diagrams, UpSet plots, and clustered heat maps. Intervene can generate five types of Venn diagrams: classical, Chow-Ruskey, Edwards, squares, and battleship. The web application Evenn [14] can be used to generate Venn diagrams including classical and Edwards, Euler proportional diagrams, UpSet plots, Flower plots, and Venn network diagrams. GeneOverlap [15] is an R package that can visualize gene set overlaps with heatmaps.

Altogether, these resources are widely used by experimental biologists that study gene set modules, but these applications lack many features and are not always user friendly.

GeneSetCart is a web-based application to manage the analysis of collections of gene sets. The platform provides access to some of the key functions implemented for the tools and services mentioned above, but also has some unique features that set it apart. Users of GeneSetCart can assemble gene sets from multiple sources including their own gene sets, annotated sets extracted from omics resources, and gene sets associated with biomedical terms from PubMed.
They can then augment these gene sets with related genes based on protein-protein interactions (PPI), co-expression, and co-mentions networks; visualize the overlap between their gene sets; send the gene set for analysis with external tools; and produce reports that contain the results of the analysis and visualizations by selecting from a collection of methods and tools. Additionally, GeneSetCart enables the storage and sharing of gene sets in user accounts. GeneSetCart also has a Chrome extension. The extension only works on Gene Expression Omnibus (GEO) [16], PubMed, and PubMed Central (PMC) web pages. It uses the Rummagene [17] and RummaGEO [18] resources to assist users with extracting gene sets from GEO studies and PubMed articles and load them for analysis by GeneSetCart.

Data Description

Assembling gene set from different sources

Users of GetSetCart can assemble gene sets from multiple sources (Fig. 1A). The first source is user submitted gene sets. Users can upload gene sets using a few ways. Users can upload a .txt file containing a single gene set, or a .gmt file containing multiple gene sets. Gene sets can be created using a PubMed search. This feature uses the Geneshot [19] API to convert PMIDs into genes based on co-mentions in publications. Gene-publication associations are sourced from GeneRIF. Another source for assembling gene sets is from Enrichr [4]. Enrichr has over 500,000 annotated gene sets organized into >530 gene set libraries. The Enrichr gene set search functionality in GeneSetCart enables users to query Enrichr for gene set description given any search term. Once matching gene sets are found, they can be added to the GeneSetCart shopping cart. Similarly, GeneSetCart has a collection of gene sets created from NIH Common Fund programs. These gene sets can be queried and added to the cart in a similar manner as the way Enrichr gene sets are fetched. The final method to add gene sets into the GeneSetCart is via a Chrome extension. The GeneSetCart Chrome extension available from the Google Chrome Store, was developed with JavaScript and HTML. The extension enables users to add gene sets found in the Rummagene [17] and RummaGEO [18] databases when users visit the PubMed and GEO NCBI websites. The Rummagene database holds gene sets extracted from supplementary materials of publications deposited into PMC, while the RummaGEO gene sets are extracted from differential expression signatures automatically computed from the uniformly aligned RNA-seq GEO studies available from ARCHS4 [20]. For a given set, users can choose to only include valid human Entrez gene symbols from the NCBI Gene database or include any identifiers. This flexibility makes GeneSetCart applicable to handle other set types such as drug, variant, or metabolite sets.

Methods

Gene set augmentation

After assembling gene sets from various sources, users can expand their sets by adding similar genes to each set using the Augmentation step (Fig. 1B). The gene set augmentation feature takes a gene set from the shopping cart and based on the selected option for augmentation: co-expression, literature co-mentions, and protein-protein interactions (PPIs) the gene set is expanded with additional relevant genes. The co-expression option uses the Geneshot [19] API which returns genes that are co-expressed with the genes in the original set. The co-expressed genes are determined based on gene-gene co-expression correlations calculated from the processed data in ARCHS4 [20]. The literature co-mentions option also calls the Geneshot [19] API, but with the GeneRIF gene-gene similarity matrix parameter selected. The related genes are those mostly co-mentioned with the input gene set based on GeneRIF co-mentions in publications. The PPI option uses the Genes2Networks (G2N) [21] API (https://maayanlab.cloud/G2N/#api). This API endpoint returns genes that directly interact with genes in the sets based on known protein-protein interactions (PPI). The PPI in Genes2Networks are assembled from BioGRID [22], BioPlex [23], IntAct [24], MINT [25], PPID [26], iRefWeb [27], Stelzl et al. [28] and few other PPI resources. To construct PPI subnetworks, Genes2Networks [21] is utilizing the PPI from these databases, and the shortest path algorithm with a maximum path length of 2 between two seed genes. For each of the augmentation options, users can specify the maximum number of genes to be added by the augmentation with the default set to 200 genes. There is also an option to decide whether to include the original genes from the set, or only include the augmented genes.

Combining gene sets

GeneSetCart has an interface that facilitates users to select sets and combine them to generate additional sets using one of four set operations options: union, intersection, consensus, and subtract (Fig. 1B). The union operation returns a single gene set containing all elements that are in each of the selected gene sets. The intersection option returns a single set composed of all elements that belong to all the selected gene sets. The consensus option returns a single gene set composed of genes that appear in at least N of the selected sets, where N is a number specified by the user. The subtract option subtracts the genes from the first selected set from the union of all other selected sets.

Visualization of the overlap among selected gene sets

GeneSetCart can visualize the overlap between selected gene sets in the cart with several interactive and publication-ready plots including: Venn, Supervenn, UpSet, Hierarchically-Clustered Heatmaps, and Uniform Manifold Projection (UMAP) [29] (Fig. 1B). The Venn diagrams support the visualization of up to five sets and use the Reaviz React library [30]. This library renders React elements using the Data-Driven Documents (D3) JavaScript library [31]. For visualization of the overlap of more sets, UpSet plots are created by rendering React Javascript

XML (JSX) elements with D3 using the D3-UpSet library (https://github.com/chuntul/d3-upset). The SuperVenn plots are created with the React-SuperVenn library [32] which is an interactive React implementation of the Python supervenn library [33]. For the hierarchically clustered heatmap, we calculate the Jaccard similarity between all gene sets, and then use the Seaborn clustermap with default parameters to create the heatmap. To create the UMAP plots, we compute the document-term frequency matrix of all gene sets using the Term Frequency Inverse Document Frequency (TF-IDF) vectorizer function from the Scikit-learn Python package [34]. The Scanpy Python package [35] is used to create the Uniform Manifold Projection (UMAP) embeddings of the TF-IDF values which is visualized as a scatterplot created using React JSX and D3. In the UMAP, each point represents a gene set which can be colored based on two options. For the default option, we apply the Leiden algorithm [36] to the TF-IDF vectors, and the gene sets points are then colored based on their assigned cluster. For the user-assigned option, the application enables users to assign gene sets to groups by uploading a .csv file mapping each gene set to its desired group. The default UMAP parameters used are minDist=0.1, spread=1, nNeighbors=15, randomState=42. Sliders are provided for users to change these parameters. The Venn, Supervenn, UpSet, and UMAP plots are interactive. Users can view the number of genes in each gene set subsection in the visualization by clicking the displayed plot. The selected genes can be added to the cart for further downstream analysis. The generated visualizations are also available for download as publication-ready Portable Network Graphics (PNG) and Scalable Vector Graphics (SVG) images. The URL of the visualization can also be shared to view the plots on the GeneSetCart site.

Gene set analysis with external tools

Users of GeneSetCart submit the gene sets in their cart for analysis with external tools. There are currently nine such tools to choose from: Enrichr [4], Enrichr-KG [37], Rummagene [17], RummaGEO [18], ChIP-X Enrichment Analysis 3 (ChEA3) [38], Kinase Enrichment Analysis 3 (KEA3) [39], SigCOM LINCS [40], Common Fund Data Ecosystem Gene Set Enrichment (CFDE-GSE), and Playbook Workflow Builder (PWB) [41] (Fig. 1B). ChEA3 performs transcription factor (TFs) enrichment analysis to rank TFs associated with a given gene set. Similarly, KEA3 performs kinase enrichment analysis to find upstream kinases whose putative substrates are over-represented in an input gene set. SigCom LINCS performs signature similarity search for mimicker and reverser compounds by querying the gene set against a collection of one million gene expression signatures collected by the L1000 assay for the LINCS program [42]. The PWB uses the input gene set as an entry point for creating interactive workflows. A selected gene set in GeneSetCart is sent to one of the external tools using the tools APIs. The tool returns a persistent URL link to the given analysis of the gene set with the selected tool. This URL is used to visualize the enrichment analysis results in the browser.

Generating hypotheses with GPT

For each significant gene set crossing pair, the user can add the overlapping genes to the GeneSetCart shopping cart, send the overlapping genes to Enrichr [4] for enrichment analysis, and generate a hypothesis that provides a possible explanation for the highly significant overlap

between the gene sets pair. Such hypotheses are formed based on a textual description of each gene set and significantly enriched terms collected from Enrichr. To create a textual description of each gene set, we designed templates for each gene set library. The template has the experimental and computational procedures used to create each gene set from each Common Fund program. We prompt the GPT-4o model from OpenAI to parse the term associated with each gene set and place the appropriate parts of the term in the place fillers found in the templates. In order to provide the model more context to generate meaningful hypotheses, we perform gene set enrichment with the overlapping genes using the GO Biological Processes [43], WikiPathways [44], MGI Mammalian Phenotype [45], and GWAS Catalog [46] Enrichr libraries. The top five enriched terms from each of the libraries are added to the model prompt. The final prompt instructs the model to generate a hypothesis describing the reason for the high overlap between the two gene sets based on the two sets, the templates, and the enriched terms.

Generating reports with GeneSetCart

GeneSetCart can produce downloadable reports in HTML and PDF formats. These reports contain the visualization and analysis modules available from the site applied to a selected set of gene sets from the user's session. Users can select the gene sets, analysis tools, and visualization modules to include in the reports. Once executed, the reports are displayed in the browser in HTML format. The reports also have a button to download the report as a PDF file. The executed reports also have a link to the GeneSetCart session, a listing of the included gene sets and their lengths, and the selected visualization and analysis modules, as well as the figure and table legends. Overall, the reports feature of GeneSetCart enables users to easily export and share the most relevant results from their analysis.

The GeneSetCart web-based interface implementation

The GeneSetCart web application is implemented in Typescript with the NextJS version 14 framework. A PostgreSQL database is used to store all user, gene set, and crossing data, while a Prisma ORM is used to query the database. User authentication is done with Nextauth.js allowing users to log into the site using their KeyCloak authentication hosted by the CFDE Workbench web portal.

Analyses

The GeneSetCart user interface

The user interface of GeneSetCart starts with a homepage where users can begin a session by clicking on the "Start Here" button. This initiates a session, and the user is navigated to the Assemble page. In the top right corner of the site, users can log into their user account. Sessions created while a user is logged are automatically saved to that user's account such that they can be reinstated. All sessions initiated when a user is not logged are public sessions that can still be shared via a persistent URL. Sessions created by a logged in user, however, can be set to either be private or public. Thus, the site provides means for users to communicate the results of their

analyses to others. The platform also provides a secure store for gene sets that may come from an investigator's unpublished experimental results.

Crossing CFDE gene set libraries

The gene set library crossing feature in GeneSetCart provides access to tables that rank pairs of gene sets created from data collected by eight Common Fund programs. Currently, there are 10 gene set libraries in GeneSetCart created from the eight programs – the Library of Integrated Network-Based Cellular Signatures (LINCS) [42], Illuminating the Druggable Genome (IDG) [47], Metabolomics Workbench [48], the Knockout Mouse Phenotyping Program (KOMP2) [49], the Genotype-Tissue Expression (GTEx) [50], Glygen [51], Human BioMolecular Atlas Program (HuBMAP) [52], and the Molecular Transducers of Physical Activity Consortium (MotrPAC) [53] (Fig. 2A). To rank gene set pairs from different CF programs the significance of the overlap is computed with Fisher's exact test computed with the SciPy Python package [54]. The crossed gene set pairs with a p-value of <0.001 are retained. The gene sets from the different CFDE libraries are visualized with UMAP plots (Fig. 2B-2C). Some gene set libraries form a singular cluster such GTEx aging signatures (red), GTEx tissue expression profiles (green), IDG drug targets (purple) and KOMP2 (orange) while other libraries have multiple clusters such as the HubMAP BioMolecular Atlas Program Azimuth library (yellow). The LINCS libraries do not form clusters but instead show looping strings likely because of many gene sets with small overlap. The crossed gene sets of each library are first characterized by the percentage of significant crossing pairs (p<0.001). A lower triangle heatmap visualizes these percentages for each library pair (Fig. 2D-E). Unsurprisingly, we observed that libraries sourced from the same Common Fund program, for example GTEx aging signatures, and GTEx tissue expression, display the greatest overlap. Additionally, the gene set libraries sourced from the LINCS program display high overlap with the gene set libraries created from GTEx, GlyGen, and HuBMAP. This might be because most of these gene sets are created from transcriptomics. The gene set libraries created from KOMP2, and Metabolomics have the least overlap with other libraries. This might be because these gene sets were created from the data collected by methods that are unique to each program.

Case Study 1: Shared pathways implicated in aging and exercise by crossing gene set libraries created from GTEx and MoTrPAC

Aging is a risk factor for many common chronic diseases [55,56] such as type 2 diabetes [57], cardiovascular disease [58] and neurological disorders such as Alzheimer's [59] and Parkinson's disease [60]. Moderate exercise [61] is widely accepted as a mechanism to promote overall health and aid in the prevention of aging related diseases [62]. To investigate the common biological underpinnings that accompany both aging and exercise, and discover genes that are induced and repressed due to exercise and aging, we crossed the GTEx aging signatures with the MoTrPAC rat endurance training gene sets [63] (Fig. 3A, fig. S1). 346 gene set pairs have a significant overlap (p<0.001, Fisher's exact test). The top two gene set pairs (GTEx Blood 20-29 vs 60-69 Up ∩ T30-Blood-Rna Female 2W Down, and GTEx Blood 20-29 vs 70-79 Up ∩ T30-Blood-Rna Female 2W Down) have 35 (p-value=6.52e-38) and 26 (p-value=1.05e-24) overlapping genes,

respectively. The “GTEx Blood 20-29 vs 60-69 Up” gene set contains genes that are upregulated when comparing the blood of subjects aged 20-29 to those aged 60-69, and similarly the “GTEx Blood 20-29 vs 70-79 Up” gene set contains genes that are upregulated when comparing the blood of subjects aged 20-29 to those aged 70-79. The “T30-Blood-Rna Female 2W Down” gene set consists of genes that are downregulated in the blood of rats after two weeks of endurance training. Next, we added these two gene sets to GeneSetCart and used the intersection set operation to discover that these two sets share 24 genes in common (Fig. 3B).

Enrichment analysis applied to these 24 overlapping genes using Enrichr [4] found enriched pathways related to immune response, blood coagulation, and lipid metabolism, which are all processes known to be affected by both aging and physical activity (Fig. 3C-D). Some enriched terms are blood related processes that are particularly known to undergo significant changes with aging and exercise such as blood coagulation and fibrinolysis. It is well known that aging is associated with increased plasma levels of many proteins related to coagulation [64]. Additionally, acute bout of exercise is also associated with transient increase in blood coagulation, whereas moderate exercise is known to enhance blood fibrinolytic activity without activation of coagulation mechanisms, while heavy exercise induces simultaneous activation of blood fibrinolysis and coagulation [65]. Notably, coagulation and fibrinolysis genes are upregulated due to aging and down-regulated due to aerobic long-term exercise. Additionally, blood lipids are a likely source of human aging and exercise biomarkers with blood lipid levels including total cholesterol, low- and high-density lipoprotein cholesterol, and triglycerides changing in specific ways with age [66,67] while endurance exercise induces fat oxidation [68]. These results are in concordance with the notion that aerobic exercise can attenuate some of the hallmarks of aging [69]. The identified genes can become biomarkers and potential therapeutic targets for exercise mimickers. Some of the identified genes are already well-known targets and biomarkers, while others are completely unknown. A novelty assessment of the gene was performed by comparing the number of publications each gene has in GeneRIF (Fig. 3E). We found that 65% of the genes contained in those sets are associated with less than 100 publications listed on PubMed, with two genes (HAO1 and SLC25A47) having less than 10 publications. We also found that out of the 37 genes (union of both sets), only 11 are previously co-mentioned with the terms aging, exercise, or both. These genes are CRP, HNF4A, TTR, HAMP, FGB, RBP4, FGA, APOC1, HABP2, SERPINA4 and AGXT2 (Fig. 3F). These results suggest that many of the identified overlapping genes are understudied in the context of both aging and exercise, and as such they provide hypotheses that warrant further exploration.

Case Study 2: Exploring novel targets for Alexander Disease

Alexander disease (AxD) is a rare neurodegenerative disease caused by a mutation in the *GFAP* gene which codes for the glial fibrillary acidic protein (GFAP) [70]. The GFAP protein supports the formation of myelin sheaths in normal physiology, but in Alexander disease, the gain-of-function mutation in the *GFAP* gene causes the protein product to accumulate. Instead of helping maintain myelin sheaths, the extra GFAP causes damage to the myelin. The overexpression of GFAP in animal models also results in the appearance and accumulation of Rosenthal fibers (RF), protein aggregates in the cytoplasm of astrocytes [71], in subpial and white matter central nervous system

areas, which have typically high GFAP expression. Other than RF build-up, astrocytes in AxD also have abnormal cell shape and function. The Gene Expression Omnibus (GEO) is a major open biomedical research repository for transcriptomics and other omics datasets that currently contains millions of gene expression samples from tens of thousands of studies collected by research laboratories from around the world [72]. Here, we use the GeneSetCart pipeline to analyze gene sets created by comparing gene expression samples obtained from GEO of wild type (WT) or controls to AxD samples (Fig. 4A).

To obtain the AxD disease signatures, we perform differential gene expression analysis on RNA-seq gene expression samples from three GEO studies that compare control or wild type to AxD samples (GSE198817, GSE197044, GSE116327) [73]. GSE198817 contains gene expression samples from the hippocampus and corpus callosum tissue of Gfap+/+, Gfap+/R236H, and mGFAPTg170-2 transgenic mice. The GSE197044 study has RNA-seq profiles from hippocampus and corpus callosum tissue of male Gfap+/R236H and Gfap+/+ mice in FVB/N-Tac at 8 weeks of age; and the GSE116327 study has profiles from healthy controls and AxD patient's iPSC-derived astrocytes and post-mortem brain tissues. Differentially expressed genes between healthy controls and disease samples for each study are computed using the limma method [74]. This analysis was performed with the bulk RNA-seq analysis pipeline appyter [75]. The up and down genes were converted into gene sets. These gene sets were uploaded to GeneSetCart for further integrative analysis. Using the GeneSetCart Combine feature, consensus up and down sets were created. Choosing the consensus criteria of 3, the consensus up signature has 65 genes and the consensus down signature has 20 genes. These up and down consensus sets were submitted to SigCom LINCS [40] to identify potential drugs and preclinical small molecules that may reverse the disease gene expression changes in different cell lines. We also perform gene set enrichment analysis on the consensus up and down sets with Enrichr [4] (Fig 4B-D).

The consensus upregulated genes are enriched for transcription factors known to regulate immune response and inflammation. The top three transcription factors from the ChEA [38] analysis is RELA, IRF8 and STAT3 ($p<0.0001$, Fisher's exact test). Consistent with inflammation and AxD, the top enriched WikiPathways [44] pathway is Spinal Cord Injury WP2432 (p=8.234e-7) with the 6 overlapping genes: CXCL10, CCND1, CCL2, CXCL1, VIM, and GFAP. The most profound results from the enrichment analysis come from the MGI Mouse Phenotypes library with the top 4 most enriched terms: Increased Susceptibility to Induced Morbidity/Mortality MP:0009763 (p=1.576e-8), CNS Inflammation MP:0006082 (p=3.795e-7), Demyelination MP:0000921 (p=0.000004793), and Abnormal Myelination MP:0000920 (p=0.00001292). The knockout mice of the overlapping genes with these terms could serve as AxD disease models due to the shared phenotype. GFAP only overlaps with genes from the Abnormal Myelination MP:0000920 phenotype together with TYROBP, PTPRC, ADGRG6, and TLR2 (Fig. 4B). When querying Rummagene [17] with the consensus up genes, the brain inflammation signature is further confirmed. Several of the top matching sets in Rummagene are from brain inflammation studies with two studies about prion disease [76,77] suggesting potentially similar mechanisms between prion disease and AxD.

The consensus down-regulated genes are enriched for terms related to brain tissues and cell types. Specifically, markers for astrocytes are the top enriched terms from the gene set libraries created from CellMarker [78], Tabula Muris [79], PanglaoDB [80], and Allen Brain Atlas 10x scRNA [81] (Fig. 4C). This observation is also supported by a RummaGEO [18] query that returned matching gene sets from studies titled: RNA-Seq of human astrocytes GSE73721; Regionally specified human pluripotent stem cell-derived astrocytes GSE133489; and CROP-seq of hiPSC-derived astrocytes GSE182307 and GSE182309.

Discussion

GeneSetCart is a user-friendly platform designed to help biomedical researchers to explore knowledge about gene sets. The platform provides mechanisms to upload, compare, combine, visualize, save, share, manage, and analyze collections of gene sets. In addition, users of GeneSetCart can perform enrichment analyses using a variety of tools via the use of their APIs. Additional tools that accept gene sets could be added. The application builds on the functionality of many existing gene set analysis tools. For example, Geneshot [ref] is used to convert PubMed searches into gene sets, Genes2Networks [21] and ARCHS4 [20] are used to expand a gene set with PPI and gene-gene co-expression correlations, respectively. The gene set expansion functionality provides predictions about additional genes that may be involved in the same function as a given gene set. Such a gene set expansion approach can be used to form novel hypotheses and point to new targets. Machine Learning methods could be employed to improve this functionality in the future.

Potential implications

GeneSetCart was created to promote the reuse of NIH Common Fund datasets. So far, we have created 10 gene set libraries from 8 Common Fund programs. It is expected that additional libraries from more programs will be added to the system once such data becomes available. One of the use cases provided in this manuscript, and on the GeneSetCart site, crosses gene sets from two NIH Common Fund programs, namely GTEx [50] and MoTrPAC [53]. The use case shows that crossing the GTEx aging gene sets, created from profiling postmortem human tissues, with tissues from rats collected by MoTrPAC after prolonged aerobic exercise, produced interesting insights. However, this is just one example. Many other crossings and overlaps are made possible by GeneSetCart and remain to be explored. For example, exercise mimickers and aging reversers can be identified by crossing the gene set libraries created from the NIH Common Fund Library of Network-based Cellular Signatures (LINCS) [42] with those created from MoTrPAC and GTEx.

Availability of source code and requirements

Project name: GeneSetCart
Project home page: https://genesetcart.cfde.cloud/
Chrome extension:
https://chromewebstore.google.com/detail/genesetcart/dahaedghigbofibfadgedahlekhphmbd
GitHub repo: https://github.com/MaayanLab/GeneSetCart/
Operating system(s): Platform independent
Programming language: Python
License: CC BY-NC-SA 4.0
Any restrictions to use by non-academics: license needed.

Funding

This project was supported by NIH grants R01DK131525, OT2OD036435, OT2OD030160, U24CA264250, U24CA271114, and RC2DK131995.

Competing interests

The authors declare that they have no competing interests.

Figure Legends

Figure 1. GeneSetCart gene set input and analysis features. A. Users can upload a single gene set or enter a gene set in a text area; Upload a .gmt file containing multiple gene sets; Search PubMed for any term, then the returned PMIDs are converted into sene set based on GeneRIF or AutoRIF; Fetch gene sets created from data collected by Common Fund programs; Fetch gene sets from Enrichr gene set libraries related to an input term. **B.** The Augment feature allows augmentation of a gene set with co-expressed genes or PPI; The Combine features provides mechanisms to perform set operations on gene sets to create new sets; The Visualize page contain tools to visualize the overlap among sets including: Venn diagrams, UpSet plots, SuperVenn, heatmaps and UMAP visualization; The Analyze page permits users to submit their gene sets to a collection of external tools; and the Report page provides means to generate reports using a selected set of sets, and tools for performing the analysis.

Figure 2. CFDE gene set libraries included in GeneSetCart. A. Gene set sizes created from various NIH Common Fund programs. **B.** UMAP plot of IDF vectors of gene sets across Common Fund gene set libraries excluding LINCS. **C.** UMAP plot of IDF vectors of gene sets across all NIH Common Fund gene set libraries. **D.** Percentage of significantly overlapping crossing pairs across all possible combinations of gene set libraries.

Figure 3. Exploring shared pathways implicated in aging and exercise from blood. A. Workflow diagram depicting the analysis steps of cross the gene set libraries created from GTEx and MoTrPAC. **B.** Screenshot from the Venn diagram visualization created by GeneSetCart of the overlap between the top two crossing results of GTEx Aging Signatures vs MoTrPAC Rat Endurance Training libraries. **C.** Enrichment analysis of the 24 overlapping genes from the top two crossing with the KEGG 2021 Human gene set library in Enrichr. **D.** Enrichment analysis of the top 24 overlapping genes with the GO Biological Processes library in Enrichr. **E.** Publication count associated with all identified genes colored by set source. **F.** Publication for each gene colored by co-mentions with the terms aging, exercise, both, or neither.

Figure 4. Investigating mechanisms in Alexander's Disease. A. Use case workflow and analysis steps. **B.** Top 10 enriched terms from ChEA 2022 library of consensus up signature (n=3) gene set in Enrichr. **C.** Top 10 enriched terms from WikiPathway 2023 Mouse library of consensus up signature (n=3) gene set in Enrichr. **D.** Top 10 enriched terms from MGI Mammalian Phenotype Level 4 2024 library of consensus up signature gene set in Enrichr.

Figure S1. Hypothesis generation for crossing the MoTrPAC exercise signatures with the GTEx tissue aging signatures. A. The CFDE GMT Crossing page results of crossing the GTEx Tissue-Specific Aging Signatures with the MoTrPAC Rat Endurance Training libraries. **B.** Overlapping genes with the lowest p-values for gene set pair overlap. **C.** GPT-4 generated hypothesis of lowest p-value gene set pair.

References

1. Subramanian A, Tamayo P, Mootha VK, Mukherjee S, Ebert BL, Gillette MA, et al. Gene set enrichment analysis: a knowledge-based approach for interpreting genome-wide expression profiles. *Proc Natl Acad Sci U S A*. 102:15545–50 2005;

2. Ma'ayan A, Rouillard AD, Clark NR, Wang Z, Duan Q, Kou Y. Lean Big Data integration in systems biology and systems pharmacology. *Trends Pharmacol Sci*. Elsevier BV; 35:450–60 2014;

3. Rouillard AD, Gundersen GW, Fernandez NF, Wang Z, Monteiro CD, McDermott MG, et al. The harmonizome: a collection of processed datasets gathered to serve and mine knowledge about genes and proteins. *Database* 2016, baw100, 2016;

4. Chen EY, Tan CM, Kou Y, Duan Q, Wang Z, Meirelles GV, et al. Enrichr: interactive and collaborative HTML5 gene list enrichment analysis tool. *BMC Bioinformatics*. 14:128 2013;

5. Reimand J, Kull M, Peterson H, Hansen J, Vilo J. g:Profiler--a web-based toolset for functional profiling of gene lists from large-scale experiments. *Nucleic Acids Res*. 35:W193–200 2007;

6. Sherman BT, Hao M, Qiu J, Jiao X, Baseler MW, Lane HC, et al. DAVID: a web server for functional enrichment analysis and functional annotation of gene lists (2021 update). *Nucleic Acids Res*. 50:W216–21 2022;

7. Elizarraras JM, Liao Y, Shi Z, Zhu Q, Pico AR, Zhang B. WebGestalt 2024: faster gene set analysis and new support for metabolomics and multi-omics. *Nucleic Acids Res*. 52:W415–21 2024;

8. Chen J, Bardes EE, Aronow BJ, Jegga AG. ToppGene Suite for gene list enrichment analysis and candidate gene prioritization. *Nucleic Acids Res*. 37:W305–11 2009;

9. Zhou Y, Zhou B, Pache L, Chang M, Khodabakhshi AH, Tanaseichuk O, et al. Metascape provides a biologist-oriented resource for the analysis of systems-level datasets. *Nat Commun*. 10:1523 2019;

10. Karatzas E, Baltoumas FA, Aplakidou E, Kontou PI, Stathopoulos P, Stefanis L, et al. Flame (v2.0): advanced integration and interpretation of functional enrichment results from multiple sources. *Bioinformatics*. 39, 8, btad490 2023;

11. Schölz C, Lyon D, Refsgaard JC, Jensen LJ, Choudhary C, Weinert BT. Avoiding abundance bias in the functional annotation of post-translationally modified proteins. *Nat Methods*. 12:1003–4 2015;

12. Zhang B, Kirov S, Snoddy J. WebGestalt: an integrated system for exploring gene sets in various biological contexts. *Nucleic Acids Res*. 33:W741–8 2005;

13. Khan A, Mathelier A. Intervene: a tool for intersection and visualization of multiple gene or genomic region sets. *BMC Bioinformatics*. 18:287 2017;

14. Yang M, Chen T, Liu Y-X, Huang L. Visualizing set relationships: EVenn's comprehensive approach to Venn diagrams. *Imeta*. 3:e184 2024;

15. Shen L. GeneOverlap: An R package to test and visualize gene overlaps. *R Package*. bioconductor.statistik.tu-dortmund.de; 2016;

16. Clough E, Barrett T, Wilhite SE, Ledoux P, Evangelista C, Kim IF, et al. NCBI GEO: archive for gene expression and epigenomics data sets: 23-year update. *Nucleic Acids Res*. 52:D138–44 2024;

17. Clarke DJB, Marino GB, Deng EZ, Xie Z, Evangelista JE, Ma’ayan A. Rummagene: massive mining of gene sets from supporting materials of biomedical research publications. *Commun Biol*. 7:482 2024;

18. Marino GB, Clarke DJB, Lachmann A, Deng EZ, Ma’ayan A. RummaGEO: Automatic mining of human and mouse gene sets from GEO. *Patterns (N Y)*. 5:101072 2024;

19. Lachmann A, Schilder BM, Wojciechowicz ML, Torre D, Kuleshov MV, Keenan AB, et al. Geneshot: search engine for ranking genes from arbitrary text queries. *Nucleic Acids Res*. academic.oup.com; 47:W571–7 2019;

20. Lachmann A, Torre D, Keenan AB, Jagodnik KM, Lee HJ, Wang L, et al. Massive mining of publicly available RNA-seq data from human and mouse. *Nat Commun*. 9:1366 2018;

21. Berger SI, Posner JM, Ma’ayan A. Genes2Networks: connecting lists of gene symbols using mammalian protein interactions databases. *BMC Bioinformatics*. Springer; 8:372 2007;

22. Breitkreutz B-J, Stark C, Tyers M. The GRID: The General Repository for Interaction Datasets. *Genome Biol*. Genome Biol; 4:R23 2003;

23. Huttlin EL, Ting L, Bruckner RJ, Gebreab F, Gygi MP, Szpyt J, et al. The BioPlex network: A systematic exploration of the human interactome. *Cell*. Elsevier BV; 162:425–40 2015;

24. Hermjakob H, Montecchi-Palazzi L, Lewington C, Mudali S, Kerrien S, Orchard S, et al. IntAct: an open source molecular interaction database. *Nucleic Acids Res*. Oxford University Press (OUP); 32:D452–5 2004;

25. Zanzoni A, Montecchi-Palazzi L, Quondam M, Ausiello G, Helmer-Citterich M, Cesareni G. MINT: A molecular INTeraction database. *FEBS Lett*. Wiley; 513:135–40 2002;

26. Husi, H., Grant, S.G.N. (2003). Construction of a Protein-Protein Interaction Database (PPID) for Synaptic Biology. In: Kötter, R. (eds) Neuroscience Databases. Springer, Boston, MA.

27. Turner B, Razick S, Turinsky AL, Vlasblom J, Crowdy EK, Cho E, et al. iRefWeb: interactive analysis of consolidated protein interaction data and their supporting evidence. *Database (Oxford)*. Oxford Academic; 2010:baq023 2010;

28. Stelzl U, Worm U, Lalowski M, Haenig C, Brembeck FH, Goehler H, et al. A human protein-protein interaction network: a resource for annotating the proteome. *Cell*. Elsevier BV; 122:957–68 2005;

29. McInnes et al., UMAP: Uniform Manifold Approximation and Projection. Journal of Open Source Software, 3(29), 861 2018;

30. reaviz: Data visualization library for React. Maintained by @goodcodeus. Github;

31. Bostock M, Ogievetsky V, Heer J. D[3]: Data-Driven Documents. *IEEE Trans Vis Comput Graph*. ieeexplore.ieee.org; 17:2301–9 2011;

32. react_supervenn at main · MaayanLab/react-supervenn. Github;

33. Fedor. supervenn: supervenn: precise and easy-to-read multiple sets visualization in Python. Github;

34. Pedregosa F, Varoquaux G, Gramfort A, Michel V, Thirion B, Grisel O, et al. Scikit-learn: Machine Learning in Python. *J Mach Learn Res*. 12, 2825-30, 2011;

35. Wolf FA, Angerer P, Theis FJ. SCANPY: large-scale single-cell gene expression data analysis. *Genome Biol*. Springer; 19:15 2018;

36. Traag VA, Waltman L, van Eck NJ. From Louvain to Leiden: guaranteeing well-connected communities. *Sci Rep*. 9:5233 2019;

37. Evangelista JE, Xie Z, Marino GB, Nguyen N, Clarke DJB, Ma'ayan A. Enrichr-KG: bridging enrichment analysis across multiple libraries. *Nucleic Acids Res*. 51:W168–79 2023;

38. Keenan AB, Torre D, Lachmann A, Leong AK, Wojciechowicz ML, Utti V, et al. ChEA3: transcription factor enrichment analysis by orthogonal omics integration. *Nucleic Acids Res*. 47:W212–24 2019;

39. Kuleshov MV, Xie Z, London ABK, Yang J, Evangelista JE, Lachmann A, et al. KEA3: improved kinase enrichment analysis via data integration. *Nucleic Acids Res*. 49:W304–16 2021;

40. Evangelista JE, Clarke DJB, Xie Z, Lachmann A, Jeon M, Chen K, et al. SigCom LINCS: data and metadata search engine for a million gene expression signatures. *Nucleic Acids Res*. 50:W697–709 2022;

41. Clarke DJB, Evangelista JE, Xie Z, Marino GB, Maurya M, Srinivasan S, et al. Playbook Workflow Builder: Interactive construction of bioinformatics workflows from a network of microservices. *bioRxiv*. 2024; doi: 10.1101/2024.06.08.598037.

42. Keenan AB, Jenkins SL, Jagodnik KM, Koplev S, He E, Torre D, et al. The Library of Integrated Network-Based Cellular Signatures NIH Program: System-Level Cataloging of Human Cells Response to Perturbations. *Cell Syst*. 6:13–24 2018;

43. Gene Ontology Consortium. Gene Ontology Consortium: going forward. *Nucleic Acids Res*. 43:D1049–56 2015;

44. Kutmon M, Riutta A, Nunes N, Hanspers K, Willighagen EL, Bohler A, et al. WikiPathways: capturing the full diversity of pathway knowledge. *Nucleic Acids Res*. 44:D488–94 2016;

45. Blake JA, Bult CJ, Eppig JT, Kadin JA, Richardson JE, Mouse Genome Database Group. The Mouse Genome Database genotypes::phenotypes. *Nucleic Acids Res*. 37:D712–9 2009;

46. Sollis E, Mosaku A, Abid A, Buniello A, Cerezo M, Gil L, et al. The NHGRI-EBI GWAS Catalog: knowledgebase and deposition resource. *Nucleic Acids Res*. 51:D977–85 2023;

47. Oprea TI, Bologa CG, Brunak S, Campbell A, Gan GN, Gaulton A, et al. Unexplored

therapeutic opportunities in the human genome. *Nat Rev Drug Discov*. Nat Rev Drug Discov; 17:317–32 2018;

48. Sud M, Fahy E, Cotter D, Azam K, Vadivelu I, Burant C, et al. Metabolomics Workbench: An international repository for metabolomics data and metadata, metabolite standards, protocols, tutorials and training, and analysis tools. *Nucleic Acids Res*. Oxford University Press (OUP); 44:D463–70 2016;

49. Dickinson ME, Flenniken AM, Ji X, Teboul L, Wong MD, White JK, et al. High-throughput discovery of novel developmental phenotypes. *Nature*. Nature; 537:508–14 2016;

50. GTEx Consortium. The Genotype-Tissue Expression (GTEx) project. *Nat Genet*. 45:580–5 2013;

51. York WS, Mazumder R, Ranzinger R, Edwards N, Kahsay R, Aoki-Kinoshita KF, et al. GlyGen: Computational and informatics resources for glycoscience. *Glycobiology*. 30:72–3 2020;

52. HuBMAP Consortium. The human body at cellular resolution: the NIH Human Biomolecular Atlas Program. *Nature*. 574:187–92 2019;

53. Sanford JA, Nogiec CD, Lindholm ME, Adkins JN, Amar D, Dasari S, et al. Molecular Transducers of Physical Activity Consortium (MoTrPAC): Mapping the dynamic responses to exercise. *Cell*. 181:1464–74 2020;

54. Virtanen P, Gommers R, Oliphant TE, Haberland M, Reddy T, Cournapeau D, et al. SciPy 1.0: fundamental algorithms for scientific computing in Python. *Nat Methods*. 17:261–72 2020;

55. Wick G, Jansen-Dürr P, Berger P, Blasko I, Grubeck-Loebenstein B. Diseases of aging. *Vaccine*. 18:1567–83 2000;

56. Saul D, Kosinsky RL. Epigenetics of Aging and Aging-Associated Diseases. *Int J Mol Sci*. 22(1):401 2021;

57. Wilkerson HLC. Problems of an Aging Population. *Am J Public Health Nations Health*. American Public Health Association; 37:177–88 1947;

58. North BJ, Sinclair DA. The intersection between aging and cardiovascular disease. *Circ Res*. 110:1097–108 2012;

59. Xia X, Jiang Q, McDermott J, Han J-DJ. Aging and Alzheimer's disease: Comparison and associations from molecular to system level. *Aging Cell*. 17:e12802 2018;

60. Reeve A, Simcox E, Turnbull D. Ageing and Parkinson's disease: why is advancing age the biggest risk factor? *Ageing Res Rev*. 14:19–30 2014;

61. Caspersen CJ, Powell KE, Christenson GM. Physical activity, exercise, and physical fitness: definitions and distinctions for health-related research. *Public Health Rep*. 100:126–31 1985;

62. Fiuza-Luces C, Santos-Lozano A, Joyner M, Carrera-Bastos P, Picazo O, Zugaza JL, et al. Exercise benefits in cardiovascular disease: beyond attenuation of traditional risk factors. *Nat Rev Cardiol*. 15:731–43 2018;

63. Schenk S, Sagendorf TJ, Many GM, Lira A, DeSousa G, Bae D, et al. Physiological Adaptations to Progressive Endurance Exercise Training in Adult and Aged Rats: Insights from The Molecular Transducers of Physical Activity Consortium (MoTrPAC). *Function*. 5(4):zqae014 2024;

64. Tracy RP, Bovill EG. Thrombosis and cardiovascular risk in the elderly. *Arch Pathol Lab Med*. 116:1307–12 1992;

65. El-Sayed MS, Sale C, Jones PG, Chester M. Blood hemostasis in exercise and training. *Med Sci Sports Exerc*. 32:918–25 2000;

66. Johnson AA, Stolzing A. The role of lipid metabolism in aging, lifespan regulation, and age-related disease. *Aging Cell*. 18:e13048 2019;

67. Prospective Studies Collaboration, Lewington S, Whitlock G, Clarke R, Sherliker P, Emberson J, et al. Blood cholesterol and vascular mortality by age, sex, and blood pressure: a meta-analysis of individual data from 61 prospective studies with 55,000 vascular deaths. *Lancet*. 370:1829–39 2007;

68. Horowitz JF, Klein S. Lipid metabolism during endurance exercise. *Am J Clin Nutr*. 72:558S – 63S 2000;

69. Carapeto PV, Aguayo-Mazzucato C. Effects of exercise on cellular and tissue aging. *Aging* . 13:14522–43 2021;

70. Kuhn J, Cascella M. Alexander Disease. [Updated 2021 Jan 16]. In: StatPearls [Internet]. Treasure Island (FL): StatPearls Publishing; 2021;

71. Messing A, Head MW, Galles K, Galbreath EJ, Goldman JE, Brenner M. Fatal encephalopathy with astrocyte inclusions in GFAP transgenic mice. *Am J Pathol*. 152:391–8 1998;

72. Barrett T, Wilhite SE, Ledoux P, Evangelista C, Kim IF, Tomashevsky M, et al. NCBI GEO: archive for functional genomics data sets--update. *Nucleic Acids Res*. 41:D991–5 2013;

73. Gammie SC, Messing A, Hill MA, Kelm-Nelson CA, Hagemann TL. Large-scale gene expression changes in APP/PSEN1 and GFAP mutation models exhibit high congruence with Alzheimer’s disease. *PLoS One*. 19:e0291995 2024;

74. Ritchie ME, Phipson B, Wu D, Hu Y, Law CW, Shi W, et al. limma powers differential expression analyses for RNA-sequencing and microarray studies. *Nucleic Acids Res*. 43:e47 2015;

75. Clarke DJB, Jeon M, Stein DJ, Moiseyev N, Kropiwnicki E, Dai C, et al. Appyters: Turning Jupyter Notebooks into data-driven web apps. *Patterns (N Y)*. 2:100213 2021;

76. Slota JA, Medina SJ, Frost KL, Booth SA. Neurons and astrocytes elicit brain region specific transcriptional responses to prion disease in the Murine CA1 and thalamus. *Front Neurosci*. Frontiers Media SA; 16:918811 2022;

77. Crespo I, Roomp K, Jurkowski W, Kitano H, del Sol A. Gene regulatory network analysis supports inflammation as a key neurodegeneration process in prion disease. *BMC Syst Biol*. Springer Science and Business Media LLC; 6:132 2012;

78. Zhang X, Lan Y, Xu J, Quan F, Zhao E, Deng C, et al. CellMarker: a manually curated resource of cell markers in human and mouse. *Nucleic Acids Res*. 47:D721–8 2019;

79. Tabula Muris Consortium. A single-cell transcriptomic atlas characterizes ageing tissues in the mouse. *Nature*. 583:590–5 2020;

80. Franzén O, Gan L-M, Björkegren JLM. PanglaoDB: a web server for exploration of mouse and human single-cell RNA sequencing data. *Database (Oxford)*. 2019, baz046, 2019;

81. Shen EH, Overly CC, Jones AR. The Allen Human Brain Atlas: comprehensive gene expression mapping of the human brain. *Trends Neurosci*. 35:711–4 2012;

Figure 1

Click here to access/download;Figure;fig1.pdf

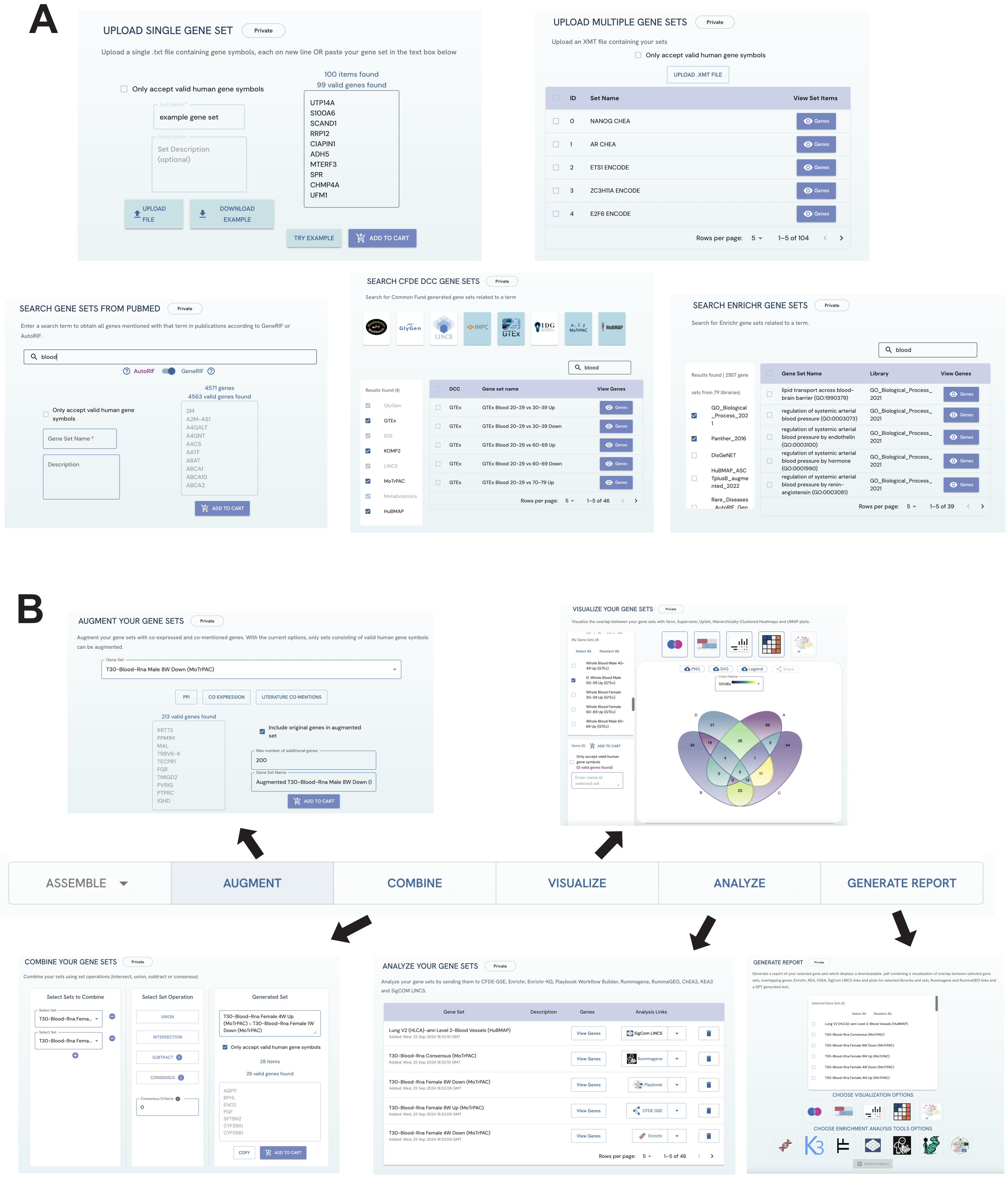

Figure 2

Click here to access/download;Figure;fig2.pdf

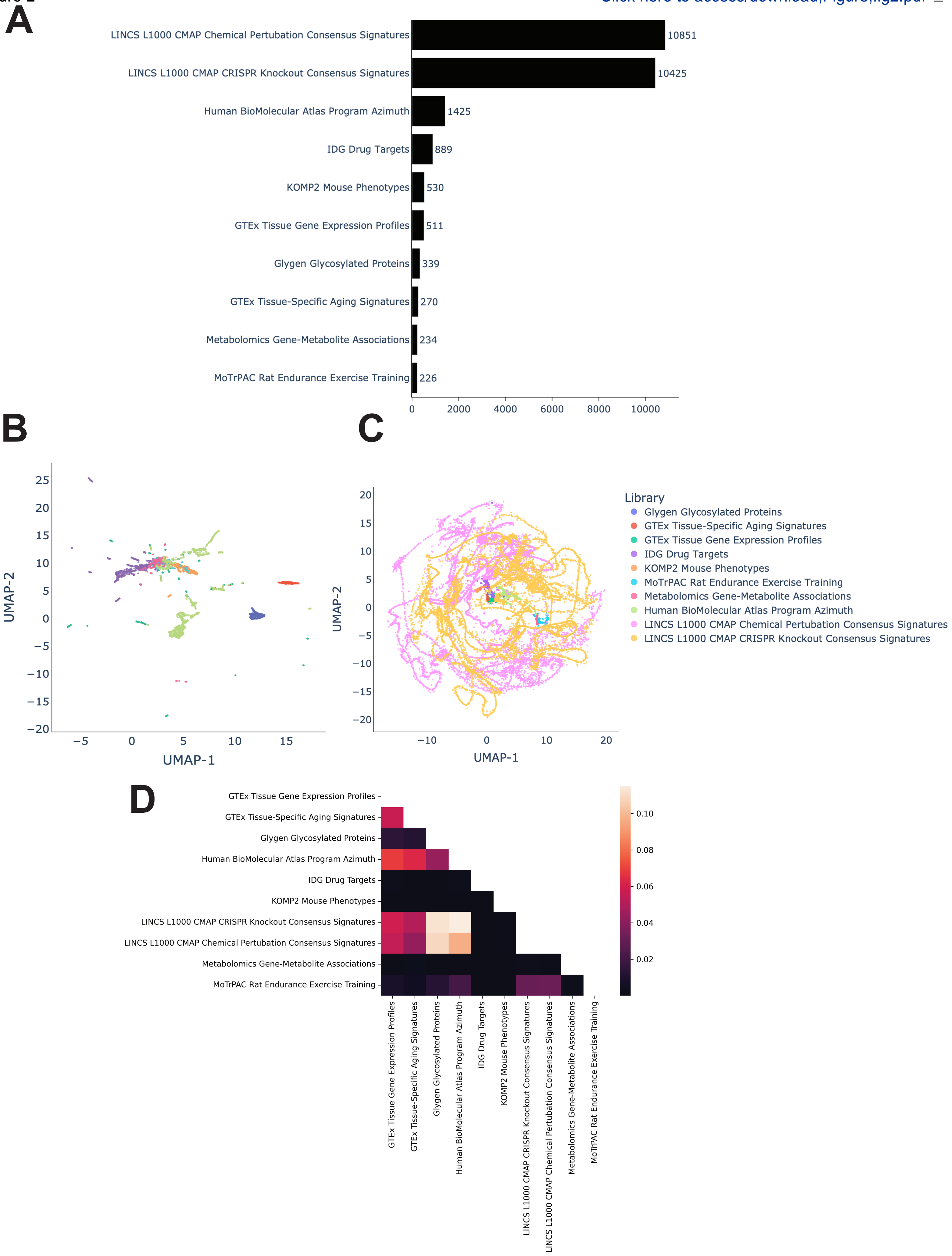

Figure 3

Click here to access/download;Figure;fig3.pdf

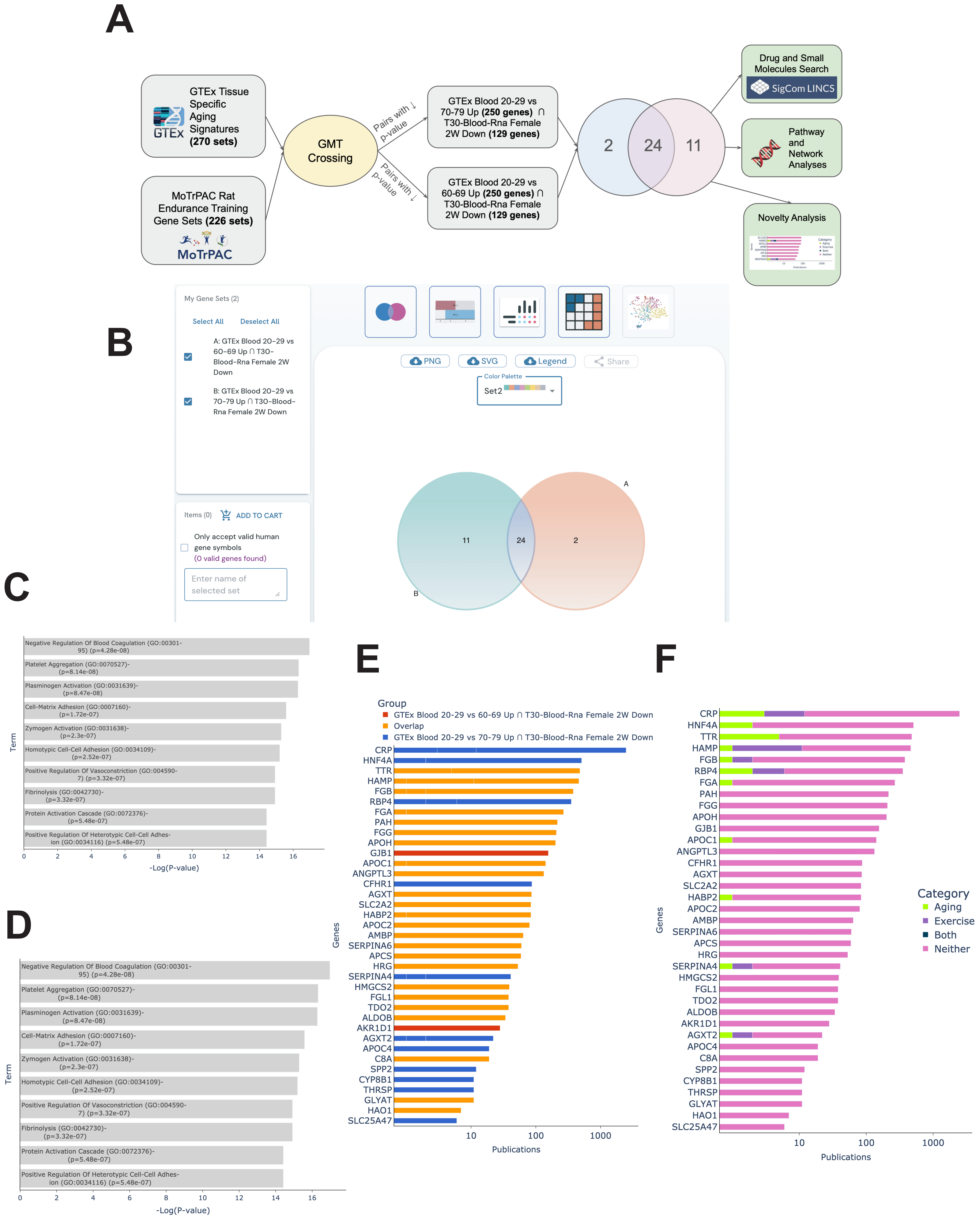

Figure 4

Click here to access/download;Figure;fig4.pdf

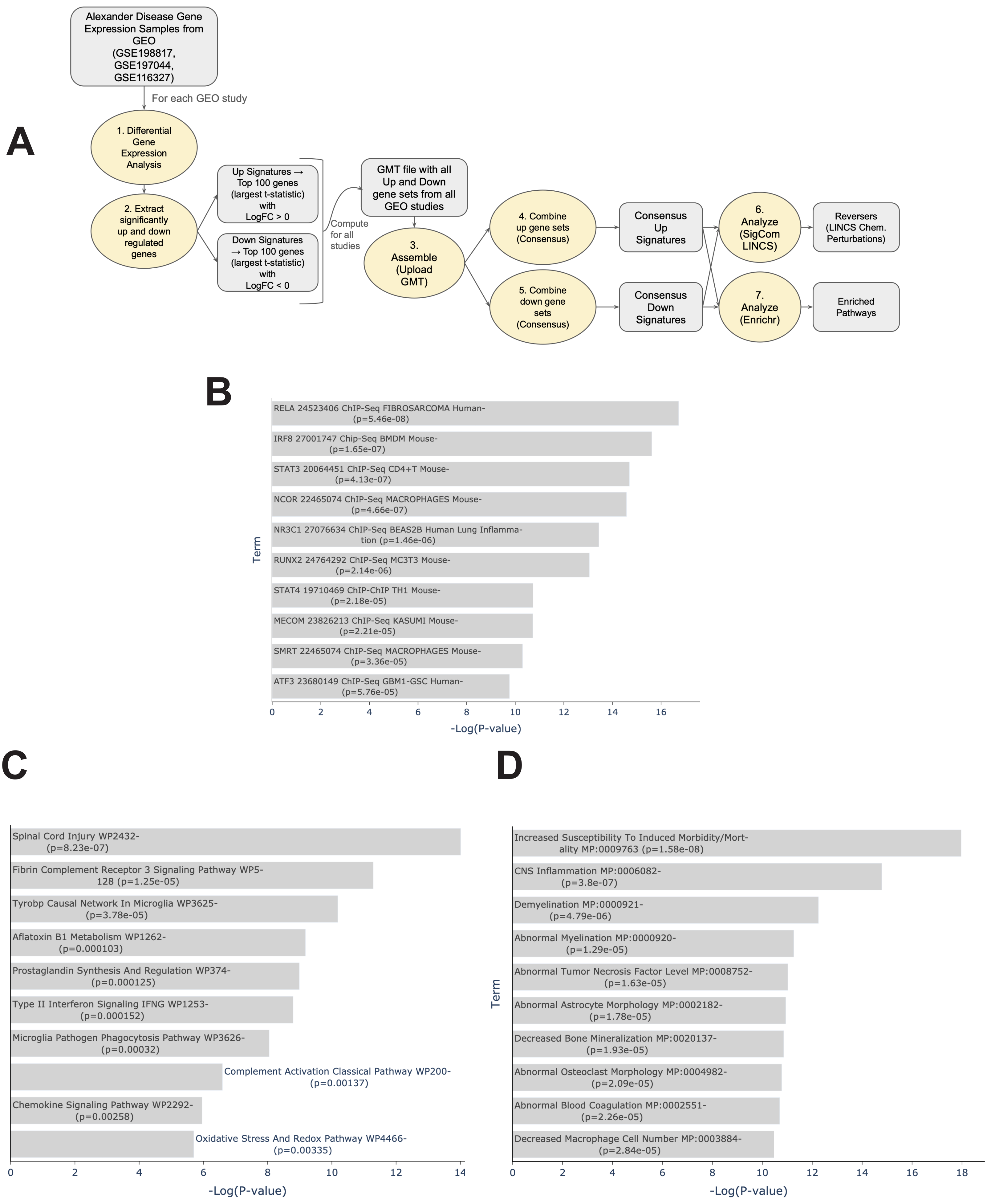

Figure S1 Click here to access/download;Figure;figS1.pdf

A

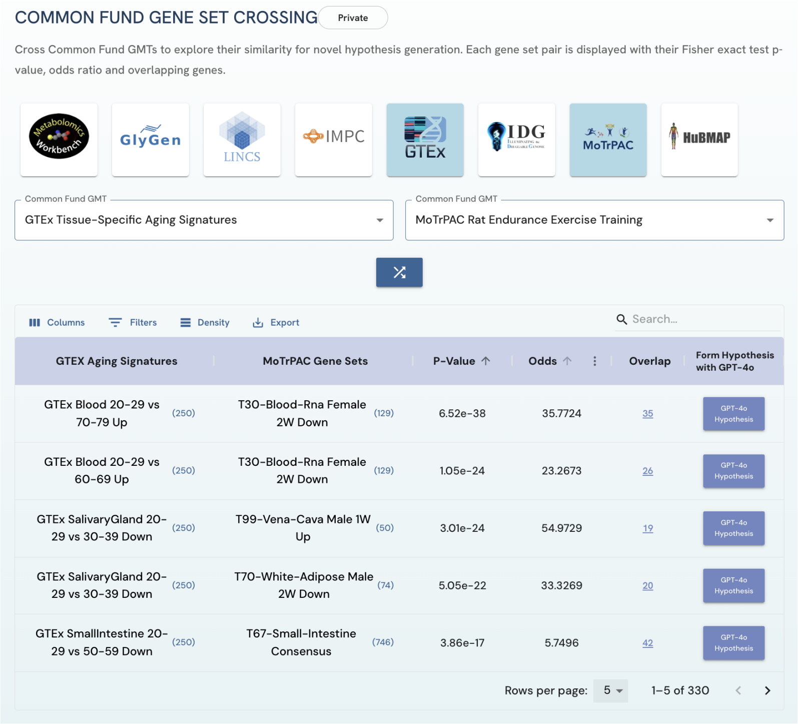

B

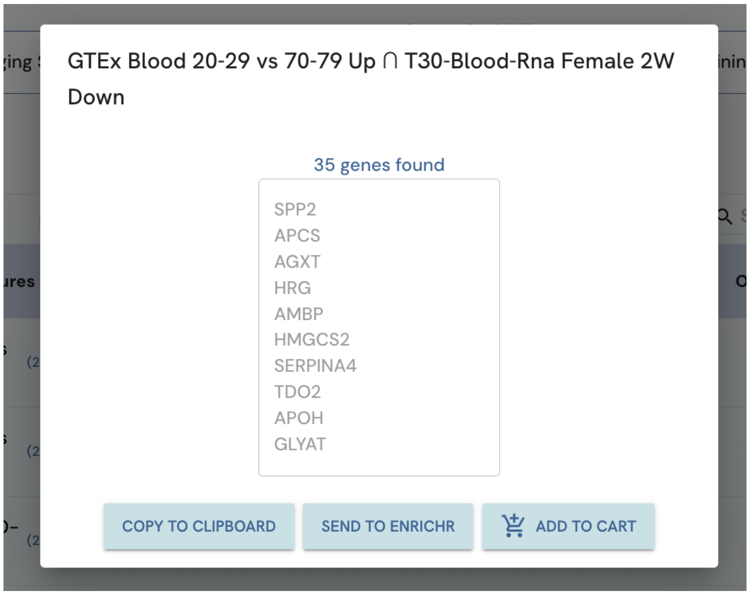

C

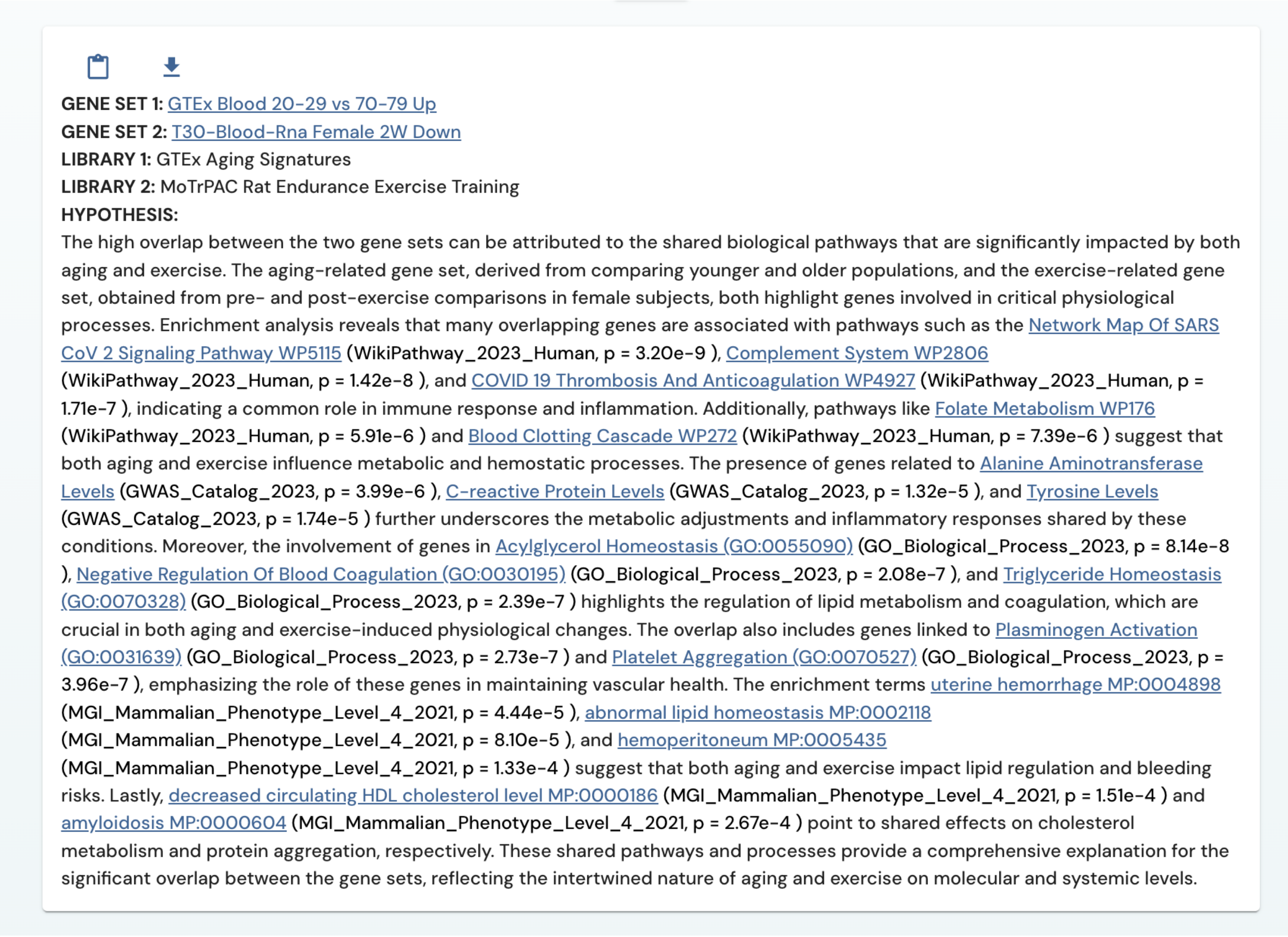

GENE SET 1: GTEx Blood 20-29 vs 70-79 Up
GENE SET 2: T30-Blood-Rna Female 2W Down
LIBRARY 1: GTEx Aging Signatures
LIBRARY 2: MoTrPAC Rat Endurance Exercise Training
HYPOTHESIS:
The high overlap between the two gene sets can be attributed to the shared biological pathways that are significantly impacted by both aging and exercise. The aging-related gene set, derived from comparing younger and older populations, and the exercise-related gene set, obtained from pre- and post-exercise comparisons in female subjects, both highlight genes involved in critical physiological processes. Enrichment analysis reveals that many overlapping genes are associated with pathways such as the Network Map Of SARS CoV 2 Signaling Pathway WP5115 (WikiPathway_2023_Human, p = 3.20e-9), Complement System WP2806 (WikiPathway_2023_Human, p = 1.42e-8), and COVID 19 Thrombosis And Anticoagulation WP4927 (WikiPathway_2023_Human, p = 1.71e-7), indicating a common role in immune response and inflammation. Additionally, pathways like Folate Metabolism WP176 (WikiPathway_2023_Human, p = 5.91e-6) and Blood Clotting Cascade WP272 (WikiPathway_2023_Human, p = 7.39e-6) suggest that both aging and exercise influence metabolic and hemostatic processes. The presence of genes related to Alanine Aminotransferase Levels (GWAS_Catalog_2023, p = 3.99e-6), C-reactive Protein Levels (GWAS_Catalog_2023, p = 1.32e-5), and Tyrosine Levels (GWAS_Catalog_2023, p = 1.74e-5) further underscores the metabolic adjustments and inflammatory responses shared by these conditions. Moreover, the involvement of genes in Acylglycerol Homeostasis (GO:0055090) (GO_Biological_Process_2023, p = 8.14e-8), Negative Regulation Of Blood Coagulation (GO:0030195) (GO_Biological_Process_2023, p = 2.08e-7), and Triglyceride Homeostasis (GO:0070328) (GO_Biological_Process_2023, p = 2.39e-7) highlights the regulation of lipid metabolism and coagulation, which are crucial in both aging and exercise-induced physiological changes. The overlap also includes genes linked to Plasminogen Activation (GO:0031639) (GO_Biological_Process_2023, p = 2.73e-7) and Platelet Aggregation (GO:0070527) (GO_Biological_Process_2023, p = 3.96e-7), emphasizing the role of these genes in maintaining vascular health. The enrichment terms uterine hemorrhage MP:0004898 (MGI_Mammalian_Phenotype_Level_4_2021, p = 4.44e-5), abnormal lipid homeostasis MP:0002118 (MGI_Mammalian_Phenotype_Level_4_2021, p = 8.10e-5), and hemoperitoneum MP:0005435 (MGI_Mammalian_Phenotype_Level_4_2021, p = 1.33e-4) suggest that both aging and exercise impact lipid regulation and bleeding risks. Lastly, decreased circulating HDL cholesterol level MP:0000186 (MGI_Mammalian_Phenotype_Level_4_2021, p = 1.51e-4) and amyloidosis MP:0000604 (MGI_Mammalian_Phenotype_Level_4_2021, p = 2.67e-4) point to shared effects on cholesterol metabolism and protein aggregation, respectively. These shared pathways and processes provide a comprehensive explanation for the significant overlap between the gene sets, reflecting the intertwined nature of aging and exercise on molecular and systemic levels.
