# Supplementary material for: GeneSetCart: assembling, augmenting, combining, visualizing, and analyzing gene sets
Source: Gigascience. 2025 Apr 10;14:giaf025. doi: 10.1093/gigascience/giaf025 (PMC11984350; doi:10.1093/gigascience/giaf025)

GigaScience

GeneSetCart: Assembling, Augmenting, Combining, Visualizing, and Analyzing Gene Sets

--Manuscript Draft--

**Manuscript Number:**	GIGA-D-24-00490R1
**Full Title:**	GeneSetCart: Assembling, Augmenting, Combining, Visualizing, and Analyzing Gene Sets
**Article Type:**	Research
**Funding Information:**	National Institute of Diabetes and Digestive and Kidney Diseases (R01DK131525) — Dr Avi Ma'ayan<br>National Institute of Diabetes and Digestive and Kidney Diseases (RC2DK131995) — Dr Avi Ma'ayan<br>NIH Office of the Director (OT2OD036435) — Dr Avi Ma'ayan<br>NIH Office of the Director (OT2OD030160) — Dr Avi Ma'ayan<br>Center for Biomedical Informatics and Information Technology, National Cancer Institute (U24CA264250) — Dr Avi Ma'ayan<br>Center for Biomedical Informatics and Information Technology, National Cancer Institute (U24CA271114) — Dr Avi Ma'ayan
**Abstract:**	Converting multi-omics datasets into gene sets facilitates data integration that leads to knowledge discovery. Although there are tools developed to analyze gene sets, only few offer the management of gene sets from multiple sources. GeneSetCart is an interactive web-based platform that enables investigators to gather gene sets from various sources; augment these sets with gene-gene co-expression correlations and protein-protein interactions; perform set operations on these sets such as union, consensus, and intersection; and visualize and analyze these gene sets, all in one place. GeneSetCart supports the upload of single or multiple gene sets, as well as fetching gene sets by searching PubMed for genes co-mentioned with terms in publications. Venn diagrams, heatmaps, UMAP, SuperVenn diagrams, and UpSet plots can visualize the gene sets in a GeneSetCart session to summarize the similarity and overlap among the sets. Users of GeneSetCart can also perform enrichment analysis on their assembled gene sets with external tools. All gene sets in a session can be saved to a user account for reanalysis and sharing with collaborators. GeneSetCart has a gene-set-library crossing feature that enables analysis of gene sets created from several NIH Common Fund programs. For the top overlapping sets from pairs of programs, a large language model (LLM) is prompted to propose possible reasons for the high overlap. Using this feature, two use cases are presented. In addition, users of GeneSetCart can produce publication-ready reports from their uploaded sets. Text in these reports is also assisted with an LLM. Overall, GeneSetCart is a useful resource for biologists without programming expertise to facilitate data integration for hypothesis generation.
**Corresponding Author:**	Avi Ma'ayan<br>Icahn School of Medicine at Mount Sinai<br>New York, NY UNITED STATES
**Corresponding Author Secondary Information:**	
**Corresponding Author's Institution:**	Icahn School of Medicine at Mount Sinai
**Corresponding Author's Secondary Institution:**	
**First Author:**	Giacomo B. Marino

Powered by Editorial Manager® and ProduXion Manager® from Aries Systems Corporation

**First Author Secondary Information:**	
**Order of Authors:**	Giacomo B. Marino
	Stephanie Olaiya
	John Erol Evangelista
	Daniel J. B. Clarke
	Avi Ma'ayan
**Order of Authors Secondary Information:**	
**Response to Reviewers:**	GeneSetCart - Replies to the Reviewers<br><br>Replies to Reviewer 1<br><br>Reviewer #1: GeneSetCart is a web-based platform for collection and analysis of gene sets from various sources. It will be useful for omics-focused researchers to understand their datasets. The introduction gives a good level of background information, especially around cloud based gene set collection and analysis tools with an emphasis on user friendly tools. It is made clear that GeneSetCart is a tool that is complementary to existing ones and builds on top of the team's existing portfolio including Enrichr, RummaGEO, ARCHS4, Harmonizome and other tools. The app looks like it was professionally designed, works smoothly and integrates well with other tools. Overall the manuscript is very well written, the app is thoroughly described and the case studies help to illuminate the potential of this type of analysis as well as identifying some interesting insights into aging and Alexander disease. I did find a few points in the manuscript that need further clarification.<br><br>Reply: Thank you for your positive and supportive comments about the manuscript and software application.<br><br>Reviewer #1: Which Species are supported? Is it possible to convert gene names based on ortholog relationships to facilitate cross-species comparisons? While the focus is on human and mouse, I can foresee this resource being extensively used by plant, animal and microbial researchers.<br><br>Reply: Thank you for this suggestion. The GeneSetCart platform mainly facilitates the analysis of human gene symbols, though it can accept any generic symbols or identifiers. To better accommodate additional species, we have added the ability to validate and standardize gene symbols for 18 species within the platform. Furthermore, each set is associated with an orthologous set of human gene symbols which are used for the downstream analysis tools because these tools mainly only accept human gene symbols.<br><br>Reviewer #1: Regarding gene identifiers - accession numbers are unique, but symbols/names are descriptive. Names also change over time. How is this managed? Will outdated gene symbols be updated over time? Will the tool be able to recognise Excel gene-date autocorrect errors? Can the tool ingest accessions from different sources like Ensembl and Entrez?<br><br>Reply: Thank you for this comment. We will utilize and keep up-to-date with the latest official NCBI gene symbols. Genes are stored in the database by their NCBI gene ID which are unique for each gene and can be associated with any changes to gene symbols over time. For the revised manuscript, we now added the functionality to recognize and correct Excel gene-date errors and other mismatching gene names. The NCBI gene information ingested into the GeneSetCart database also includes gene synonyms from Ensembl, Entrez, HGNC, OMIM, and previous symbols used by NCBI. These synonyms are utilized to map user-inputted genes into their standardized NCBI gene symbols supported by GeneSetCart.<br><br>Reviewer #1: As users will be uploading and working with gene sets, which could represent some important IP, the privacy and IP considerations might need some further clarity. Will gene sets and data remain private? What assurances are there that the data will remain private? Who retains IP ownership of the uploaded gene

signatures and the downstream works? What about the data sent to ChatGPT/OpenAI? Such details could be described in a terms of service document, which the user can retain for their records.

Reply: Thank you for this comment. Overall, sessions created by logged-in users who may upload their own gene sets are private by default. We have no intention of sharing gene sets without user consent. Users retain the IP ownership of their uploaded gene sets and their analysis using our tools. We can't make such assurances about gene sets and other text the user indirectly sends to OpenAI. For the revised manuscript, we added text to the manuscript about our policy. We also added a Terms of Service section to the GeneSetCart About page to cover these concerns.

Reviewer #1: Fig 2A needs an x-axis label.

Reply: Thank you for pointing this out. The x-axis label has been added.

Reviewer #1: Gene sets are truly useful in distilling large genomic datasets into a manageable size, but it should be emphasised that the Genes of Interest list (GoI) is influenced by the limitations of the technology and biological material (Timmons 2015; PMID: 26346307). Therefore when doing downstream enrichment analysis of these gene sets, we should be using a background gene list that eliminates those biases. One way to overcome this is by systematically collecting background gene lists for each GoI list, that is,the complete list of genes/proteins that were detected in the assay. Including an option for a background list for every GoI list would nudge the end user towards best practice. This point is relevant to the 24 gene enrichment shown in Fig 3B, where the background list correction could lead to half of the results being invalid (Wijesooriya 2022, PMID: 35263338, Fig 4C).

Reply: Thank you for this comment. We have added the ability to include a background gene set for each submitted gene set and utilize this background in any downstream tool that supports background. Since the overlap in Figure 3 reflects RNA-seq data, the background of all human protein-coding genes is appropriate and has been utilized.

Replies to Reviewer 2

Reviewer #2: In the submitted manuscript, the authors introduce GeneSetCart, a web-based platform designed to facilitate the management, augmentation, and analysis of gene sets from multiple sources, with a particular emphasis on those generated by NIH Common Fund programs. This platform has the potential to be highly useful for experimental biologists who often struggle with fetching and analyzing gene sets across diverse NIH web portals.

Reply: Thank you for your positive and supportive comments about the manuscript and software application.

Reviewer #2: The "Only Accept Valid Human Gene Symbols" option, if not selected at the beginning, can result in certain operations during the AUGMENT phase—such as selecting specific gene sets—failing. Highlighting this option prominently at the start would prevent invalid user operations and enhance usability.

Reply: Thank you for this comment. We have more prominently highlighted this option and added additional tools to convert gene symbols provided by the user into validated gene sets.

Reviewer #2: GeneSetCart offers three augmentation methods: PPI, Co-Expression, and Literature Co-Mentions. However, the PPI method seems to provide minimal augmentation compared to the other two. For example, applying PPI to the "GSEA: HALLMARK_IL2_STAT5_SIGNALING" human gene set added only three new genes, resulting in 196 genes, compared to 376 and 379 genes augmented by Co-Expression and Literature Co-Mentions, respectively.
- The authors should justify the inclusion of PPI as an augmentation method, especially given its relatively limited contribution.
- Alternatively, they could consider integrating enrichment tools specifically tailored for network data in the ANALYSIS phase, where high-quality protein-protein interaction

	data could play a more critical role.<br><br>Reply: Thank you for this comment. The default parameters to the PPI expansion augmentation method have been updated to provide a more comprehensive background of genes based on a larger integrated protein-protein interactions database. After implementing this upgrade, ~200 genes are added by all three augmentation methods.<br><br>Reviewer #2: GeneSetCart includes a wide range of analysis tools, such as CFDE-GSE, Enrichr, Enrichr-KG, Rummagene, RummaGEO, ChEA3, KEA3, SigCOM LINCS, L2S2, and PFOCRummage. However:<br>- Switching between these tools requires repeated manual navigation, which could be streamlined.<br>- Applying all tools generates a large volume of results, which might be overwhelming for experimental biologists to interpret.<br>Recommendation: Consider providing a batch-run option and integrating the results in an organized, user-friendly format to improve accessibility and usability.<br><br>Reply: Thank you for your suggestion. For the revised version of the application, we improved the way reports work. The updated reports not only provide a way to batch-run all the tools, but also display the results in a summary, and a way that enables the users to easily navigate to the sections of the report that are most relevant to them. We also made the reports persistent through a unique and persistent URL, added a table of contents, added a way to automatically generate an abstract, and we now also provide options for organizing the report by either analysis type or by gene set.<br><br>Reviewer #2: The platform's report generation time varies significantly depending on the size of the input gene set. Larger gene sets result in substantially longer generation times. Given the potential use of the platform with large-scale datasets, the authors should assess and report the platform's scalability.<br><br>Reply: Thank you for this comment and suggestion. For the revised version of the manuscript, we added a new figure (Fig. S1) that shows the average time it takes to analyze gene sets of various sizes with each of the reports' tools, as well as executing the report with all the tools. This information can assist users with understanding the wait times for their submitted gene sets.<br><br>Reviewer #2: The GPT-4o feature for generating explanatory hypotheses is currently restricted to the COMMON FUND GENE SET CROSSING module. However, users cannot apply this functionality to their own gene set intersections, limiting its broader applicability.<br>- The authors should clarify the rationale behind this restriction.<br>- Additionally, they should discuss whether this functionality can be extended to user-defined gene sets to enhance usability and versatility.<br><br>Reply: Thank you for this suggestion. For this revision, we added the ability for users to examine the overlap between their own gene sets and gene sets from all the CFDE-originated gene set libraries. This new feature was added to the gene-set-crossing interface. Additionally, for the Report section, we added the ability to form GPT-4o LLM hypotheses speculating about the overlap between two gene sets from their session. To activate this feature, the user needs to select two gene sets. To implement this feature, we utilize the gene set descriptions, the overlapping genes, and the Enrichr results for each set. With this information, we ask GPT-4o to generate a hypothesis.
**Additional Information:**	
**Question**	**Response**
Are you submitting this manuscript to a special series or article collection?	No
**Experimental design and statistics**	Yes

Powered by Editorial Manager® and ProduXion Manager® from Aries Systems Corporation

Full details of the experimental design and statistical methods used should be given in the Methods section, as detailed in our Minimum Standards Reporting Checklist. Information essential to interpreting the data presented should be made available in the figure legends.<br><br>Have you included all the information requested in your manuscript?	
**Resources**<br><br>A description of all resources used, including antibodies, cell lines, animals and software tools, with enough information to allow them to be uniquely identified, should be included in the Methods section. Authors are strongly encouraged to cite Research Resource Identifiers (RRIDs) for antibodies, model organisms and tools, where possible.<br><br>Have you included the information requested as detailed in our Minimum Standards Reporting Checklist?	Yes
**Availability of data and materials**<br><br>All datasets and code on which the conclusions of the paper rely must be either included in your submission or deposited in publicly available repositories (where available and ethically appropriate), referencing such data using a unique identifier in the references and in the "Availability of Data and Materials" section of your manuscript.<br><br>Have you have met the above requirement as detailed in our Minimum Standards Reporting Checklist?	Yes
GigaScience has policies and guidelines in place for the use of generative AI-writing tools such as ChatGPT. If you have used such writing tools to assist with	No

Powered by Editorial Manager® and ProduXion Manager® from Aries Systems Corporation

writing the manuscript this must be declared and cited in the text. Authors should not list AI-writing tools and other AI-assisted technologies as an author or co-author and should acknowledge that they are fully responsible for text generated or refined by AI-writing tools.<p><br><br>A summary of use (particularly in the introduction or among methods) needs to be included at the end of the paper, and the outputs should also be included as a supplementary file hosted in GigaDB or other open repositories. Please read our guidelines for more information. <p><br><br>By submitting to GigaScience, you are aware of the journal's AI-writing tools policy, and if you have declared use of such tools below, you have acknowledged this where appropriate in your manuscript and have made a summary of use and outputs available. <p><br><b>AI-assisted writing tools have been used in the preparation of this manuscript?	

Manuscript

Click here to access/download;Manuscript;GeneSetCart Manuscript 01252025.docx

GeneSetCart: Assembling, Augmenting, Combining, Visualizing, and Analyzing Gene Sets

Giacomo B. Marino[1], Stephanie Olaiya[1], John Erol Evangelista[1], Daniel J. B. Clarke[1], Avi Ma'ayan[1,*]

[1]Mount Sinai Center for Bioinformatics, Department of Pharmacological Sciences, Department of Artificial Intelligence and Human Health, Icahn School of Medicine at Mount Sinai, New York 10029, NY USA

*To whom correspondence should be addressed:
E-mail: avi.maayan@mssm.edu

Abstract

Converting multi-omics datasets into gene sets facilitates data integration that leads to knowledge discovery. Although there are tools developed to analyze gene sets, only few offer the management of gene sets from multiple sources. GeneSetCart is an interactive web-based platform that enables investigators to gather gene sets from various sources; augment these sets with gene-gene co-expression correlations and protein-protein interactions; perform set operations on these sets such as union, consensus, and intersection; and visualize and analyze these gene sets, all in one place. GeneSetCart supports the upload of single or multiple gene sets, as well as fetching gene sets by searching PubMed for genes co-mentioned with terms in publications. Venn diagrams, heatmaps, UMAP, SuperVenn diagrams, and UpSet plots can visualize the gene sets in a GeneSetCart session to summarize the similarity and overlap among the sets. Users of GeneSetCart can also perform enrichment analysis on their assembled gene sets with external tools. All gene sets in a session can be saved to a user account for reanalysis and sharing with collaborators. GeneSetCart has a gene-set-library crossing feature that enables analysis of gene sets created from several NIH Common Fund programs. For the top overlapping sets from pairs of programs, a large language model (LLM) is prompted to propose possible reasons for the high overlap. Using this feature, two use cases are presented. In addition, users of GeneSetCart can produce publication-ready reports from their uploaded sets. Text in these reports is also assisted with an LLM. Overall, GeneSetCart is a useful resource for biologists without programming expertise to facilitate data integration for hypothesis generation.

Keywords

Venn, UpSet, SuperVenn, gene set intersection, Geneshot, Integrative Analysis, Alexander Disease, Aging, Exercise, Chrome extension.

Background

The abstraction of biological and biomedical knowledge into gene sets has proven to be useful for data integration and reuse [1]. High dimensional omics datasets are commonly converted into gene set libraries which are collections of gene sets with annotations about the functions of each gene set in the library [2]. Gene sets can be created from many types of omics including genomics, proteomics, epigenomics, metabolomics, literature, and high-throughput drug and gene knockout, knockdown, and over-expression screening. Such gene sets can be differentially expressed genes from transcriptomics studies such as RNA-seq, targets of transcription factors from epigenomics experiments such as ChIP-seq, protein complexes from mass-spectrometry proteomics, genes that harbor mutations or deletions that lead to a human or a mouse phenotype from genomics studies, genes belonging to a pathway or a biological process based on literature curation, or marker genes that define specific cell types within a tissue [3].

There are several web-based platforms that facilitate the analysis of gene sets in the cloud. Most of these efforts focus on gene set enrichment analysis while others provide access to set operations and data visualizations. Enrichment analysis tools compute the significance of the overlap between an input gene set and background gene sets organized into gene set libraries. Enrichr is one example of a widely used gene set enrichment analysis tool that computes over-representation against a wide array of gene-set libraries created from a multitude of sources [4]. Another leading tool, gProfiler has the added functionality of ID conversion, multi-organism support, and support for single-nucleotide polymorphism (SNPs) enrichment analysis, but compared to Enrichr, gProfiler supports enrichment analysis with only a few selected gene set libraries [5]. One of the first, and still one of the leading platforms in this domain, is the Database for Annotation, Visualization, and Integrated Discovery (DAVID) [6].

DAVID also supports ID conversion, and additionally enables the combination of sets with the union operation. Other key widely-used enrichment analysis platforms are WebGestalt [7], GSEA [1], ToppGene [8], and Metascape [9]. However, most of these systems do not provide users with accounts where they can save and manage their gene sets and apply other types of analyses and visualizations on these sets. One example of a gene set management system is Flame [10]. Flame has the ability to upload multiple sets and perform combinatorial functional enrichment analysis for multiple organisms using different enrichment analysis tools that include aGOtool [11], gProfiler [5], WebGestalt [12], and Enrichr [4]. Flame also supports different gene identifiers, SNPs, and uploading free text that can be mined for genes and proteins using named entity recognition (NER) for a specific organism. However, Flame does not have the ability to save gene sets in a user account. Another application called Intervene [13] is a command line tool that visualizes intersection across gene sets with Venn diagrams, UpSet plots, and clustered heat maps. Intervene can generate five types of Venn diagrams: classical, Chow-Ruskey, Edwards, squares, and battleship. The web application Evenn [14] can be used to generate Venn diagrams including classical and Edwards, Euler proportional diagrams, UpSet plots, Flower plots, and Venn network diagrams. GeneOverlap [15] is an R package that can visualize gene set overlaps with heatmaps. Altogether, these resources are widely used by experimental biologists that study gene sets, but these applications lack many features and are not always user friendly.

GeneSetCart is a web-based application to manage the analysis of collections of gene sets. The platform provides access to some of the key functions implemented for the tools and services mentioned above, but also has some unique features that set it apart. Users of GeneSetCart can assemble gene sets from multiple sources including their own gene sets, annotated sets extracted from omics resources, and gene sets associated with biomedical terms from PubMed. Users can then augment these gene sets with related genes based on protein-protein interactions (PPI), co-expression, and co-mentions networks; visualize the overlap between their gene sets; send the gene set for analysis with external tools; and produce reports that contain the results of the analysis and visualizations by selecting from a collection of visualization methods and downstream analysis tools. Additionally, GeneSetCart enables the storage and sharing of gene sets in user accounts. GeneSetCart also has a Chrome extension. The extension only works when user visit the Gene Expression Omnibus (GEO) [16], PubMed, and PubMed Central (PMC) websites. The extension uses the Rummagene [17] and RummaGEO [18] resources to assist users with extracting gene sets from GEO studies and PubMed articles and load them for analysis by GeneSetCart.

Data Description

Assembling gene set from different sources

Users of GetSetCart can assemble gene sets from multiple sources (Fig. 1A). The first source is user submitted gene sets. Users can upload gene sets using a few ways. Users can upload a .txt file containing a single gene set, or a .gmt file containing multiple gene sets. Next, gene sets can be created using a PubMed search. This feature uses the Geneshot [19] API to convert PMIDs into genes based on co-mentions in publications. Gene-publication associations are sourced from GeneRIF. Another source for assembling gene sets is from Enrichr [4]. Enrichr has over 500,000 annotated gene sets organized into >530 gene set libraries. The Enrichr gene set search functionality in GeneSetCart enables users to query Enrichr's metadata for finding gene sets based on their description given any search term. Once matching gene sets are found, they can be added to the GeneSetCart shopping cart. Similarly, GeneSetCart has a collection of gene sets created from NIH Common Fund programs. These gene sets can be queried based on their description and added to the cart in a similar manner as the way Enrichr gene sets are fetched. The final method to add gene sets into the GeneSetCart is via a Chrome extension. The GeneSetCart Chrome extension available from the Google Chrome Store, was developed with JavaScript and HTML. The extension enables users to add gene sets found in the Rummagene [17] and RummaGEO [18] databases when users visit the PubMed, PMC, or the GEO NCBI websites. The Rummagene database holds gene sets extracted from supplementary materials of publications deposited into PMC, while the RummaGEO gene sets are extracted from differential expression signatures automatically computed from the uniformly aligned RNA-seq GEO studies available from the ARCHS4 resource [20]. For a given set, users can choose to only include valid human Entrez gene symbols from the NCBI Gene database or include any identifiers. This flexibility makes GeneSetCart applicable to handle other set types such as drugs, variants, and metabolites.

Methods

Gene names validation and mapping

All gene sets uploaded to GeneSetCart are converted into official NCBI gene symbols when the gene validation toggle is selected. Synonyms from other ontologies such as Ensembl and HGNC are supported and converted to official NCBI gene symbols. Users may select from 18 species to perform gene name conversion and validation. However, since most downstream tools support only human gene symbols, a copy of the set with valid mappable human gene symbols is also stored. Additionally, the gene conversion functionality includes correcting common Excel date conversion errors, where gene names are mistakenly converted to dates.

Gene set augmentation

After assembling gene sets from various sources, users can expand their sets by adding similar genes to each set using the Augmentation step (Fig. 1B). The gene set augmentation feature takes a gene set from the shopping cart and based on the selected option for augmentation: co-expression, literature co-mentions, and protein-protein interactions (PPIs), the gene set is expanded with additional relevant genes. The co-expression option uses the Geneshot [19] API which returns genes that are co-expressed with the genes in the original set. The co-expressed genes are determined based on gene-gene co-expression correlations calculated from the processed data in ARCHS4 [20]. The literature co-mentions option also calls the Geneshot [19] API, but with the GeneRIF gene-gene similarity matrix parameter selected. The related genes are those mostly co-mentioned with the input gene set based on GeneRIF co-mentions in publications. The PPI option uses the Genes2Networks (G2N) [21] API. This API endpoint returns genes that directly interact with genes in the sets based on known protein-protein interactions (PPI). The PPI in Genes2Networks are assembled from BioGRID [22], BioPlex [23], IntAct [24], MINT [25], PPID [26], iRefWeb [27], Stelzl et al. [28] and few other PPI resources. To construct PPI subnetworks, Genes2Networks [21] is utilizing the PPI from these databases, and the shortest path algorithm with a maximum path length of 2 between two seed genes. For each of these three augmentation options, users can specify the maximum number of genes to be added by the augmentation with the default set to 200 genes. There is also an option to decide whether to include the original genes from the set, or only include the augmented genes.

Combining gene sets

GeneSetCart has an interface that facilitates users to select sets and combine them to generate additional sets using one of four set operations options: union, intersection, consensus, and subtract (Fig. 1B). The union operation returns a single gene set containing all elements that are in each of the selected gene sets. The intersection option returns a single set composed of all elements that belong to all the selected gene sets. The consensus option returns a single gene set composed of genes that appear in at least N of the selected sets, where N is a number specified by the user. The subtract option subtracts the genes from the first selected set from the union of all other selected sets.

Visualization of the overlap among selected gene sets

GeneSetCart can visualize the overlap between selected gene sets in the cart with several interactive and statis publication-ready plots including: Venn diagrams, Supervenn diagrams, UpSet plots, hierarchically-clustered heatmaps, and Uniform Manifold Projection (UMAP) plots [29] (Fig. 1B). The Venn diagrams support the visualization of up to five sets and use the Reaviz React library [30]. This library renders React elements using the Data-Driven Documents (D3) JavaScript library [31]. For visualization of the overlap of more sets, UpSet plots are created by rendering React Javascript XML (JSX) elements with D3 using the D3-UpSet library (https://github.com/chuntul/d3-upset). The SuperVenn plots are created with the React-SuperVenn library [32] which is an interactive React implementation of the Python supervenn library [33]. For the hierarchically clustered heatmap, we calculate the Jaccard similarity between all gene sets, and then use the Seaborn clustermap function with default parameters to create the heatmap. To create the UMAP plots, we compute the document-term frequency matrix of all gene sets using the Term Frequency Inverse Document Frequency (TF-IDF) vectorizer function from the Scikit-learn Python package [34]. The Scanpy Python package [35] is then used to create the Uniform Manifold Projection (UMAP) embeddings of the TF-IDF values which are visualized as a scatterplot created using React JSX and D3. In the UMAP, each point represents a gene set. Points on the UMAP can be colored based on two options. The default coloring option applies the Leiden algorithm [36] to the TF-IDF vectors, and thus the gene sets points are colored based on their assigned cluster. For the user-assigned option, the application enables users to assign gene sets to groups by uploading a .csv file mapping each gene set to its desired group. The default UMAP parameters used are minDist=0.1, spread=1, nNeighbors=15, randomState=42. Sliders are provided for users to change these parameters. The Venn, Supervenn, UpSet, and UMAP plots are interactive. Users can view the number of genes in each gene set in the visualization by clicking the region in the plot that represent the gene set. The selected gene sets can be added to the cart for further downstream analysis. The generated visualizations are also available for download as publication-ready Portable Network Graphics (PNG) and Scalable Vector Graphics (SVG) images. The URL of the plots can be shared to view the plots again directly from the GeneSetCart website.

Gene set enrichment analysis with external tools

Users of GeneSetCart can submit the gene sets in their cart for analysis with external tools. There are currently 11 tools to choose from: Enrichr [4], Enrichr-KG [37], Rummagene [17], RummaGEO [18], ChIP-X Enrichment Analysis 3 (ChEA3) [38], Kinase Enrichment Analysis 3 (KEA3) [39], SigCom LINCS [40], LINCS L1000 Signature Search (L2S2), Common Fund Data Ecosystem Gene Set Enrichment (CFDE-GSE), and Playbook Workflow Builder (PWB) [41], and PFOCRummage (Fig. 1B). ChEA3 performs transcription factor (TFs) enrichment analysis to rank TFs associated with a given gene set. Similarly, KEA3 performs kinase enrichment analysis to find upstream kinases whose putative substrates are over-represented in an input gene set. SigCom LINCS and L2S2 perform signature similarity search for mimickers and reversers compounds and single gene knockouts by querying the gene set against a collection of one million

gene expression signatures collected by the L1000 assay for the LINCS program [42]. The PWB uses the input gene set as an entry point for creating interactive workflows. PFOCRummage facilitates the search of the gene set against the gene sets collected for the Pathway Figure Optical Character Recognition project [43,44]. A selected gene set in GeneSetCart is sent to one of these external tools using the tools' APIs. The tool returns a persistent URL link to the given analysis of the gene set with the selected tool. This URL is used to visualize the enrichment analysis results in the browser.

Generating hypotheses with a large language model (LLM)

For each significant gene set crossing pair, the user can add the overlapping genes to the GeneSetCart shopping cart, send the overlapping genes to Enrichr [4] for enrichment analysis, and generate a hypothesis that provides a possible explanation for the highly significant overlap between the gene sets pair. Such hypotheses are formed based on a textual description of each gene set and significantly enriched terms collected from Enrichr. To create a textual description of each gene set, we designed templates for each Common Fund Data Ecosystem (CFDE) gene set library. The template has the experimental and computational procedures used to create each gene set from each Common Fund program. We prompt the GPT-4o model from OpenAI to parse the terms associated with each gene set and place the appropriate parts of the term in the place fillers of the template. In order to provide the model more context to generate meaningful hypotheses, we perform gene set enrichment with the overlapping genes using the GO Biological Processes [45], WikiPathways [46], MGI Mammalian Phenotype [47], and the GWAS Catalog [48] Enrichr libraries. The top five enriched terms from each of these libraries are added to the model prompt. The final prompt instructs the model to generate a hypothesis describing the reason for the high overlap between the two gene sets based on the two sets, the templates, and the enriched terms. User uploaded sets can be compared to CFDE gene sets and to each other. When users select exactly two sets in the report mode, an option to form LLM-driven hypotheses about the possible relationship between the two set is provided.

Generating reports with GeneSetCart

GeneSetCart can produce downloadable persistently accessible reports provided in HTML and PDF formats. These reports contain the same visualization and analysis modules available from the visualize and analyze sections. These functions are applied to a selected small collection of gene sets from the user's session. Users can select up to five gene sets, analysis tools, and visualization modules to include in the report. Once executed, the reports are displayed in the browser in HTML format. The reports also have a button to download the report as a PDF file. Users can share reports with a provided persistent URL. The executed reports also have a link to the GeneSetCart session, a listing of the included gene sets and their size, a table of contents, and the selected visualization and analysis modules, as well as figure and table legends. Reports also contain text automatically generated by an LLM. If the user selects exactly two sets for the report, they can enable a hypothesis generation feature. To generate hypotheses, GeneSetCart utilizes the same elements as described for the CFDE gene set crossing feature. The time that it takes to generate reports depends on the number of gene sets and the tools the user selects, as

well as the size of the selected sets (fig. S1). Overall, the reports feature of GeneSetCart enable users to easily export and share the most relevant results from their gene set analyses.

The GeneSetCart web-based interface implementation

The GeneSetCart web application is implemented in Typescript with the NextJS version 14 framework. A PostgreSQL database is used to store all user, gene set, and crossing data, while a Prisma ORM is used to query the database. User authentication is applied with Nextauth.js enabling users to log into the site using their KeyCloak authentication hosted by the CFDE Workbench web portal (https://cfde.info).

Analyses

The GeneSetCart user interface

The user interface of GeneSetCart starts with a homepage where users can begin a session by clicking on the “Start Here” button. This initiates a session, and the user is navigated to the Assemble page. In the top right corner of the site, users can log into their user account. Sessions created while a user is logged are automatically saved to that user’s account such that they can be reinstated. All sessions initiated when a user is not logged are public sessions that can still be shared via a persistent URL. Sessions created by a logged in user, however, can be set to either be private or public. By default, all sessions are private. Users own the intellectual property of their sets, and these sets will not be share or published without the user’s consent. Users can share their sessions with their colleagues by making them public. Making sessions public is still hidden from most users because URLs to public sessions are also not shared with others by GeneSetCart and not indexed by search engines. Thus, the site provides means for users to communicate the results of their analyses to others while providing some level of privacy. While the platform provides a secure store for gene sets that may come from an investigator’s unpublished experimental results, investigators should be aware that we cannot guarantee 100% security of the GeneSetCart database. In addition, when gene sets are sent to third party applications, such as LLM services, there is a chance that such gene sets are save by these third-party applications.

Crossing CFDE gene set libraries

The gene set library crossing feature in GeneSetCart provides access to tables that rank pairs of gene sets created from data collected by eight NIH Common Fund (CF) supported programs. Currently, there are 10 gene set libraries in GeneSetCart created from these eight programs – the Library of Integrated Network-Based Cellular Signatures (LINCS) [42], Illuminating the Druggable Genome (IDG) [49], Metabolomics Workbench [50], the Knockout Mouse Phenotyping Program (KOMP2) [51], the Genotype-Tissue Expression (GTEx) [52], Glygen [53], Human BioMolecular Atlas Program (HuBMAP) [54], and the Molecular Transducers of Physical Activity Consortium (MotrPAC) [55] (Fig. 2A). To rank gene set pairs from different CF programs, the significance of the overlap between set pairs is computed with the Fisher's exact test computed

with the SciPy Python package [56]. The crossed gene set pairs with a p-value of <0.001 are retained. The gene sets from the different CF libraries are visualized with UMAP plots (Fig. 2B-2C). Some gene set libraries form a singular cluster such GTEx aging signatures (red), GTEx tissue expression profiles (green), IDG drug targets (purple) and KOMP2 (orange) while other libraries have multiple clusters such as the HubMAP Azimuth library (yellow). The LINCS libraries do not form clusters but instead show looping strings likely because of many gene sets with small overlap. The crossed gene sets of each library are first characterized by the percentage of significant crossing pairs (p<0.001). A lower triangle heatmap visualizes these percentages for each library pair (Fig. 2D-E). Unsurprisingly, we observed that libraries sourced from the same CF program, for example, GTEx aging signatures and GTEx tissue expression, display the greatest overlap (fig. S2). Additionally, the gene set libraries sourced from the LINCS program display high overlap with the gene set libraries created from GTEx, GlyGen, and HuBMAP. This might be because most of these gene sets are created from transcriptomics. The gene set libraries created from KOMP2 and Metabolomics have the least overlap with other libraries. This might be because these gene sets were created from data collected by methods that are unique to each program.

Case Study 1: Shared pathways implicated in aging and exercise by crossing gene set libraries created from GTEx and MoTrPAC

Aging is a risk factor for many common chronic diseases [57,58] such as type 2 diabetes [59], cardiovascular disease [60], and neurological disorders such as Alzheimer's [61] and Parkinson's diseases [62]. Moderate exercise [63] is widely accepted as a mechanism to promote overall health and aid in the prevention of aging related diseases [64]. To investigate the common biological underpinnings that accompany both aging and exercise, and discover genes that are induced or repressed due to exercise and aging, we crossed the GTEx aging signatures with the MoTrPAC rat endurance training gene sets [65] (Fig. 3A, fig. S3). 346 gene set pairs have a significant overlap (p<0.001, Fisher's exact test). The top two gene set pairs (GTEx Blood 20-29 vs 60-69 Up ∩ T30-Blood-Rna Female 2W Down, and GTEx Blood 20-29 vs 70-79 Up ∩ T30-Blood-Rna Female 2W Down) have 35 (p-value=6.52e-38) and 26 (p-value=1.05e-24) overlapping genes, respectively. The "GTEx Blood 20-29 vs 60-69 Up" gene set contains genes that are upregulated when comparing the blood of subjects aged 20-29 to those aged 60-69, and similarly the "GTEx Blood 20-29 vs 70-79 Up" gene set contains genes that are upregulated when comparing the blood of subjects aged 20-29 to those aged 70-79. The "T30-Blood-Rna Female 2W Down" gene set consists of genes that are downregulated in the blood of rats after two weeks of endurance training. Next, we added these two gene sets to GeneSetCart and used the intersection set operation to discover that these two sets share 24 genes in common (Fig. 3B).

Enrichment analysis applied to these 24 overlapping genes using Enrichr [4] found enriched pathways related to immune response, blood coagulation, and lipid metabolism, which are all processes known to be affected by both aging and physical activity (Fig. 3C-D). Some enriched terms are blood related processes that are particularly known to undergo significant changes with aging and exercise such as blood coagulation and fibrinolysis. It is well known that aging is associated with increased plasma levels of many proteins related to coagulation [66]. Additionally,

acute bout of exercise is also associated with transient increase in blood coagulation, whereas moderate exercise is known to enhance blood fibrinolytic activity without activation of coagulation mechanisms, while heavy exercise induces simultaneous activation of blood fibrinolysis and coagulation [67]. Notably, coagulation and fibrinolysis genes are upregulated due to aging in human blood and downregulated in rat blood due to aerobic long-term exercise. Additionally, blood lipids are a likely source of human aging and exercise biomarkers with blood lipid levels including total cholesterol, low- and high-density lipoprotein cholesterol, and triglycerides changing in specific ways with age [68,69] while endurance exercise induces fat oxidation [70]. These results are in concordance with the notion that aerobic exercise can attenuate some of the hallmarks of aging [71]. The identified genes can become biomarkers and potential therapeutic targets for exercise mimickers. Some of the identified genes are already well-known targets and biomarkers, while others are completely unknown. A novelty assessment of the gene was performed by comparing the number of publications each gene has on PubMed (Fig. 3E). We found that 65% of the genes contained in those sets are associated with less than 100 publications listed on PubMed, with two genes (HAO1 and SLC25A47) having less than 10 publications. We also found that out of the 37 genes (union of both sets), only 11 are previously co-mentioned with the terms aging, exercise, or both. These genes are CRP, HNF4A, TTR, HAMP, FGB, RBP4, FGA, APOC1, HABP2, SERPINA4 and AGXT2 (Fig. 3F). These results suggest that many of the identified overlapping genes are understudied in the context of both aging and exercise, and as such they provide hypotheses that warrant further exploration.

Case Study 2: Exploring novel targets for Alexander Disease

Alexander disease (AxD) is a rare neurodegenerative disease caused by a mutation in the *GFAP* gene which codes for the glial fibrillary acidic protein (GFAP) [72]. The GFAP protein supports the formation of myelin sheaths in normal physiology, but in AxD, the gain-of-function mutation in the *GFAP* gene causes the protein product to accumulate. Instead of helping maintain myelin sheaths, the extra GFAP causes damage to the myelin. The overexpression of GFAP in animal models also results in the appearance and accumulation of Rosenthal fibers (RF), protein aggregates in the cytoplasm of astrocytes [73], in subpial and white matter central nervous system areas, which have typically high *GFAP* expression. Other than RF build-up, astrocytes in AxD also have abnormal cell shape and function. The Gene Expression Omnibus (GEO) is a major open biomedical research repository for transcriptomics and other omics datasets that currently contains millions of gene expression samples from tens of thousands of studies collected by research laboratories from around the world [74]. Here, we use the GeneSetCart pipeline to analyze gene sets created by comparing gene expression samples obtained from GEO of wild type (WT) or controls to AxD samples (Fig. 4A).

To obtain the AxD disease signatures, we perform differential gene expression analysis on RNA-seq gene expression samples from three GEO studies that compare control or wild type cells and tissues to AxD samples (GSE198817, GSE197044, GSE116327) [75]. GSE198817 contains gene expression samples from the hippocampus and corpus callosum tissue of Gfap+/+, Gfap+/R236H, and mGFAPTg170-2 transgenic mice. The GSE197044 study has RNA-seq profiles from hippocampus and corpus callosum tissue of male Gfap+/R236H and Gfap+/+ mice

in FVB/N-Tac at 8 weeks of age; and the GSE116327 study has profiles from healthy controls and AxD patient's iPSC-derived astrocytes and post-mortem brain tissues. Differentially expressed genes between healthy controls and disease samples for each study are computed using the limma method [76]. This analysis was performed with the bulk RNA-seq analysis pipeline appyter [77]. The up and down genes were converted into gene sets. These gene sets were uploaded to GeneSetCart for further integrative analysis. Using the GeneSetCart Combine feature, consensus up and down sets were created. Choosing the consensus criteria of 3, the consensus up set has 65 genes and the consensus down set has 20 genes. These up and down consensus sets were submitted to SigCom LINCS [40] to identify potential drugs and preclinical small molecules that may reverse the disease gene expression changes in different cell lines. We also perform gene set enrichment analysis on the consensus up and down sets with Enrichr [4] (Fig 4B-D).

The consensus upregulated genes are enriched for transcription factors known to regulate immune response and inflammation. The top three transcription factors from the ChEA [38] analysis are RELA, IRF8 and STAT3 ($p<0.0001$, Fisher's exact test). Consistent with inflammation and AxD, the top enriched WikiPathways [46] pathway is Spinal Cord Injury WP2432 (p=8.234e-7) with the 6 overlapping genes: CXCL10, CCND1, CCL2, CXCL1, VIM, and GFAP. The most profound results from the enrichment analysis come from the MGI Mouse Phenotypes library with the top four most enriched terms: Increased Susceptibility to Induced Morbidity/Mortality MP:0009763 (p=1.576e-8), CNS Inflammation MP:0006082 (p=3.795e-7), Demyelination MP:0000921 (p=0.000004793), and Abnormal Myelination MP:0000920 (p=0.00001292). The knockout mice of the overlapping genes with these terms could serve as AxD disease models due to the shared phenotype. GFAP only overlaps with genes from the Abnormal Myelination MP:0000920 phenotype together with TYROBP, PTPRC, ADGRG6, and TLR2 (Fig. 4B). When querying Rummagene [17] with the consensus up genes, the brain inflammation signature is further confirmed. Several of the top matching sets in Rummagene are from brain inflammation studies with two studies about prion disease [78,79] suggesting potentially similar mechanisms between prion disease and AxD. The consensus down-regulated genes are enriched for terms related to brain tissues and cell types. Specifically, markers for astrocytes are the top enriched terms from the gene set libraries created from CellMarker [80], Tabula Muris [81], PanglaoDB [82], and Allen Brain Atlas 10x scRNA [83] (Fig. 4C). This observation is also supported by a RummaGEO [18] query that returned matching gene sets from studies titled: RNA-seq of human astrocytes GSE73721; Regionally specified human pluripotent stem cell-derived astrocytes GSE133489; and CROP-seq of hiPSC-derived astrocytes from GSE182307 and GSE182309. Altogether, the AxD down gene set appears to be related to astrocyte cell type specification, supporting the observed phenotype of defective astrocytes in AxD.

Discussion

GeneSetCart is a user-friendly platform designed to help biomedical researchers to explore knowledge about gene sets. The platform provides mechanisms to upload, compare, combine, visualize, save, share, manage, and analyze collections of gene sets. In addition, users of GeneSetCart can perform enrichment analyses using a variety of tools via the use of their APIs.

Additional tools that accept gene sets could be added. The application builds on the functionality of many existing gene set analysis tools. For example, Geneshot [19] is used to convert PubMed searches into gene sets, Genes2Networks [21] and ARCHS4 [20] are used to expand a gene set with PPI and gene-gene co-expression correlations, respectively. The gene set expansion functionality provides predictions about additional genes that may be involved in the same function as a given gene set. Such a gene set expansion approach can be used to form novel hypotheses and point to new targets. Machine Learning methods could be employed to improve this functionality in the future. GeneSetCart also employs LLMs to form hypotheses about the overlap between pairs of gene sets. Typically, such hypotheses state obvious general concepts that can be gleaned from examining the shared genes and enriched terms. However, LLM helps with articulating the most apparent patterns, and in some cases the LLM can provide surprising explanations. As LLMs improve, their ability to reason is expected to further improve this feature. User of GeneSetCart should also use caution when reading the LLM hypotheses because it is plausible for the LLM to make logical mistakes. One other limitation of GeneSetCart is that it is designed to handle a relatively small collection of gene sets. Users with hundreds of gene sets should seek other analysis with other tools, methods, and platforms.

Potential implications

GeneSetCart was created to promote the reuse of NIH Common Fund datasets. So far, we have created 10 gene set libraries from 8 Common Fund programs. It is expected that additional libraries from more programs will be added to the system once such data becomes available. One of the use cases provided in this manuscript, and on the GeneSetCart site, crosses gene sets from two NIH Common Fund programs, namely GTEx [52] and MoTrPAC [55]. The use case shows that crossing the GTEx aging gene sets, created from profiling postmortem human tissues, with tissues from rats collected by MoTrPAC after prolonged aerobic exercise, produced interesting insights. However, this is just one example. Many other crossings and overlaps are made possible by GeneSetCart and remain to be explored. For example, exercise mimickers and aging reversers can be identified by crossing the gene set libraries created from the NIH Common Fund Library of Network-based Cellular Signatures (LINCS) [42] with those created from MoTrPAC and GTEx.

Availability of source code and requirements

Project name: GeneSetCart
Project home page: https://genesetcart.cfde.cloud/
Chrome extension:
https://chromewebstore.google.com/detail/genesetcart/dahaedghigbofibfadgedahlekhphmbd
GitHub repo: https://github.com/MaayanLab/GeneSetCart/
Operating system(s): Platform independent
Programming language: Python
License: CC BY-NC-SA 4.0
Any restrictions to use by non-academics: license needed.
Bio.tools Unique Identifier: biotools:genesetcart

Funding

This project was supported by NIH grants R01DK131525, OT2OD036435, OT2OD030160, U24CA264250, U24CA271114, and RC2DK131995.

Competing interests

The authors declare that they have no competing interests.

Figure Legends

Figure 1. GeneSetCart gene set input and analysis features. A. Users can upload a single gene set or enter a gene set in a text area; Upload a .gmt file containing multiple gene sets; Search PubMed for any term, then the returned PMIDs are converted into sene set based on GeneRIF or AutoRIF; Fetch gene sets created from data collected by Common Fund programs; Fetch gene sets from Enrichr gene set libraries related to an input term. **B.** The Augment feature allows augmentation of a gene set with co-expressed genes or PPI; The Combine features provides mechanisms to perform set operations on gene sets to create new sets; The Visualize page contain tools to visualize the overlap among sets including: Venn diagrams, UpSet plots, SuperVenn, heatmaps and UMAP visualization; The Analyze page permits users to submit their gene sets to a collection of external tools; and the Report page provides means to generate reports using a selected set of sets, and tools for performing the analysis.

Figure 2. CFDE gene set libraries included in GeneSetCart. A. Gene set sizes created from various NIH Common Fund programs. **B.** UMAP plot of IDF vectors of gene sets across Common Fund gene set libraries excluding LINCS. **C.** UMAP plot of IDF vectors of gene sets across all NIH Common Fund gene set libraries. **D.** Percentage of significantly overlapping crossing pairs across all possible combinations of gene set libraries.

Figure 3. Exploring shared pathways implicated in aging and exercise from blood. A. Workflow diagram depicting the analysis steps of cross the gene set libraries created from GTEx and MoTrPAC. **B.** Screenshot from the Venn diagram visualization created by GeneSetCart of the overlap between the top two crossing results of GTEx Aging Signatures vs MoTrPAC Rat Endurance Training libraries. **C.** Enrichment analysis of the 24 overlapping genes from the top two crossing with the KEGG 2021 Human gene set library in Enrichr. **D.** Enrichment analysis of the top 24 overlapping genes with the GO Biological Processes library in Enrichr. **E.** Publication count associated with all identified genes colored by set source. **F.** Publication for each gene colored by co-mentions with the terms aging, exercise, both, and neither.

Figure 4. Investigating mechanisms in Alexander's Disease. A. Use case workflow and analysis steps. **B.** Top 10 enriched terms from ChEA 2022 library of consensus up signature (n=3) gene set in Enrichr. **C.** Top 10 enriched terms from WikiPathway 2023 Mouse library of consensus up signature (n=3) gene set in Enrichr. **D.** Top 10 enriched terms from MGI Mammalian Phenotype Level 4 2024 library of consensus up signature gene set in Enrichr.

Figure S1. API response time (n=5) as a function of gene set size for all linked analysis tools.

Figure S2. P-value distribution of significant library crossing results for each library **A.** Glygen Glycosylated Proteins vs. rest. **B.** GTEx Aging Signatures vs. rest. **C.** GTEx Tissue Gene Expression Profiles vs. rest. **D.** IDG Drug Targets vs. rest. **E.** KOMP2 Mouse Phenotypes vs. rest. **F.** MoTrPAC Rat Endurance Training vs. rest. **G.** Metabolomics Workbench Metabolites. **H.** HuBMAP Azimuth 2023 Augmented. **I.** LINCS L1000 Chemical Perturbation Consensus Signatures. **J.** LINCS L1000 CRISPR Knockout Consensus Signatures.

Figure S3. Hypothesis generation for crossing the MoTrPAC exercise signatures with the GTEx tissue aging signatures. A. The CFDE GMT Crossing page results of crossing the GTEx Tissue-Specific Aging Signatures with the MoTrPAC Rat Endurance Training libraries. **B.** Overlapping genes with the lowest p-values for gene set pair overlap. **C.** GPT-4 generated hypothesis of lowest p-value gene set pair.

References

1. Subramanian A, Tamayo P, Mootha VK, Mukherjee S, Ebert BL, Gillette MA, et al.. Gene set enrichment analysis: a knowledge-based approach for interpreting genome-wide expression profiles. *Proc Natl Acad Sci U S A*. 102:15545–502005;

2. Ma'ayan A, Rouillard AD, Clark NR, Wang Z, Duan Q, Kou Y. Lean Big Data integration in systems biology and systems pharmacology. *Trends Pharmacol Sci*. Elsevier BV; 35:450–602014;

3. Rouillard AD, Gundersen GW, Fernandez NF, Wang Z, Monteiro CD, McDermott MG, et al.. The harmonizome: a collection of processed datasets gathered to serve and mine knowledge about genes and proteins. *Database* . 2016; doi: 10.1093/database/baw100.

4. Chen EY, Tan CM, Kou Y, Duan Q, Wang Z, Meirelles GV, et al.. Enrichr: interactive and collaborative HTML5 gene list enrichment analysis tool. *BMC Bioinformatics*. 14:1282013;

5. Reimand J, Kull M, Peterson H, Hansen J, Vilo J. g:Profiler--a web-based toolset for functional profiling of gene lists from large-scale experiments. *Nucleic Acids Res*. 35:W193-2002007;

6. Sherman BT, Hao M, Qiu J, Jiao X, Baseler MW, Lane HC, et al.. DAVID: a web server for functional enrichment analysis and functional annotation of gene lists (2021 update). *Nucleic Acids Res*. 50:W216–212022;

7. Elizarraras JM, Liao Y, Shi Z, Zhu Q, Pico AR, Zhang B. WebGestalt 2024: faster gene set analysis and new support for metabolomics and multi-omics. *Nucleic Acids Res*. Oxford Academic; 52:W415–212024;

8. Chen J, Bardes EE, Aronow BJ, Jegga AG. ToppGene Suite for gene list enrichment analysis and candidate gene prioritization. *Nucleic Acids Res*. 37:W305-112009;

9. Zhou Y, Zhou B, Pache L, Chang M, Khodabakhshi AH, Tanaseichuk O, et al.. Metascape provides a biologist-oriented resource for the analysis of systems-level datasets. *Nat Commun*. 10:15232019;

10. Karatzas E, Baltoumas FA, Aplakidou E, Kontou PI, Stathopoulos P, Stefanis L, et al.. Flame (v2.0): advanced integration and interpretation of functional enrichment results from multiple sources. *Bioinformatics*. 2023; doi: 10.1093/bioinformatics/btad490.

11. Schölz C, Lyon D, Refsgaard JC, Jensen LJ, Choudhary C, Weinert BT. Avoiding abundance bias in the functional annotation of post-translationally modified proteins. *Nat Methods*. 12:1003–42015;

12. Zhang B, Kirov S, Snoddy J. WebGestalt: an integrated system for exploring gene sets in various biological contexts. *Nucleic Acids Res*. 33:W741-82005;

13. Khan A, Mathelier A. Intervene: a tool for intersection and visualization of multiple gene or genomic region sets. *BMC Bioinformatics*. 18:2872017;

14. Yang M, Chen T, Liu Y-X, Huang L. Visualizing set relationships: EVenn's comprehensive approach to Venn diagrams. *Imeta*. 3:e1842024;

15. Shen L. GeneOverlap: An R package to test and visualize gene overlaps. *R Package*. bioconductor.statistik.tu-dortmund.de; 2016;

16. Clough E, Barrett T, Wilhite SE, Ledoux P, Evangelista C, Kim IF, et al.. NCBI GEO: archive for gene expression and epigenomics data sets: 23-year update. *Nucleic Acids Res*. 52:D138–442024;

17. Clarke DJB, Marino GB, Deng EZ, Xie Z, Evangelista JE, Ma'ayan A. Rummagene: massive mining of gene sets from supporting materials of biomedical research publications. *Commun Biol*. 7:4822024;

18. Marino GB, Clarke DJB, Lachmann A, Deng EZ, Ma'ayan A. RummaGEO: Automatic mining of human and mouse gene sets from GEO. *Patterns (N Y)*. Elsevier BV; 5:1010722024;

19. Lachmann A, Schilder BM, Wojciechowicz ML, Torre D, Kuleshov MV, Keenan AB, et al.. Geneshot: search engine for ranking genes from arbitrary text queries. *Nucleic Acids Res*. academic.oup.com; 47:W571–72019;

20. Lachmann A, Torre D, Keenan AB, Jagodnik KM, Lee HJ, Wang L, et al.. Massive mining of publicly available RNA-seq data from human and mouse. *Nat Commun*. 9:13662018;

21. Berger SI, Posner JM, Ma'ayan A. Genes2Networks: connecting lists of gene symbols using mammalian protein interactions databases. *BMC Bioinformatics*. Springer; 8:3722007;

22. Breitkreutz B-J, Stark C, Tyers M. The GRID: The General Repository for Interaction Datasets. *Genome Biol*. Genome Biol; 4:R232003;

23. Huttlin EL, Ting L, Bruckner RJ, Gebreab F, Gygi MP, Szpyt J, et al.. The BioPlex network: A systematic exploration of the human interactome. *Cell*. Elsevier BV; 162:425–402015;

24. Hermjakob H, Montecchi-Palazzi L, Lewington C, Mudali S, Kerrien S, Orchard S, et al.. IntAct: an open source molecular interaction database. *Nucleic Acids Res*. Oxford University Press (OUP); 32:D452-52004;

25. Zanzoni A, Montecchi-Palazzi L, Quondam M, Ausiello G, Helmer-Citterich M, Cesareni G. MINT: A molecular INTeraction database. *FEBS Lett*. Wiley; 513:135–402002;

26. Husi H, Grant SGN. Construction of a protein-protein interaction database (PPID) for synaptic biology. *Neuroscience Databases*. Boston, MA: Springer US; p. 51–62.

27. Turner B, Razick S, Turinsky AL, Vlasblom J, Crowdy EK, Cho E, et al.. iRefWeb: interactive analysis of consolidated protein interaction data and their supporting evidence. *Database (Oxford)*. Oxford Academic; 2010:baq0232010;

28. Stelzl U, Worm U, Lalowski M, Haenig C, Brembeck FH, Goehler H, et al.. A human protein-protein interaction network: a resource for annotating the proteome. *Cell*. Elsevier BV; 122:957–682005;

29. McInnes L, Healy J. UMAP: Uniform Manifold Approximation and Projection for Dimension Reduction. *arXiv.org*. 2018;

30. . reaviz: □ Data visualization library for React. Maintained by @goodcodeus. Github;

31. Bostock M, Ogievetsky V, Heer J. D^3: Data-Driven Documents. *IEEE Trans Vis Comput Graph*. ieeexplore.ieee.org; 17:2301–92011;

32. . react_supervenn at main · MaayanLab/react-supervenn. Github;

33. Fedor. supervenn: supervenn: precise and easy-to-read multiple sets visualization in Python. Github;

34. Pedregosa F, Varoquaux G, Gramfort A, Michel V, Thirion B, Grisel O, et al.. Scikit-learn: Machine Learning in Python. *J Mach Learn Res*. jmlr.org; 2011; doi: 10.5555/1953048.2078195.

35. Wolf FA, Angerer P, Theis FJ. SCANPY: large-scale single-cell gene expression data analysis. *Genome Biol*. Springer; 19:152018;

36. Traag VA, Waltman L, van Eck NJ. From Louvain to Leiden: guaranteeing well-connected communities. *Sci Rep*. nature.com; 9:52332019;

37. Evangelista JE, Xie Z, Marino GB, Nguyen N, Clarke DJB, Ma'ayan A. Enrichr-KG: bridging enrichment analysis across multiple libraries. *Nucleic Acids Res*. 51:W168–792023;

38. Keenan AB, Torre D, Lachmann A, Leong AK, Wojciechowicz ML, Utti V, et al.. ChEA3: transcription factor enrichment analysis by orthogonal omics integration. *Nucleic Acids Res*. 47:W212–242019;

39. Kuleshov MV, Xie Z, London ABK, Yang J, Evangelista JE, Lachmann A, et al.. KEA3: improved kinase enrichment analysis via data integration. *Nucleic Acids Res*. 49:W304–162021;

40. Evangelista JE, Clarke DJB, Xie Z, Lachmann A, Jeon M, Chen K, et al.. SigCom LINCS: data and metadata search engine for a million gene expression signatures. *Nucleic Acids Res*. 50:W697–7092022;

41. Clarke DJB, Evangelista JE, Xie Z, Marino GB, Maurya M, Srinivasan S, et al.. Playbook Workflow Builder: Interactive construction of bioinformatics workflows from a network of microservices. *bioRxiv*. 2024; doi: 10.1101/2024.06.08.598037.

42. Keenan AB, Jenkins SL, Jagodnik KM, Koplev S, He E, Torre D, et al.. The Library of Integrated Network-Based Cellular Signatures NIH Program: System-Level Cataloging of Human Cells Response to Perturbations. *Cell Syst*. 6:13–242018;

43. Shin M-G, Pico A. Using Published Pathway Figures in Enrichment Analysis and Machine Learning. *bioRxiv*. 2023; doi: 10.1101/2023.07.06.548037.

44. Hanspers K, Riutta A, Summer-Kutmon M, Pico AR. Pathway information extracted from 25 years of pathway figures. *Genome Biol*. 21:2732020;

45. Gene Ontology Consortium. Gene Ontology Consortium: going forward. *Nucleic Acids Res*. 43:D1049-562015;

46. Kutmon M, Riutta A, Nunes N, Hanspers K, Willighagen EL, Bohler A, et al.. WikiPathways: capturing the full diversity of pathway knowledge. *Nucleic Acids Res*. 44:D488-942016;

47. Blake JA, Bult CJ, Eppig JT, Kadin JA, Richardson JE, Mouse Genome Database Group. The Mouse Genome Database genotypes::phenotypes. *Nucleic Acids Res*. 37:D712-92009;

48. Sollis E, Mosaku A, Abid A, Buniello A, Cerezo M, Gil L, et al.. The NHGRI-EBI GWAS Catalog: knowledgebase and deposition resource. *Nucleic Acids Res*. 51:D977–852023;

49. Oprea TI, Bologa CG, Brunak S, Campbell A, Gan GN, Gaulton A, et al.. Unexplored therapeutic opportunities in the human genome. *Nat Rev Drug Discov*. Nat Rev Drug Discov; 17:317–322018;

50. Sud M, Fahy E, Cotter D, Azam K, Vadivelu I, Burant C, et al.. Metabolomics Workbench: An international repository for metabolomics data and metadata, metabolite standards, protocols, tutorials and training, and analysis tools. *Nucleic Acids Res*. Oxford University Press (OUP); 44:D463-702016;

51. Dickinson ME, Flenniken AM, Ji X, Teboul L, Wong MD, White JK, et al.. High-throughput discovery of novel developmental phenotypes. *Nature*. Nature; 537:508–142016;

52. GTEx Consortium. The Genotype-Tissue Expression (GTEx) project. *Nat Genet*. Nat Genet; 45:580–52013;

53. York WS, Mazumder R, Ranzinger R, Edwards N, Kahsay R, Aoki-Kinoshita KF, et al.. GlyGen: Computational and informatics resources for glycoscience. *Glycobiology*. Oxford University Press (OUP); 30:72–32020;

54. HuBMAP Consortium. The human body at cellular resolution: the NIH Human Biomolecular Atlas Program. *Nature*. Springer Science and Business Media LLC; 574:187–922019;

55. Sanford JA, Nogiec CD, Lindholm ME, Adkins JN, Amar D, Dasari S, et al.. Molecular Transducers of Physical Activity Consortium (MoTrPAC): Mapping the dynamic responses to exercise. *Cell*. Elsevier BV; 181:1464–742020;

56. Virtanen P, Gommers R, Oliphant TE, Haberland M, Reddy T, Cournapeau D, et al.. SciPy 1.0: fundamental algorithms for scientific computing in Python. *Nat Methods*. 17:261–722020;

57. Wick G, Jansen-Dürr P, Berger P, Blasko I, Grubeck-Loebenstein B. Diseases of aging. *Vaccine*. 18:1567–832000;

58. Saul D, Kosinsky RL. Epigenetics of Aging and Aging-Associated Diseases. *Int J Mol Sci*. 2021; doi: 10.3390/ijms22010401.

59. Wilkerson HLC. Problems of an Aging Population. *Am J Public Health Nations Health*. American Public Health Association; 37:177–881947;

60. North BJ, Sinclair DA. The intersection between aging and cardiovascular disease. *Circ Res*. 110:1097–1082012;

61. Xia X, Jiang Q, McDermott J, Han J-DJ. Aging and Alzheimer's disease: Comparison and associations from molecular to system level. *Aging Cell*. 17:e128022018;

62. Reeve A, Simcox E, Turnbull D. Ageing and Parkinson's disease: why is advancing age the biggest risk factor? *Ageing Res Rev*. 14:19–302014;

63. Caspersen CJ, Powell KE, Christenson GM. Physical activity, exercise, and physical fitness: definitions and distinctions for health-related research. *Public Health Rep*. 100:126–311985;

64. Fiuza-Luces C, Santos-Lozano A, Joyner M, Carrera-Bastos P, Picazo O, Zugaza JL, et al.. Exercise benefits in cardiovascular disease: beyond attenuation of traditional risk factors. *Nat Rev Cardiol*. 15:731–432018;

65. Schenk S, Sagendorf TJ, Many GM, Lira A, DeSousa G, Bae D, et al.. Physiological Adaptations to Progressive Endurance Exercise Training in Adult And Aged Rats: Insights from The Molecular Transducers of Physical Activity Consortium (MoTrPAC). *Function*. doi: 10.1093/function/zqae014.

66. Tracy RP, Bovill EG. Thrombosis and cardiovascular risk in the elderly. *Arch Pathol Lab Med*. 116:1307–121992;

67. El-Sayed MS, Sale C, Jones PG, Chester M. Blood hemostasis in exercise and training. *Med Sci Sports Exerc*. 32:918–252000;

68. Johnson AA, Stolzing A. The role of lipid metabolism in aging, lifespan regulation, and age-related disease. *Aging Cell*. 18:e130482019;

69. Prospective Studies Collaboration, Lewington S, Whitlock G, Clarke R, Sherliker P, Emberson J, et al.. Blood cholesterol and vascular mortality by age, sex, and blood pressure: a meta-analysis of individual data from 61 prospective studies with 55,000 vascular deaths. *Lancet*. 370:1829–392007;

70. Horowitz JF, Klein S. Lipid metabolism during endurance exercise. *Am J Clin Nutr*. 72:558S-63S2000;

71. Carapeto PV, Aguayo-Mazzucato C. Effects of exercise on cellular and tissue aging. *Aging* . 13:14522–432021;

72. Kuhn J, Cascella M. Alexander Disease. StatPearls Publishing;

73. Messing A, Head MW, Galles K, Galbreath EJ, Goldman JE, Brenner M. Fatal encephalopathy with astrocyte inclusions in GFAP transgenic mice. *Am J Pathol*. 152:391–81998;

74. Barrett T, Wilhite SE, Ledoux P, Evangelista C, Kim IF, Tomashevsky M, et al.. NCBI GEO: archive for functional genomics data sets--update. *Nucleic Acids Res*. 41:D991-52013;

75. Gammie SC, Messing A, Hill MA, Kelm-Nelson CA, Hagemann TL. Large-scale gene expression changes in APP/PSEN1 and GFAP mutation models exhibit high congruence with Alzheimer's disease. *PLoS One*. 19:e02919952024;

76. Ritchie ME, Phipson B, Wu D, Hu Y, Law CW, Shi W, et al.. limma powers differential expression analyses for RNA-sequencing and microarray studies. *Nucleic Acids Res*.

43:e472015;

77. Clarke DJB, Jeon M, Stein DJ, Moiseyev N, Kropiwnicki E, Dai C, et al.. Appyters: Turning Jupyter Notebooks into data-driven web apps. *Patterns (N Y)*. 2:1002132021;

78. Slota JA, Medina SJ, Frost KL, Booth SA. Neurons and astrocytes elicit brain region specific transcriptional responses to prion disease in the Murine CA1 and thalamus. *Front Neurosci*. Frontiers Media SA; 16:9188112022;

79. Crespo I, Roomp K, Jurkowski W, Kitano H, del Sol A. Gene regulatory network analysis supports inflammation as a key neurodegeneration process in prion disease. *BMC Syst Biol*. Springer Science and Business Media LLC; 6:1322012;

80. Zhang X, Lan Y, Xu J, Quan F, Zhao E, Deng C, et al.. CellMarker: a manually curated resource of cell markers in human and mouse. *Nucleic Acids Res*. Oxford University Press (OUP); 47:D721–82019;

81. Tabula Muris Consortium. A single-cell transcriptomic atlas characterizes ageing tissues in the mouse. *Nature*. Springer Science and Business Media LLC; 583:590–52020;

82. Franzén O, Gan L-M, Björkegren JLM. PanglaoDB: a web server for exploration of mouse and human single-cell RNA sequencing data. *Database (Oxford)*. Oxford University Press (OUP); 2019; doi: 10.1093/database/baz046.

83. Shen EH, Overly CC, Jones AR. The Allen Human Brain Atlas: comprehensive gene expression mapping of the human brain. *Trends Neurosci*. Elsevier BV; 35:711–42012;

Figure 1

Click here to access/download;Figure;fig1.pdf

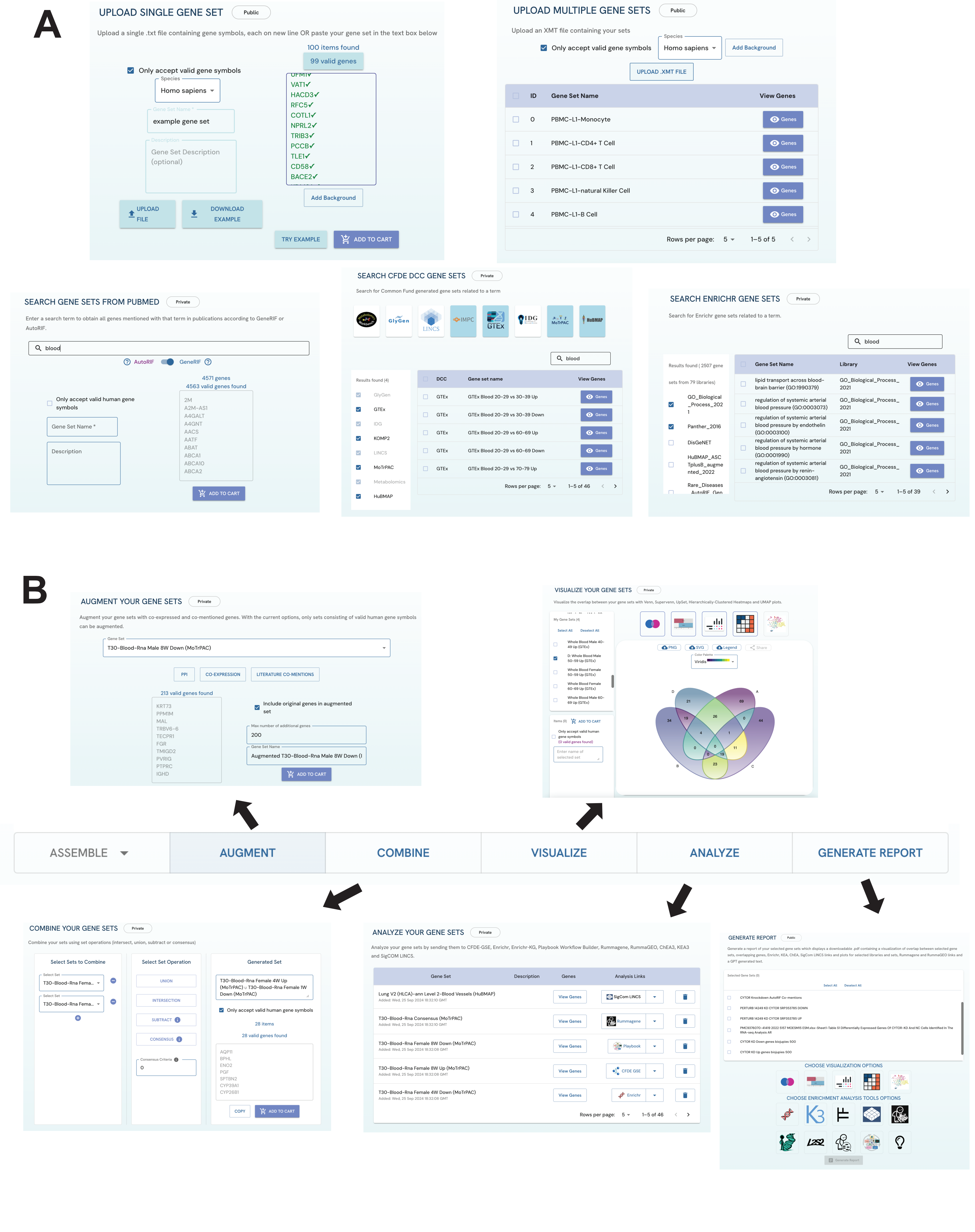

Figure 2

Click here to access/download;Figure;fig2.pdf

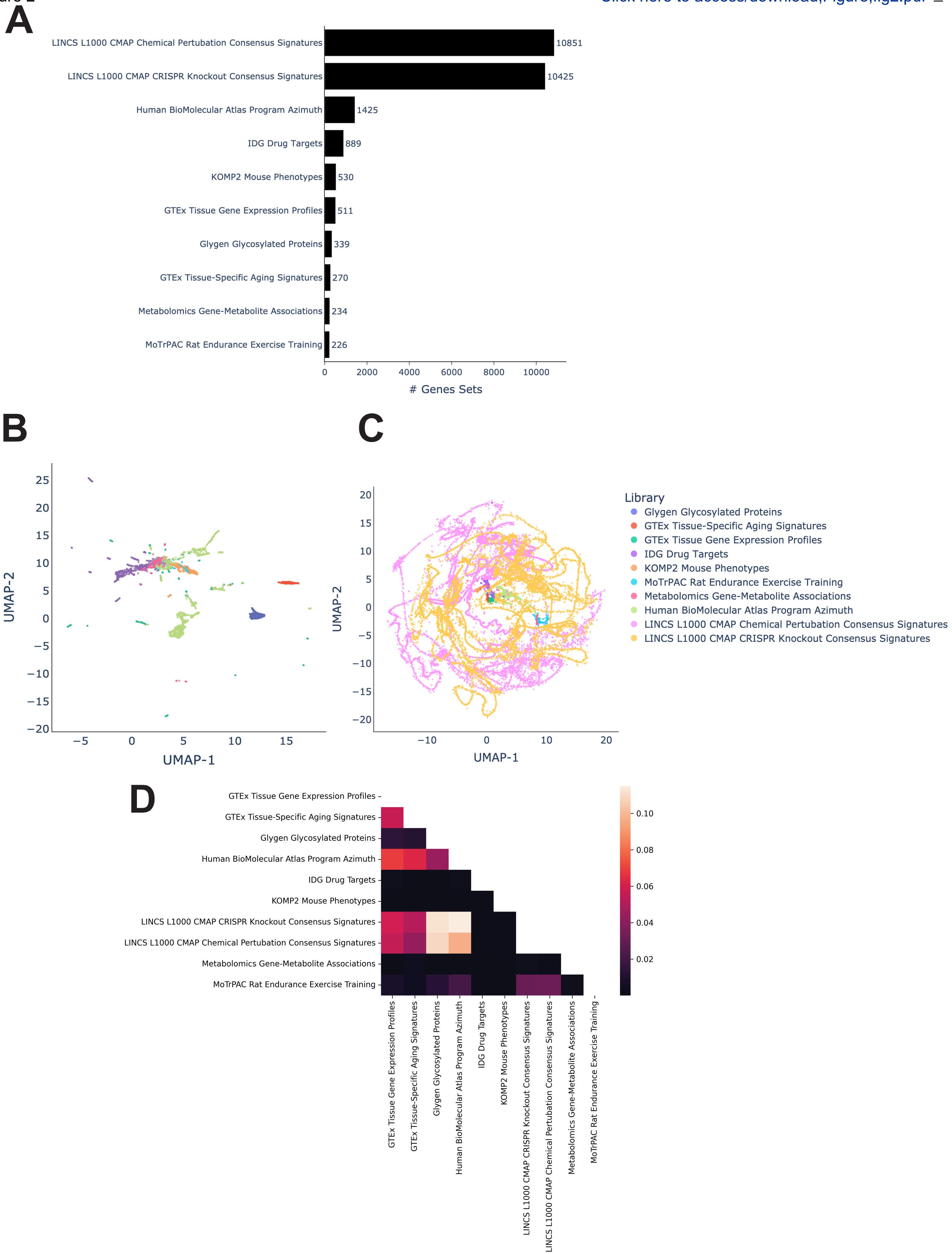

Figure 3

Click here to access/download;Figure;fig3.pdf

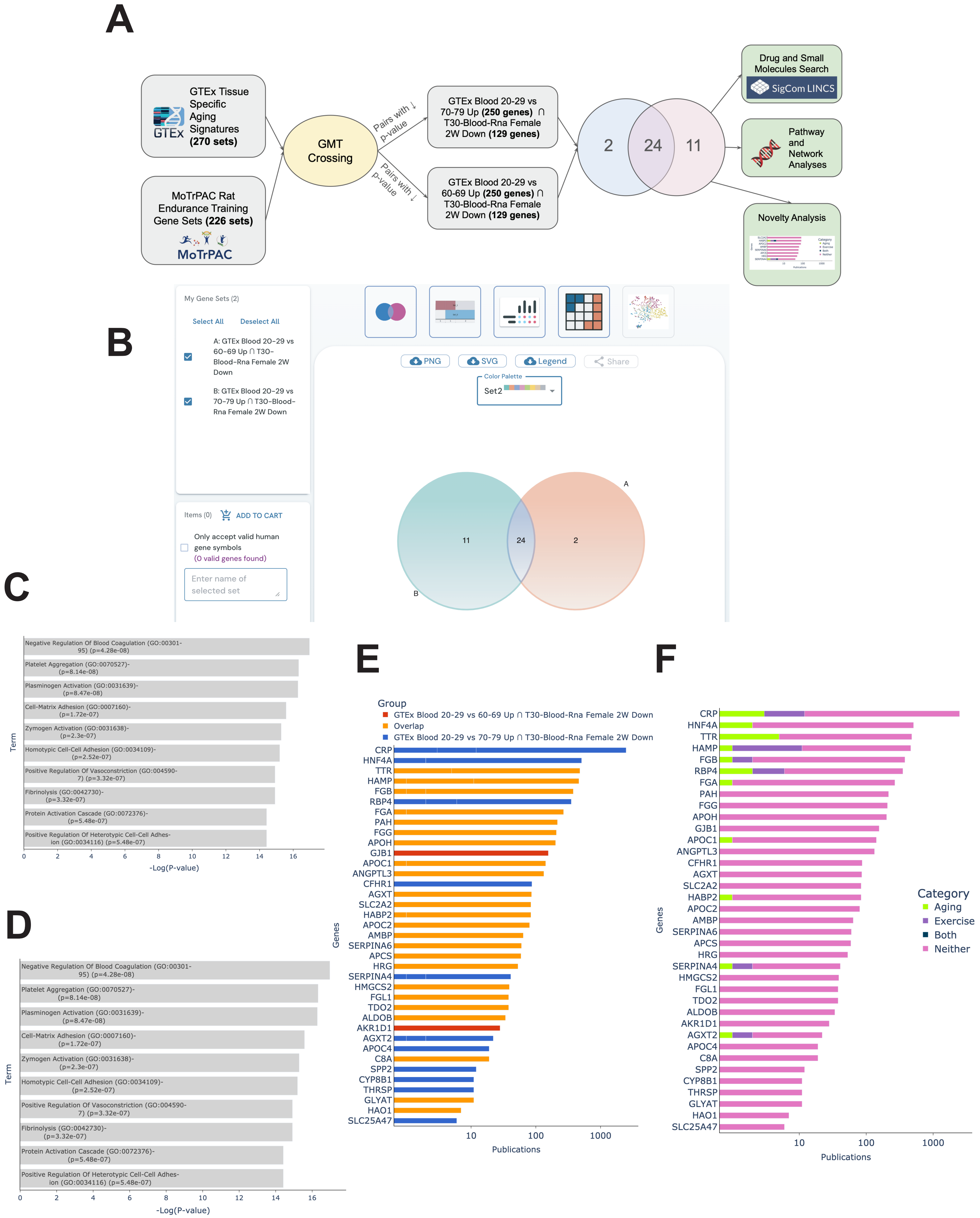

Figure 3

Click here to access/download;Figure;fig4.pdf

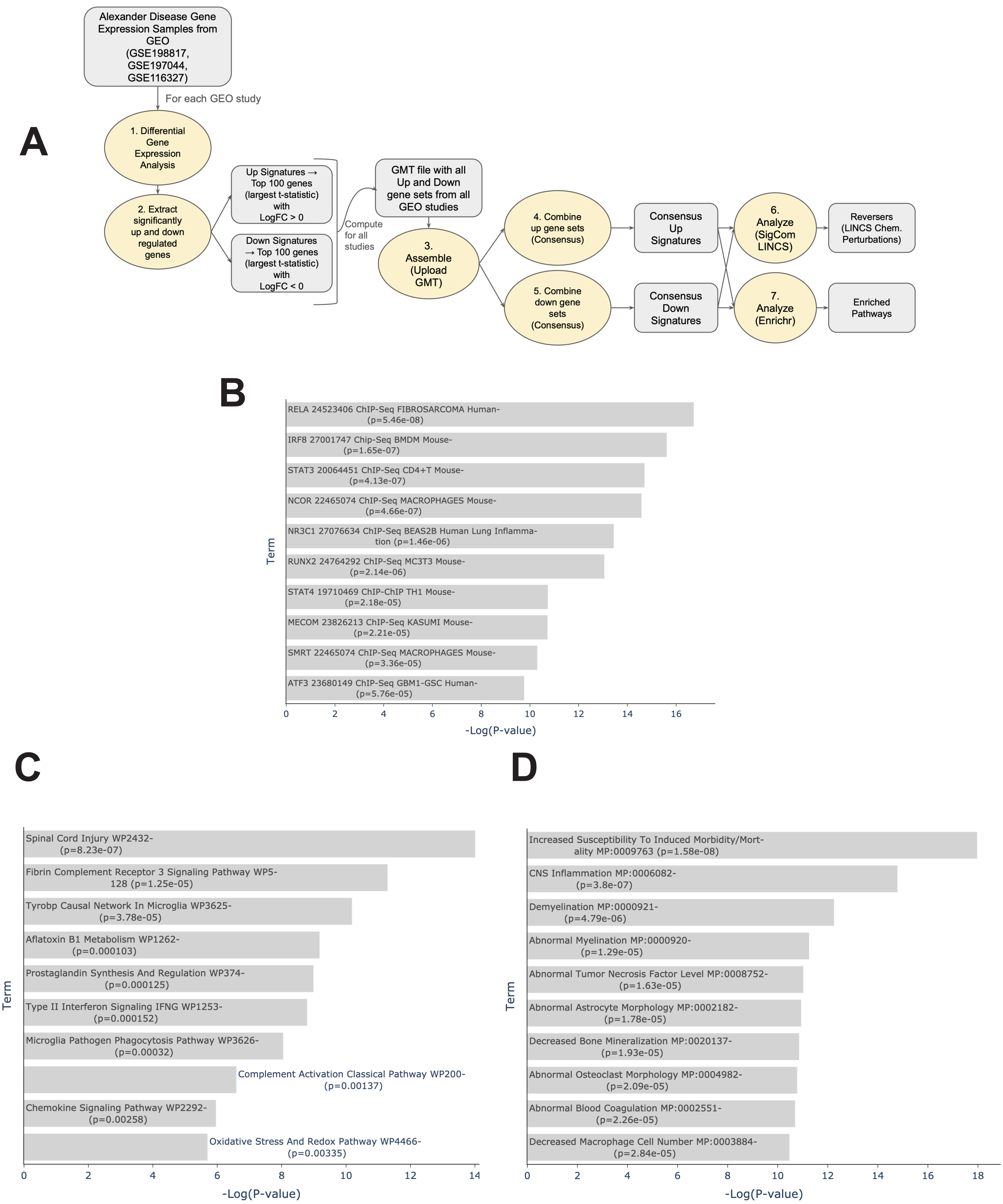

Figure S1

Click here to access/download;Figure;figS1.pdf

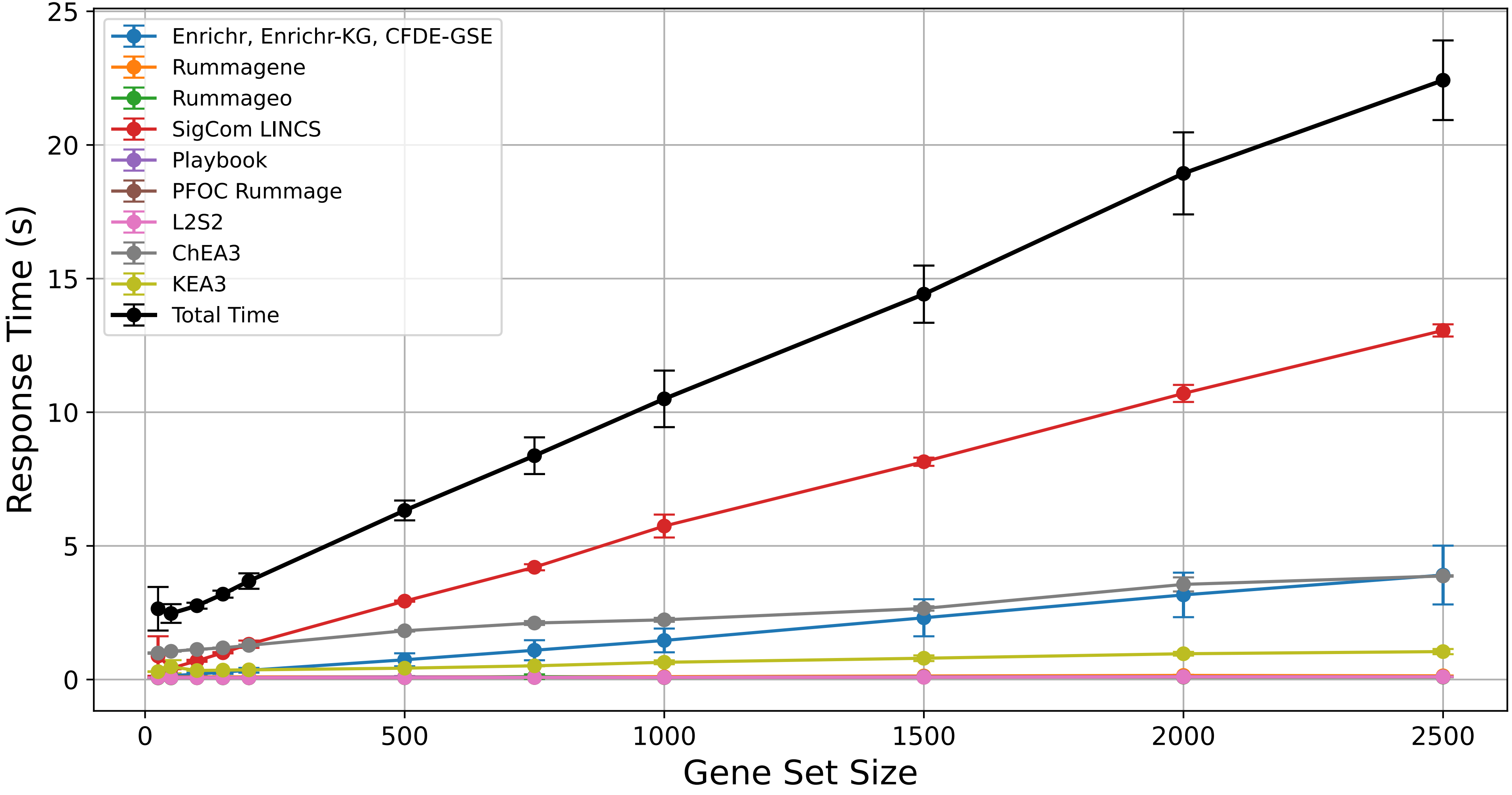

Figure S2

Click here to access/download;Figure;figS2.pdf

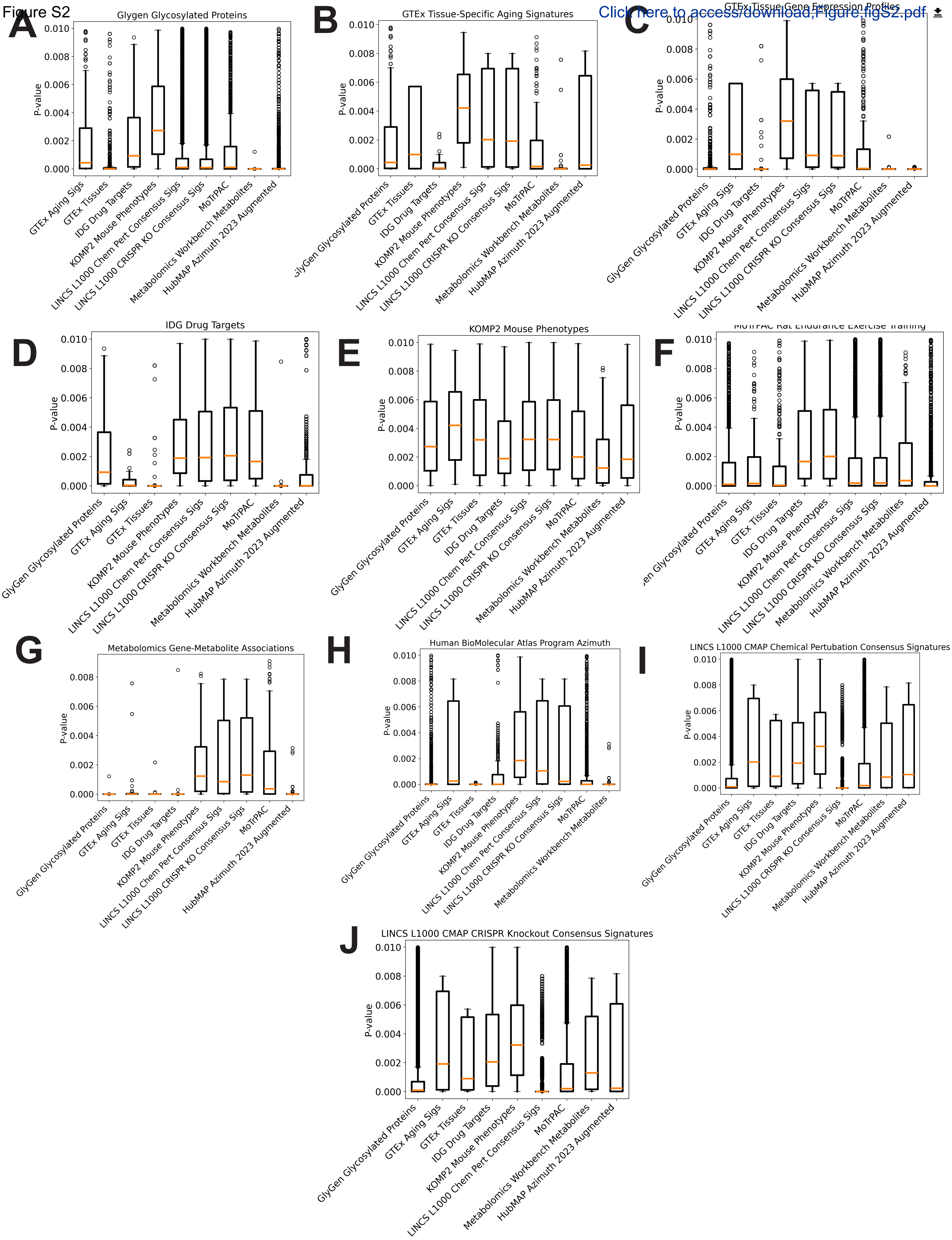

Figure S3 Click here to access/download;Figure;figS3.pdf

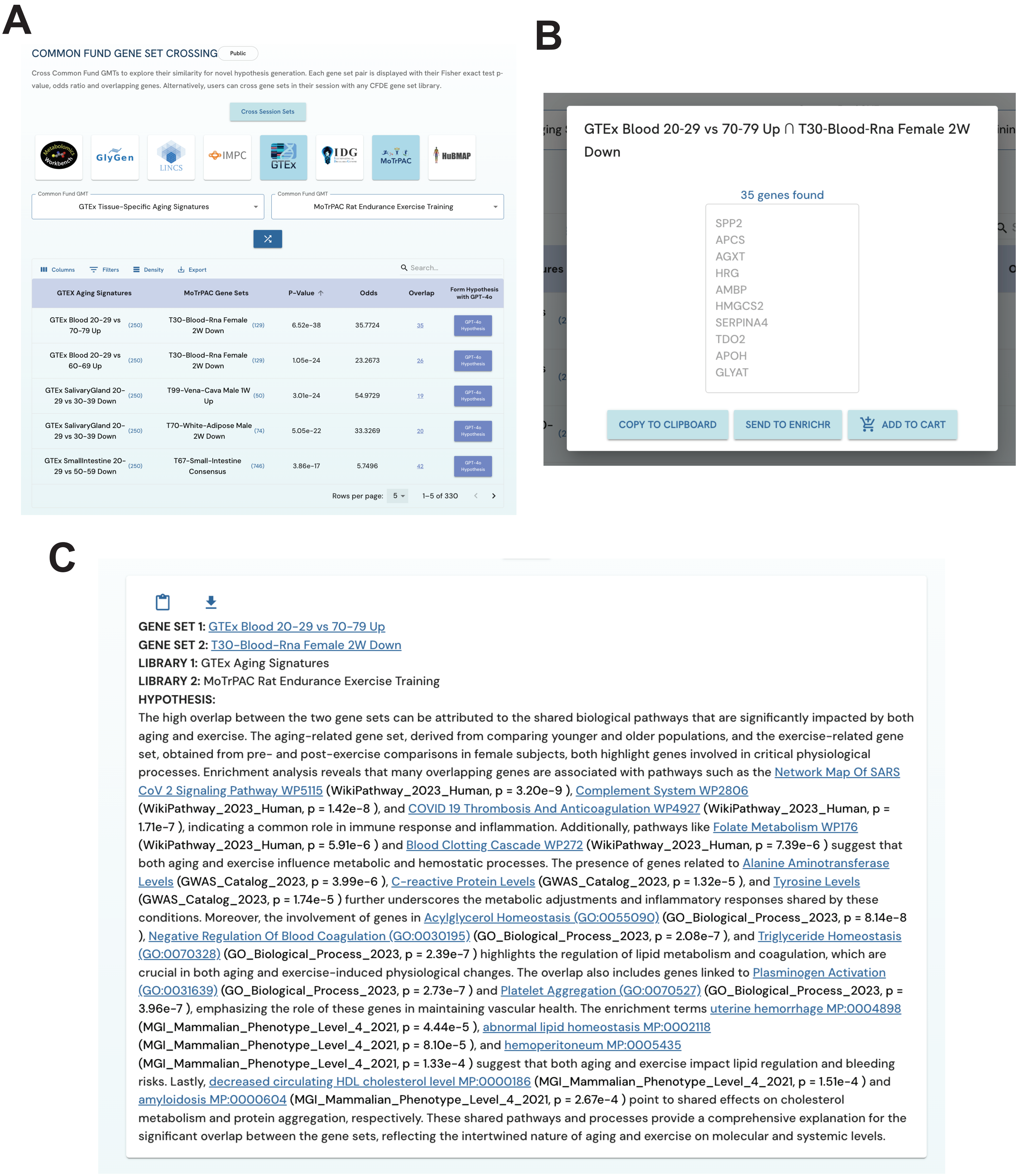

Supplement: giaf025_GIGA-D-24-00490_Revision_1 [file giaf025_giga-d-24-00490_revision_1.pdf]
