# Supplementary material for: GeneSetCart: assembling, augmenting, combining, visualizing, and analyzing gene sets
Source: Gigascience. 2025 Apr 10;14:giaf025. doi: 10.1093/gigascience/giaf025 (PMC11984350; doi:10.1093/gigascience/giaf025)
Supplement: giaf025_Response_to_Reviewer_Comments_Original_Submission [file giaf025_response_to_reviewer_comments_original_submission.pdf]

Author's Response To Reviewer Comments

GeneSetCart - Replies to the Reviewers

Replies to Reviewer 1

Reviewer #1: GeneSetCart is a web-based platform for collection and analysis of gene sets from various sources. It will be useful for omics-focused researchers to understand their datasets. The introduction gives a good level of background information, especially around cloud based gene set collection and analysis tools with an emphasis on user friendly tools. It is made clear that GeneSetCart is a tool that is complementary to existing ones and builds on top of the team's existing portfolio including Enrichr, RummaGEO, ARCHS4, Harmonizome and other tools. The app looks like it was professionally designed, works smoothly and integrates well with other tools. Overall the manuscript is very well written, the app is thoroughly described and the case studies help to illuminate the potential of this type of analysis as well as identifying some interesting insights into aging and Alexander disease. I did find a few points in the manuscript that need further clarification.

Reply: Thank you for your positive and supportive comments about the manuscript and software application.

Reviewer #1: Which Species are supported? Is it possible to convert gene names based on ortholog relationships to facilitate cross-species comparisons? While the focus is on human and mouse, I can foresee this resource being extensively used by plant, animal and microbial researchers.

Reply: Thank you for this suggestion. The GeneSetCart platform mainly facilitates the analysis of human gene symbols, though it can accept any generic symbols or identifiers. To better accommodate additional species, we have added the ability to validate and standardize gene symbols for 18 species within the platform. Furthermore, each set is associated with an orthologous set of human gene symbols which are used for the downstream analysis tools because these tools mainly only accept human gene symbols.

Reviewer #1: Regarding gene identifiers - accession numbers are unique, but symbols/names are descriptive. Names also change over time. How is this managed? Will outdated gene symbols be updated over time? Will the tool be able to recognise Excel gene-date autocorrect errors? Can the tool ingest accessions from different sources like Ensembl and Entrez?

Reply: Thank you for this comment. We will utilize and keep up-to-date with the latest official NCBI gene symbols. Genes are stored in the database by their NCBI gene ID which are unique for each gene and can be associated with any changes to gene symbols over time. For the revised manuscript, we now added the functionality to recognize and correct Excel gene-date errors and other mismatching gene names. The NCBI gene information ingested into the GeneSetCart database also includes gene synonyms from Ensembl, Entrez, HGNC, OMIM, and previous symbols used by NCBI. These synonyms are utilized to map user-inputted genes into their standardized NCBI gene symbols supported by GeneSetCart.

Reviewer #1: As users will be uploading and working with gene sets, which could represent some important IP, the privacy and IP considerations might need some further clarity. Will gene sets and data remain private? What assurances are there that the data will remain private? Who retains IP ownership of the uploaded gene signatures and the downstream works? What about the data sent to ChatGPT/OpenAI? Such details could be described in a terms of service document, which the user can retain for their records.

Reply: Thank you for this comment. Overall, sessions created by logged-in users who may upload their own gene sets are private by default. We have no intention of sharing gene sets without user consent. Users retain the IP ownership of their uploaded gene sets and their analysis using our tools. We can't make such assurances about gene sets and other text the user indirectly sends to OpenAI. For the revised manuscript, we added text to the manuscript about our policy. We also added a Terms of Service section to the GeneSetCart About page to cover these concerns.

Reviewer #1: Fig 2A needs an x-axis label.

Reply: Thank you for pointing this out. The x-axis label has been added.

Reviewer #1: Gene sets are truly useful in distilling large genomic datasets into a manageable size, but it should be emphasised that the Genes of Interest list (GoI) is influenced by the limitations of the technology and biological material (Timmons 2015; PMID: 26346307). Therefore when doing downstream enrichment analysis of these gene sets, we should be using a background gene list that eliminates those biases. One way to overcome this is by systematically collecting background gene lists for each GoI list, that is,the complete list of genes/proteins that were detected in the assay. Including an option for a background list for every GoI list would nudge the end user towards best practice. This point is relevant to the 24 gene enrichment shown in Fig 3B, where the background list correction could lead to half of the results being invalid (Wijesooriya 2022, PMID: 35263338, Fig 4C).

Reply: Thank you for this comment. We have added the ability to include a background gene set for each submitted gene set and utilize this background in any downstream tool that supports background. Since the overlap in Figure 3 reflects RNA-seq data, the background of all human protein-coding genes is appropriate and has been utilized.

Replies to Reviewer 2

Reviewer #2: In the submitted manuscript, the authors introduce GeneSetCart, a web-based platform designed to facilitate the management, augmentation, and analysis of gene sets from multiple sources, with a particular emphasis on those generated by NIH Common Fund programs. This platform has the potential to be highly useful for experimental biologists who often struggle with fetching and analyzing gene sets across diverse NIH web portals.

Reply: Thank you for your positive and supportive comments about the manuscript and software application.

Reviewer #2: The “Only Accept Valid Human Gene Symbols” option, if not selected at the beginning, can result in certain operations during the AUGMENT phase—such as selecting specific gene sets—failing. Highlighting this option prominently at the start would prevent invalid user operations and enhance usability.

Reply: Thank you for this comment. We have more prominently highlighted this option and added additional tools to convert gene symbols provided by the user into validated gene sets.

Reviewer #2: GeneSetCart offers three augmentation methods: PPI, Co-Expression, and Literature Co-Mentions. However, the PPI method seems to provide minimal augmentation compared to the other two. For example, applying PPI to the “GSEA: HALLMARK_IL2_STAT5_SIGNALING” human gene set added only three new genes, resulting in 196 genes, compared to 376 and 379 genes augmented by Co-Expression and Literature Co-Mentions, respectively.
- The authors should justify the inclusion of PPI as an augmentation method, especially given its relatively limited contribution.
- Alternatively, they could consider integrating enrichment tools specifically tailored for network data in the ANALYSIS phase, where high-quality protein-protein interaction data could play a more critical role.

Reply: Thank you for this comment. The default parameters to the PPI expansion augmentation method have been updated to provide a more comprehensive background of genes based on a larger integrated protein-protein interactions database. After implementing this upgrade, ~200 genes are added by all three augmentation methods.

Reviewer #2: GeneSetCart includes a wide range of analysis tools, such as CFDE-GSE, Enrichr, Enrichr-KG, Rummagene, RummaGEO, ChEA3, KEA3, SigCOM LINCS, L2S2, and PFOCRummage. However:
- Switching between these tools requires repeated manual navigation, which could be streamlined.

- Applying all tools generates a large volume of results, which might be overwhelming for experimental biologists to interpret.
Recommendation: Consider providing a batch-run option and integrating the results in an organized, user-friendly format to improve accessibility and usability.

Reply: Thank you for your suggestion. For the revised version of the application, we improved the way reports work. The updated reports not only provide a way to batch-run all the tools, but also display the results in a summary, and a way that enables the users to easily navigate to the sections of the report that are most relevant to them. We also made the reports persistent through a unique and persistent URL, added a table of contents, added a way to automatically generate an abstract, and we now also provide options for organizing the report by either analysis type or by gene set.

Reviewer #2: The platform’s report generation time varies significantly depending on the size of the input gene set. Larger gene sets result in substantially longer generation times. Given the potential use of the platform with large-scale datasets, the authors should assess and report the platform’s scalability.

Reply: Thank you for this comment and suggestion. For the revised version of the manuscript, we added a new figure (Fig. S1) that shows the average time it takes to analyze gene sets of various sizes with each of the reports’ tools, as well as executing the report with all the tools. This information can assist users with understanding the wait times for their submitted gene sets.

Reviewer #2: The GPT-4o feature for generating explanatory hypotheses is currently restricted to the COMMON FUND GENE SET CROSSING module. However, users cannot apply this functionality to their own gene set intersections, limiting its broader applicability.
- The authors should clarify the rationale behind this restriction.
- Additionally, they should discuss whether this functionality can be extended to user-defined gene sets to enhance usability and versatility.

Reply: Thank you for this suggestion. For this revision, we added the ability for users to examine the overlap between their own gene sets and gene sets from all the CFDE-originated gene set libraries. This new feature was added to the gene-set-crossing interface. Additionally, for the Report section, we added the ability to form GPT-4o LLM hypotheses speculating about the overlap between two gene sets from their session. To activate this feature, the user needs to select two gene sets. To implement this feature, we utilize the gene set descriptions, the overlapping genes, and the Enrichr results for each set. With this information, we ask GPT-4o to generate a hypothesis.
