# Supplementary material for: GeneSetCart: assembling, augmenting, combining, visualizing, and analyzing gene sets
Source: Gigascience. 2025 Apr 10;14:giaf025. doi: 10.1093/gigascience/giaf025 (PMC11984350; doi:10.1093/gigascience/giaf025)
Supplement: giaf025_Response_to_Reviewer_Comments_Revision_1 [file giaf025_response_to_reviewer_comments_revision_1.pdf]

Author's Response To Reviewer Comments

GeneSetCart - Replies to the Editor

Editor: In addition, please register any new software application in the databases to receive RRID (Research Resource Identification Initiative ID) and biotoolsID identifiers, and include these in your manuscript.

Reply: Both types of identifiers are now included in the manuscript.

GeneSetCart - Replies to the Reviewers

Replies to Reviewer 1

Reviewer #1: GeneSetCart is a web-based platform for collection and analysis of gene sets from various sources. It will be useful for omics-focused researchers to understand their datasets. The introduction gives a good level of background information, especially around cloud based gene set collection and analysis tools with an emphasis on user friendly tools. It is made clear that GeneSetCart is a tool that is complementary to existing ones and builds on top of the team's existing portfolio including Enrichr, RummaGEO, ARCHS4, Harmonizome and other tools. The app looks like it was professionally designed, works smoothly and integrates well with other tools. Overall the manuscript is very well written, the app is thoroughly described and the case studies help to illuminate the potential of this type of analysis as well as identifying some interesting insights into aging and Alexander disease. I did find a few points in the manuscript that need further clarificatioGood to hear that GeneSetCart has background capability now. Even though RNA-seq is quite comprehensive, using all protein coding genes is not appropriate for a background in this case. As the 24 genes of interest were detected in both the GTEx Blood and T30-Blood datasets, the background will need to consist of genes that were robustly detected in both datasets. Without a corresponding background, the enrichment analysis will only succeed in telling us that the gene list is blood derived. In blood, only ~12,000 genes are expressed, which is a subset of the ~20k protein coding genes encoded by the genome. Moreover, technical limitations with RNA-seq mean that some expressed genes will not be detected, so using a custom background list will be able to account for such sampling biases (whether biological or technological). To make the custom background list, please decide upon a detection threshold (such as no. raw counts, TPM, etc) and apply it to both GTEx Blood and T30-Blood datasets and get the intersection. Due to the widespread misunderstanding of this, I suggest citing Timmons 2015 somewhere in the article either as a part of the introduction, in the case study description, or as a limitation in the discussion.

Reply: Thank you for these suggestions. To address this concern, we added the following text to the revised manuscript:

(Lines 220-222)

"Since some of these external tools have the option to include a background for the enrichment analysis [45], users of GeneSetCart can upload such background sets or select from various provided backgrounds."

We also performed the enrichment analysis on the 24 blood genes with a blood gene set background we created from ARCHS4, and added this additional statement:

"Enrichment analysis applied to these 24 overlapping genes using Enrichr [4], with the default settings, and with a background set of genes expressed only in blood [45] using ARCHS4 as the background atlas [20],"

(Lines 341-343)

The results did not change the ordering of the enriched terms, but produced lower p-values as expected.

Reviewer #1: Another suggestion: when mentioning changes made to the MS in the rebuttal letter, page and line numbers are helpful for us to check that stated changes have been made. Document mark-up/highlighting works as well.

Reply: We added line number to the text as suggested.
