# Supplementary material for: GeneSetCart: assembling, augmenting, combining, visualizing, and analyzing gene sets
Source: Gigascience. 2025 Apr 10;14:giaf025. doi: 10.1093/gigascience/giaf025 (PMC11984350; doi:10.1093/gigascience/giaf025)
Supplement: giaf025_Reviewer_1_Report_Original_Submission — Mark Ziemann, PhD -- 12/5/2024 [file giaf025_reviewer_1_report_original_submission.pdf]

Reviewer Report

Title: GeneSetCart: Assembling, Augmenting, Combining, Visualizing, and Analyzing Gene Sets

Version: Original Submission **Date:** 12/5/2024

Reviewer name: Mark Ziemann, PhD

Reviewer Comments to Author:

GeneSetCart is a web based platform for collection and analysis of gene sets from various sources. It will be useful for omics-focused researchers to understand their datasets. The introduction gives a good level of background information, especially around cloud based gene set collection and analysis tools with an emphasis on user friendly tools. It is made clear that GeneSetCart is a tool that is complementary to existing ones and builds on top of the team's existing portfolio including Enrichr, RummaGEO, ARCHS4, Harmonizome and other tools. The app looks like it was professionally designed, works smoothly and integrates well with other tools. Overall the manuscript is very well written, the app is thoroughly described and the case studies help to illuminate the potential of this type of analysis as well as identifying some interesting insights into aging and Alexander disease. I did find a few points in the manuscript that need further clarification.

1. Which Species are supported? Is it possible to convert gene names based on ortholog relationships to facilitate cross-species comparisons? While the focus is on human and mouse, I can foresee this resource being extensively used by plant, animal and microbial researchers.
2. Regarding gene identifiers - accession numbers are unique, but symbols/names are descriptive. Names also change over time. How is this managed? Will outdated gene symbols be updated over time? Will the tool be able to recognise Excel gene-date autocorrect errors? Can the tool ingest accessions from different sources like Ensembl and Entrez?
3. As users will be uploading and working with gene sets, which could represent some important IP, the privacy and IP considerations might need some further clarity. Will gene sets and data remain private? What assurances are there that the data will remain private? Who retains IP ownership of the uploaded gene signatures and the downstream works? What about the data sent to ChatGPT/OpenAI? Such details could be described in a terms of service document, which the user can retain for their records.
4. Fig 2A needs an x-axis label.
5. Gene sets are truly useful in distilling large genomic datasets into a manageable size, but it should be emphasised that the Genes of Interest list (GoI) is influenced by the limitations of the technology and biological material (Timmons 2015; PMID: 26346307). Therefore when doing downstream enrichment analysis of these gene sets, we should be using a background gene list that eliminates those biases. One way to overcome this is by systematically collecting background gene lists for each GoI list, that is,the complete list of genes/proteins that were detected in the assay. Including an option for a background list for every GoI list would nudge the end user towards best practice. This point is relevant to the 24 gene enrichment shown in Fig 3B, where the background list correction could lead to half of the results being invalid (Wijesooriya 2022, PMID: 35263338, Fig 4C).

Methods

Are the methods appropriate to the aims of the study, are they well described, and are necessary controls included? Choose an item.

Conclusions

Are the conclusions adequately supported by the data shown? Choose an item.

Reporting Standards

Does the manuscript adhere to the journal's guidelines on minimum standards of reporting? Choose an item.

Choose an item.

Statistics

Are you able to assess all statistics in the manuscript, including the appropriateness of statistical tests used? Choose an item.

Quality of Written English

Please indicate the quality of language in the manuscript: Choose an item.

Declaration of Competing Interests

Please complete a declaration of competing interests, considering the following questions:

- Have you in the past five years received reimbursements, fees, funding, or salary from an organisation that may in any way gain or lose financially from the publication of this manuscript, either now or in the future?
- Do you hold any stocks or shares in an organisation that may in any way gain or lose financially from the publication of this manuscript, either now or in the future?
- Do you hold or are you currently applying for any patents relating to the content of the manuscript?
- Have you received reimbursements, fees, funding, or salary from an organization that holds or has applied for patents relating to the content of the manuscript?
- Do you have any other financial competing interests?
- Do you have any non-financial competing interests in relation to this paper?

If you can answer no to all of the above, write 'I declare that I have no competing interests' below. If your reply is yes to any, please give details below.

I declare that I have no competing interests.

I agree to the open peer review policy of the journal. I understand that my name will be included on my report to the authors and, if the manuscript is accepted for publication, my named report including any attachments I upload will be posted on the website along with the authors' responses. I agree for my report to be made available under an Open Access Creative Commons CC-BY license (http://creativecommons.org/licenses/by/4.0/). I understand that any comments which I do not wish to be included in my named report can be included as confidential comments to the editors, which will not be published.

Choose an item.

To further support our reviewers, we have joined with Publons, where you can gain additional credit to further highlight your hard work (see: https://publons.com/journal/530/gigascience). On publication of this paper, your review will be automatically added to Publons, you can then choose whether or not to claim your Publons credit. I understand this statement.

Yes Choose an item.
