# Supplementary material for: GeneSetCart: assembling, augmenting, combining, visualizing, and analyzing gene sets
Source: Gigascience. 2025 Apr 10;14:giaf025. doi: 10.1093/gigascience/giaf025 (PMC11984350; doi:10.1093/gigascience/giaf025)
Supplement: giaf025_Reviewer_1_Report_Revision_1 — Mark Ziemann, PhD -- 2/4/2025 [file giaf025_reviewer_1_report_revision_1.pdf]

Reviewer Report

Title: GeneSetCart: Assembling, Augmenting, Combining, Visualizing, and Analyzing Gene Sets

Version: Revision 1 **Date:** 2/4/2025

Reviewer name: Mark Ziemann, PhD

Reviewer Comments to Author:

1. Resolved. Optional suggestion - to include the species names here.
2. Resolved.
3. Resolved.
4. Resolved.
5. Good to hear that GeneSetCart has background capability now. Even though RNA-seq is quite comprehensive, using all protein coding genes is not appropriate for a background in this case. As the 24 genes of interest were detected in both the GTEx Blood and T30-Blood datasets, the background will need to consist of genes that were robustly detected in both datasets. Without a corresponding background, the enrichment analysis will only succeed in telling us that the gene list is blood derived. In blood, only ~12,000 genes are expressed, which is a subset of the ~20k protein coding genes encoded by the genome. Moreover, technical limitations with RNA-seq mean that some expressed genes will not be detected, so using a custom background list will be able to account for such sampling biases (whether biological or technological). To make the custom background list, please decide upon a detection threshold (such as no. raw counts, TPM, etc) and apply it to both GTEx Blood and T30-Blood datasets and get the intersection. Due to the widespread misunderstanding of this, I suggest citing Timmons 2015 somewhere in the article either as a part of the introduction, in the case study description, or as a limitation in the discussion.

Another suggestion: when mentioning changes made to the MS in the rebuttal letter, page and line numbers are helpful for us to check that stated changes have been made. Document mark-up/highlighting works as well.

Methods

Are the methods appropriate to the aims of the study, are they well described, and are necessary controls included? Choose an item.

Conclusions

Are the conclusions adequately supported by the data shown? Choose an item.

Reporting Standards

Does the manuscript adhere to the journal’s guidelines on minimum standards of reporting? Choose an item.

Choose an item.

Statistics

Are you able to assess all statistics in the manuscript, including the appropriateness of statistical tests used? Choose an item.

Quality of Written English

Please indicate the quality of language in the manuscript: Choose an item.

Declaration of Competing Interests

Please complete a declaration of competing interests, considering the following questions:

- Have you in the past five years received reimbursements, fees, funding, or salary from an organisation that may in any way gain or lose financially from the publication of this manuscript, either now or in the future?
- Do you hold any stocks or shares in an organisation that may in any way gain or lose financially from the publication of this manuscript, either now or in the future?
- Do you hold or are you currently applying for any patents relating to the content of the manuscript?
- Have you received reimbursements, fees, funding, or salary from an organization that holds or has applied for patents relating to the content of the manuscript?
- Do you have any other financial competing interests?
- Do you have any non-financial competing interests in relation to this paper?

If you can answer no to all of the above, write 'I declare that I have no competing interests' below. If your reply is yes to any, please give details below.

I declare that I have no competing interests.

I agree to the open peer review policy of the journal. I understand that my name will be included on my report to the authors and, if the manuscript is accepted for publication, my named report including any attachments I upload will be posted on the website along with the authors' responses. I agree for my report to be made available under an Open Access Creative Commons CC-BY license (http://creativecommons.org/licenses/by/4.0/). I understand that any comments which I do not wish to be included in my named report can be included as confidential comments to the editors, which will not be published.

Choose an item.

To further support our reviewers, we have joined with Publons, where you can gain additional credit to further highlight your hard work (see: https://publons.com/journal/530/gigascience). On publication of this paper, your review will be automatically added to Publons, you can then choose whether or not to claim your Publons credit. I understand this statement.

Yes Choose an item.
