# Supplementary material for: GeneSetCart: assembling, augmenting, combining, visualizing, and analyzing gene sets
Source: Gigascience. 2025 Apr 10;14:giaf025. doi: 10.1093/gigascience/giaf025 (PMC11984350; doi:10.1093/gigascience/giaf025)
Supplement: giaf025_Reviewer_2_Report_Original_Submission — Weidong Tian -- 12/23/2024 [file giaf025_reviewer_2_report_original_submission.pdf]

Reviewer Report

Title: GeneSetCart: Assembling, Augmenting, Combining, Visualizing, and Analyzing Gene Sets

Version: Original Submission **Date:** 12/23/2024

Reviewer name: Weidong Tian

Reviewer Comments to Author:

In the submitted manuscript, the authors introduce GeneSetCart, a web-based platform designed to facilitate the management, augmentation, and analysis of gene sets from multiple sources, with a particular emphasis on those generated by NIH Common Fund programs. This platform has the potential to be highly useful for experimental biologists who often struggle with fetching and analyzing gene sets across diverse NIH web portals.

Below are my comments on the manuscript:

1. "Only Accept Valid Human Gene Symbols" Option

The "Only Accept Valid Human Gene Symbols" option, if not selected at the beginning, can result in certain operations during the AUGMENT phaseâ€”such as selecting specific gene setsâ€—failing. Highlighting this option prominently at the start would prevent invalid user operations and enhance usability.

2. Protein-Protein Interaction (PPI) Method for Augmentation

GeneSetCart offers three augmentation methods: PPI, Co-Expression, and Literature Co-Mentions. However, the PPI method seems to provide minimal augmentation compared to the other two. For example, applying PPI to the "GSEA: HALLMARK_IL2_STAT5_SIGNALING" human gene set added only three new genes, resulting in 196 genes, compared to 376 and 379 genes augmented by Co-Expression and Literature Co-Mentions, respectively.

- The authors should justify the inclusion of PPI as an augmentation method, especially given its relatively limited contribution.
- Alternatively, they could consider integrating enrichment tools specifically tailored for network data in the ANALYSIS phase, where high-quality protein-protein interaction data could play a more critical role.

3. Analysis Tools

GeneSetCart includes a wide range of analysis tools, such as CFDE-GSE, Enrichr, Enrichr-KG, Rummagene, RummaGEO, ChEA3, KEA3, SigCOM LINCS, L2S2, and PFOCRummage. However:

- Switching between these tools requires repeated manual navigation, which could be streamlined.
- Applying all tools generates a large volume of results, which might be overwhelming for experimental biologists to interpret.

Recommendation: Consider providing a batch-run option and integrating the results in an organized, user-friendly format to improve accessibility and usability.

4. Report Generation Time

The platform's report generation time varies significantly depending on the size of the input gene set. Larger gene sets result in substantially longer generation times. Given the potential use of the platform with large-scale datasets, the authors should assess and report the platform's scalability.

5. Report Generation by GPT-40
The GPT-40 feature for generating explanatory hypotheses is currently restricted to the COMMON FUND GENE SET CROSSING module. However, users cannot apply this functionality to their own gene set intersections, limiting its broader applicability.
- The authors should clarify the rationale behind this restriction.
- Additionally, they should discuss whether this functionality can be extended to user-defined gene sets to enhance usability and versatility.

Methods

Are the methods appropriate to the aims of the study, are they well described, and are necessary controls included? Choose an item.

Conclusions

Are the conclusions adequately supported by the data shown? Choose an item.

Reporting Standards

Does the manuscript adhere to the journal's guidelines on minimum standards of reporting? Choose an item.

Choose an item.

Statistics

Are you able to assess all statistics in the manuscript, including the appropriateness of statistical tests used? Choose an item.

Quality of Written English

Please indicate the quality of language in the manuscript: Choose an item.

Declaration of Competing Interests

Please complete a declaration of competing interests, considering the following questions:

- Have you in the past five years received reimbursements, fees, funding, or salary from an organisation that may in any way gain or lose financially from the publication of this manuscript, either now or in the future?
- Do you hold any stocks or shares in an organisation that may in any way gain or lose financially from the publication of this manuscript, either now or in the future?
- Do you hold or are you currently applying for any patents relating to the content of the manuscript?

- Have you received reimbursements, fees, funding, or salary from an organization that holds or has applied for patents relating to the content of the manuscript?
- Do you have any other financial competing interests?
- Do you have any non-financial competing interests in relation to this paper?

If you can answer no to all of the above, write 'I declare that I have no competing interests' below. If your reply is yes to any, please give details below.

I declare that I have no competing interests

I agree to the open peer review policy of the journal. I understand that my name will be included on my report to the authors and, if the manuscript is accepted for publication, my named report including any attachments I upload will be posted on the website along with the authors' responses. I agree for my report to be made available under an Open Access Creative Commons CC-BY license (http://creativecommons.org/licenses/by/4.0/). I understand that any comments which I do not wish to be included in my named report can be included as confidential comments to the editors, which will not be published.

Choose an item.

To further support our reviewers, we have joined with Publons, where you can gain additional credit to further highlight your hard work (see: https://publons.com/journal/530/gigascience). On publication of this paper, your review will be automatically added to Publons, you can then choose whether or not to claim your Publons credit. I understand this statement.

Yes Choose an item.
